# Impact of common cardio-metabolic risk factors on fatal and non-fatal cardiovascular disease in Latin America and the Caribbean: an individual-level pooled analysis of 31 cohort studies

**DOI:** 10.1016/j.lana.2021.100068

**Published:** 2021-12

**Authors:** Cohorts Consortium of Latin America and the Caribbean (CC-LAC), Rodrigo M. Carrillo-Larco, Dalia Stern, Ian R. Hambleton, Anselm Hennis, Mariachiara Di Cesare, Paulo Lotufo, Catterina Ferreccio, Vilma Irazola, Pablo Perel, Edward W. Gregg, J. Jaime Miranda, Majid Ezzati, Goodarz Danaei, Carlos A. Aguilar-Salinas, Ramón Alvarez-Váz, Marselle B. Amadio, Cecilia Baccino, Claudia Bambs, João Luiz Bastos, Gloria Beckles, Antonio Bernabe-Ortiz, Carla D.O. Bernardo, Katia V. Bloch, Juan E. Blümel, Jose G. Boggia, Pollyanna K. Borges, Miguel Bravo, Gilbert Brenes-Camacho, Horacio A Carbajal, Maria S. Castillo Rascon, Blanca H. Ceballos, Veronica Colpani, Jackie A. Cooper, Sandra Cortes, Adrian Cortes-Valencia, Roberto S. Cunha, Eleonora d'Orsi, William H. Dow, Walter G. Espeche, Flavio D. Fuchs, Sandra C. Fuchs, Suely G.A. Gimeno, Donaji Gomez-Velasco, David A. Gonzalez-Chica, Clicerio Gonzalez-Villalpando, María-Elena Gonzalez-Villalpando, Gonzalo Grazioli, Ricardo O. Guerra, Laura Gutierrez, Fernando L Herkenhoff, Andrea RVR Horimoto, Andrea Huidobro, Elard Koch, Martin Lajous, Maria Fernanda Lima-Costa, Ruy Lopez-Ridaura, Alvaro C.C. Maciel, Betty S. Manrique-Espinoza, Larissa P. Marques, Jose G. Mill, Leila B. Moreira, Oscar M. Muñoz, Lariane M. Ono, Karen Oppermann, Karina M. Paiva, Sergio V. Peixoto, Alexandre C. Pereira, Karen G. Peres, Marco A. Peres, Paula Ramírez-Palacios, Cassiano R. Rech, Berenice Rivera-Paredez, Nohora I. Rodriguez, Rosalba Rojas-Martinez, Luis Rosero-Bixby, Adolfo Rubinstein, Alvaro Ruiz-Morales, Martin R. Salazar, Aaron Salinas-Rodriguez, Jorge Salmerón, Ramon A. Sanchez, Nelson A.S. Silva, Thiago L.N. Silva, Liam Smeeth, Poli M. Spritzer, Fiorella Tartaglione, Jorge Tartaglione, Rafael Velázquez-Cruz

**Affiliations:** a Imperial College London, UK; b National Institute of Public Health, Mexico; c The University of the West Indies, Barbados; d Pan American Health Organization, USA; e Middlesex University, UK; f University of São Paulo, Brazil; g Pontificia Universidad Católica de Chile, Chile; h Institute for Clinical Effectiveness and Health Policy, Argentina; i London School of Hygiene and Tropical Medicine, UK; j Imperial College London, UK; k Universidad Peruana Cayetano Heredia, Peru; l Imperial College London, UK; m Harvard T.H. Chan School of Public Health, USA; o Instituto Nacional de Ciencias Médicas y Nutrición, México; p Universidad de la República, Uruguay; q Centro Universitario Senac Santo Amaro, Brazil; r Universidad de la República, Uruguay; s Pontificia Universidad Católica de Chile, Chile; t Universidade Federal de Santa Catarina, Brazil; u Centers for Disease Control and Prevention, USA; v Universidad Peruana Cayetano Heredia, Perú; w The University of Adelaide, Australia; x Universidade Federal do Rio de Janeiro (UFRJ), Brazil; y Universidad de Chile, Chile; z Universidad de la República, Uruguay; aa Universidade Estadual de Ponta Grossa, Brazil; bb MELISA Institute, Chile; cc Universidad de Costa Rica, Costa Rica; dd Universidad Nacional de la Plata, Argentina; ee Universidad Nacional de Misiones, Argentina; ff Hospital Dr Ramon Madariaga, Argentina; gg Federal University of Rio Grande do Sul, Brazil; hh Queen Mary University of London, UK; ii Pontificia Universidad Católica de Chile, Chile; jj National Institute of Public Health, Mexico; kk Federal University of Espírito Santo, Brazil; ll Universidade Federal de Santa Catarina, Brazil; mm University of California, Berkeley, USA; nn Universidad Nacional de la Plata, Argentina; oo Universidade Federal do Rio Grande do Sul, Brazil; pp Universidade Federal do Rio Grande do Sul, Brazil; qq Universidad Federal de São Paulo, Brazil; rr Instituto Nacional de Ciencias Médicas y Nutrición, México; ss The University of Adelaide, Australia; tt Instituto Nacional de Salud Pública, México; uu Centro de Estudios en Diabetes A.C., México; vv Hospital Churruca Visca, Argentina; ww Federal University of Rio Grande do Norte, Brazil; xx Institute for Clinical Effectiveness and Health Policy, Argentina; yy Federal University of Espírito Santo, Brazil; zz University of São Paulo, Brazil; aaa Universidad Católica del Maule, Chile; bbb MELISA Institute, Chile; ccc Harvard T.H. Chan School of Public Health, USA; ddd National Institute of Public Health, Mexico; eee Oswaldo Cruz Foundation, Brazil; fff National Institute of Public Health, Mexico; ggg Federal University of Rio Grande do Norte, Brazil; hhh National Institute of Public Health, Mexico; iii Universidade Federal de Santa Catarina, Brazil; jjj Federal University of Espírito Santo, Brazil; kkk Universidade Federal do Rio Grande do Sul, Brazil; lll Pontificia Universidad Javeriana, Hospital Universitario San Ignacio, Colombia; mmm Universidade Federal do Paraná, Brazil; nnn Passo Fundo University, Brazil; ooo Universidade Federal de Santa Catarina, Brazil; ppp Oswaldo Cruz Foundation, Brazil; qqq University of São Paulo, Brazil; rrr NDRIS/NDCS Duke-NUS Medical School, Singapore; sss NDRIS/NDCS Duke-NUS Medical School, Singapore; ttt IMSS Epidemiology and Health Services Research Unit, Mexico; uuu Universidade Federal de Santa Catarina, Brazil; vvv National Autonomous University of Mexico, Mexico; www Clinica de Marly, Colombia; xxx Instituto Nacional de Salud Pública, México; yyy Universidad de Costa Rica, Costa Rica; zzz Institute for Clinical Effectiveness and Health Policy, Argentina; aaaa Pontificia Universidad Javeriana, Colombia; bbbb Universidad Nacional de la Plata, Argentina; cccc National Institute of Public Health, Mexico; dddd National Autonomous University of Mexico, Mexico; eeee Universidad Nacional de Misiones, Argentina; ffff Universidade Federal do Rio de Janeiro (UFRJ), Brazil; gggg Universidade federal do Rio de Janeiro (UFRJ), Brazil; hhhh London School of Hygiene & Tropical Medicine, UK; iiii Federal University of Rio Grande do Sul, Brazil; jjjj Hospital Churruca Visca, Argentina; kkkk Hospital Churruca Visca, Argentina; llll National Institute of Genomic Medicine (INMEGEN), Mexico

## Abstract

**Background:**

Estimates of the burden of cardio-metabolic risk factors in Latin America and the Caribbean (LAC) rely on relative risks (RRs) from non-LAC countries. Whether these RRs apply to LAC remains unknown.

**Methods:**

We pooled LAC cohorts. We estimated RRs per unit of exposure to body mass index (BMI), systolic blood pressure (SBP), fasting plasma glucose (FPG), total cholesterol (TC) and non-HDL cholesterol on fatal (31 cohorts, n=168,287) and non-fatal (13 cohorts, n=27,554) cardiovascular diseases, adjusting for regression dilution bias. We used these RRs and national data on mean risk factor levels to estimate the number of cardiovascular deaths attributable to non-optimal levels of each risk factor.

**Results:**

Our RRs for SBP, FPG and TC were like those observed in cohorts conducted in high-income countries; however, for BMI, our RRs were consistently smaller in people below 75 years of age. Across risk factors, we observed smaller RRs among older ages. Non-optimal SBP was responsible for the largest number of attributable cardiovascular deaths ranging from 38 per 100,000 women and 54 men in Peru, to 261 (Dominica, women) and 282 (Guyana, men). For non-HDL cholesterol, the lowest attributable rate was for women in Peru (21) and men in Guatemala (25), and the largest in men (158) and women (142) from Guyana.

**Interpretation:**

RRs for BMI from studies conducted in high-income countries may overestimate disease burden metrics in LAC; conversely, RRs for SBP, FPG and TC from LAC cohorts are similar to those estimated from cohorts in high-income countries.

**Funding:**

Wellcome Trust (214185/Z/18/Z)

**Research In Context**

**Evidence before this study**

The search query ("Latin America" AND "Caribbean") AND ("relative risks" OR "population attributable fraction OR "PAF") AND ("body mass index" OR "BMI" OR "blood pressure" OR "total cholesterol" OR "fasting glucose") did not retrieve any results in PubMed (June 14^th^ 2021). It is well known that Latin America and the Caribbean has not had large multi-country cohort studies or cohort pooling projects. Before this work, the evidence about long-terms effects of cardio-metabolic risk factors in Latin America and the Caribbean was informed by cohorts conducted in North America, Europe and Asia.

**Added value of this study**

This work pooled data from several Latin American and the Caribbean cohorts and examined the relative risks of established cardio-metabolic risk factors for cardiovascular outcomes. We found that the relative risks for systolic blood pressure, fasting glucose and total cholesterol, are similar to those reported by cohort pooling projects carried out in other world regions (e.g., Asia-Pacific Cohort Studies Collaboration, Prospective Studies Collaboration and Emerging Risk Factors Collaboration); however, for body mass index, the relative risks were slightly smaller in Latin America and the Caribbean. We used the relative risks herein derived to estimate the mortality attributable to non-optimal levels of the selected cardio-metabolic risk factors. We estimated the largest attributable cardiovascular deaths due to non-optimal systolic blood pressure and non-HDL cholesterol. These risk factors had a larger impact on cardiovascular deaths in the Caribbean, as well as in Southern and Tropical sub-regions.

**Implications for all the available evidence**

Our results support using global relative risks for systolic blood pressure, fasting glucose and total cholesterol in Latin America and the Caribbean; for body mass index, however, it seems reasonable to use the relative risks herein proposed. Global relative risks for body mass index may overestimate disease burden metrics in Latin America and the Caribbean.

Alt-text: Unlabelled box

## 1 Introduction

Cardiovascular diseases are the leading causes of death globally [1], and the main causes of these deaths are a set of well-known cardio-metabolic risk factors such as high blood pressure, overweight/obesity, diabetes and dyslipidemias [2], [3], [4]. Supporting evidence on the impact of cardio-metabolic risk factors on cardiovascular diseases has mostly come from cohort pooling collaborations [5], including the Asia-Pacific Cohort Studies Collaboration [6,7], the Prospective Studies Collaboration [8], [9], [10], and the Emerging Risk Factors Collaboration [11], [12], [13]. The results of these collaborations have been used to attribute disease burden to risk factors globally, providing inputs for surveillance and monitoring of cardio-metabolic risk factors and diseases.

These collaborations have little representation from Latin America and the Caribbean (LAC) [14], a vast region with unique characteristics in terms of non-communicable diseases such as diabetes and raised blood pressure [15], paired with the fastest rate of transition towards a predominance of urban areas in the developing world [16,17]. Therefore, the findings of these non-LAC collaborations, such as the age-specific relative risks used in global burden of disease estimations, may not apply to LAC countries. In fact, evidence suggests that the association between some risk factors and cardio-metabolic outcomes may be stronger in LAC compared with other world regions [4,18], possibly due to different levels of access to health care [19,20], differences in the distribution of cardio-metabolic risk factors [15,[21], [22], [23]], or incidence of non-communicable diseases [24], [25], [26]. We identified and pooled prospective cohort studies in LAC to examine the effect of major cardio-metabolic risk factors on cardiovascular outcomes, and to estimate age-specific relative risks for this world region.

## 2 Methods

Details about the Cohorts Consortium of Latin America and the Caribbean (CC-LAC) have been reported detailed elsewhere [27]. We analysed cohort data pooled and harmonized by the CC-LAC [27], a network of health researchers and practitioners in LAC. We have harmonised and pooled approximately population-based cohort data on cardio-metabolic risk factors and outcomes, i.e., participants were not recruited based on cardiovascular diseases (CVD) (e.g., cohort of stroke survivors) or risk factor (e.g., cohort of smokers) history only. Cohort studies were identified through a systematic search and networks of researchers in LAC. We identified 78 approximately population-based cohorts (i.e., did not select participants on the basis of having previous disease) and excluded 31 cohorts that recruited only young participants (e.g., birth cohorts), did not measure exposures/outcomes of interests, or could not be accessed by the original investigators [27]. We accessed 33 cohorts from 13 countries (37% of LAC countries). From these 33 cohorts, 5 included participants who attended a specific health centre [28], [29], [30] or were members of a professional organization such as The Mexican Teachers' Cohort [31] and the Health Workers Cohort Study [32]. The other cohorts sampled individuals from the general population. Individual-level data from each cohort were received by the CC-LAC and were subsequently harmonised and pooled for the present analyses [27].

From the pooled 33 cohorts, we excluded two that did not ascertain either fatal or non-fatal cardiovascular events; we further excluded 18 cohorts that did not ascertain non-fatal cardiovascular events from analysis of fatal- and non-fatal outcomes. Thus, our estimates for fatal cardiovascular events were informed by 31 cohorts with a mean follow-up of 8.8 years (standard deviation = 3.1), while our estimates for fatal and non-fatal cardiovascular events were informed by 13 cohorts with a mean follow-up of 8.5 years (standard deviation = 5.3).

We estimated incidence rate ratios which we will hereafter refer to as relative risks (RRs) and 95% Confidence Intervals (95% CI) for each selected cardio-metabolic risk factor on fatal and non-fatal cardiovascular diseases. We used these RRs to estimate the proportion and number of deaths attributable to each risk factor.

### 2.1 Cardiovascular outcomes

We analysed two outcomes separately: i) fatal and non-fatal cardiovascular events and ii) fatal cardiovascular events. Non-fatal cardiovascular events were not analysed alone because of the small number of events in many age groups. Cardiovascular events were identified using data from vital registration systems, clinical records or verbal autopsies, and where relevant adjudicated by each cohort (Supplementary Table 1).

### 2.2 Cardio-metabolic risk factors

The risk factors of interest were systolic blood pressure (SBP, in mmHg), body mass index (BMI, in kg/m^2^), fasting plasma glucose (FPG, in mmol/L), total serum cholesterol (in mmol/L) and non-high-density lipoprotein cholesterol (non-HDL, in mmol/L). These variables were collected following standardised protocols in each cohort. We only examined SBP as the relationship between SBP and CVD outcomes is stronger than that of diastolic blood pressure [8,33].

### 2.3 Statistical analysis

#### 2.3.1 Handling of missing data

BMI was missing in 12% of the pooled observations, while this number for SBP, total cholesterol, FPG and non-HDL cholesterol ranged from 66% to 80% (Supplementary Table 2). We used multiple imputation and fitted the regression models in each of 50 imputed datasets, pooling the estimates following Rubin's rules [34]. In sensitivity analyses, we used a complete-case dataset, and we observed minor differences for non-HDL cholesterol but overall the results were unchanged using multiple imputation (Supplementary Figure 1). Detailed methods on multiple imputation are available in Supplementary Material (pp. 7-8). The main results herein presented are based on the multiple imputation data.

#### 2.3.2 Adjusting for Regression Dilution Bias (RDB)

As the selected risk factors have a natural variability during follow-up, the estimated associations between baseline one-off measures underestimate the effect of “usual” exposure. This is often referred to as regression dilution bias [35,36]. To adjust for this bias, we used data from 10 of our cohorts with repeated risk factor measurements and used standard analytic methods (MacMahon method) [35,36] to calculate correction factors. The estimated correction factors were: 1.10 for BMI, 1.50 for SBP, 1.53 for FPG, 1.75 for total cholesterol, and 1.85 for non-HDL cholesterol. Further details about the RDB methods are available in the expanded methods (Supplementary Material pp. 6-7).

#### 2.3.3 Survival analysis

For each cardio-metabolic risk factor, we fitted a Poisson linear mixed effects regression model for each age group separately (35-44, 45-54, 55-64, 65-74, 75-84, 85+ years) in which the independent variable was the risk factor, adjusted for sex and age at risk (i.e., at follow-up/event) within each age-group. A cohort-specific random intercept was included as well as the natural logarithm of the follow-up time as an offset. The coefficient of each risk factor from this model represents the log-incidence rate ratio for one-unit increase in the risk factor in each 10-year age group. We applied the RDB correction factor to these coefficients (in the log scale). For further details, refer to the extended methods (Supplementary Material pp. 5-11).

To better understand any potential differences in RRs between sub-regions in LAC, we computed RDB-adjusted RRs for fatal cardiovascular diseases for Central America & the Caribbean (Costa Rica, Cuba, Dominican Republic, Puerto Rico and Trinidad & Tobago) versus South America (Argentina, Brazil, Chile, Colombia, Peru, Uruguay and Venezuela). We included Mexico in the former group to preserve geographic proximity. To compare our results with a previous analysis of cohort data pooling studies that reported RRs for fatal ischaemic heart disease and stroke subtypes separately [5], we weighted their estimated RRs by the relative prevalence of these outcomes in LAC.

#### 2.3.4 Quantifying the population-level impact of risk factors

Following a comparative risk assessment approach [37], we estimated the population attributable fraction (PAF) for each risk factor on cardiovascular deaths in 35 countries of the region comparing the current mortality burden to the one that would have been observed if the mean levels in the population were optimal (Supplementary Material p. 9). The optimal levels in the population were derived from previous analyses of global burden of disease [5]. Current mean levels of BMI, SBP, total cholesterol and non-HDL cholesterol for each country and by five-year age group and sex, were extracted from the NCD-RisC (http://ncdrisc.org/) [15,22,23]. This information was not available for FPG, therefore, we did not include non-optimal glucose in these analyses. The number of cardiovascular deaths for the year 2019 was extracted from the estimates provided in the Global Burden of Disease (GBD) Study [38]. We used the RRs herein estimated (10-year age groups), and we interpolated them into 5-year age groups (Supplementary Figure 2 and Supplementary Table 7) [5]. We used the same age-specific RRs for men and women across countries in LAC, as we found no evidence of different RRs by sex. We estimated crude attributable death rates per 100,000 person-years by multiplying PAFs by total CVD deaths and dividing by the adult population of each country, which was also extracted from the GBD Study. Further details about the comparative risk assessment are available in the expanded methods (Supplementary Material pp. 8-9). Countries for which we made estimates are those in common between the GBD Study and the NCD-RisC (35 countries and territories in LAC).

### 2.4 Risk of bias in each study

We evaluated three sources of bias. First, selection bias due to enrolment of participants. The risk of selection bias in these cohorts is rather small because inclusion in the study is unlikely to be simultaneously related to exposure and outcome. Second, measurement bias. As explained above and in Supplementary Material p. 04, major variables of interest were measured except for BMI which was self-reported in one cohort [31,39]. These variables are commonly measured in cardiovascular cohorts and were measured following standard procedures. Regarding the outcomes, we did not pool cohorts in which this information was not verified using links to vital registration data or adjudication (Flow Diagram on Supplementary Material p. 04). Third, confounding. We adjusted for age, sex and cohort in all analyses as we were interested in comparing the magnitude of our RRs with those of other global pooling studies which used the same set of potential confounders [5].

#### 2.4.1 Role of the funding source

The funder of the study had no role in study design, data collation, analysis, interpretation, or writing of the report. RMC-L and GD had full access to the data in the study. RMC-L and GD had final responsibility for the decision to submit for publication.

## 3 Results

In the group of cohorts analysed for fatal outcomes, women were younger than men (46.1 vs 55.7 years), while in the second set of cohorts the age was more alike (52.7 vs 52.1 years). In both sets of cohorts (i.e., analysis for fatal as well as for fatal and non-fatal outcomes), women had higher BMI than men (27.4 kg/m^2^ vs 26.2 kg/m^2^ and 28.6 kg/m^2^ vs 26.4 kg/m^2^); mean total cholesterol was also higher in women (5.4 mmol/L vs 5.2 mmol/L and 5.3 mmol/L vs 5.2 mmol/L). The average non-HDL cholesterol was slightly higher among men than women in both sets of cohorts (4.1 mmol/L vs 4.2 mmol/L and 4.1 mmol/L vs 4.2 mmol/L). In both sets of cohorts, mean SBP was higher among men than women (131 mmHg vs 134 mmHg and 128 mmHg vs 133 mmHg; Supplementary Table 3B).

For fatal outcomes, the 31 selected cohort studies contributed with 168,287 eligible participants aged 20 years old and over. More than four fifths were women (83.7%) and they were on average 47.7 (standard deviation (SD)= 12.2) years old at baseline. The mean BMI was 27.2 kg/m^2^ (SD = 4.8), the average SBP was 131 mmHg (SD = 22.1), the mean FPG was 5.5 mmol/L (SD = 1.9), the mean total cholesterol was 5.3 mmol/L (SD = 1.3), and the average non-HDL cholesterol was 4.2 mmol/L (SD = 1.3) (Supplementary Table 3). The mean follow-up was 8.9 (SD = 3.1) years. In the 31 cohorts analysed for fatal outcomes we observed, 1,710 events (116 (95% CI: 111-122) per 100,000 person-years).

**Figure 1:**
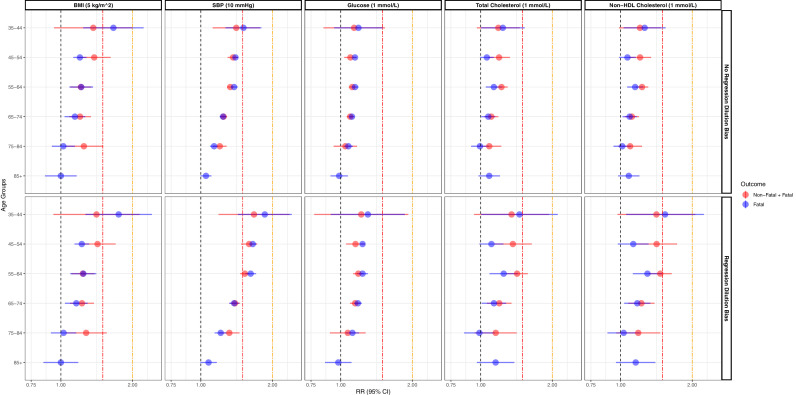
Age-specific relative risks for fatal and fatal plus non-fatal cardiovascular disease associated with usual levels of selected cardio-metabolic risk factors. While the upper panel shows estimates without accounting for regression dilution bias, the lower panel shows estimates accounting for regression dilution bias; all estimates were adjusted by sex and age (within each age group). Age groups based on age at risk. Estimates for fatal plus non-fatal events included only the first five age groups (insufficient observations in the eldest age group). RR: relative risk; 95% CI: 95% confidence interval; BMI: body mass index; SBP: systolic blood pressure. The red vertical line at relative risk = 1.5 and the orange vertical line at relative risk = 2.0 on the X-axis.

We observed an age gradient in the magnitude of the RDB-adjusted RRs across all cardio-metabolic risk factors for fatal cardiovascular outcomes, with smaller RRs in older ages; this pattern was less clear for total cholesterol and non-HDL cholesterol (Figure 1, Supplementary Table 5). The magnitude of the RRs in the youngest group (35-44) was at or above 1.3 (Figure 1, Supplementary Table 5), with the largest estimate for SBP on fatal cardiovascular events (RR = 1.9, 95% CI: 1.4-2.4); conversely, the RRs for FPG in the youngest age group was 1.3 (95% CI: 0.9-1.9) (Figure 1, Supplementary Table 5).

**Figure 2:**
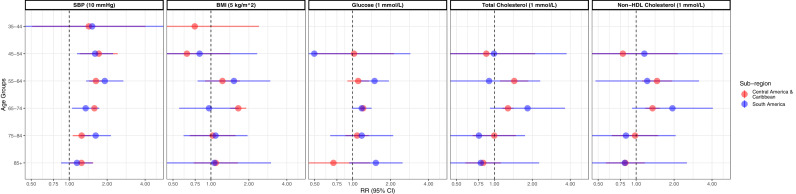
Age-specific relative risks for fatal cardiovascular disease associated with usual levels of selected cardio-metabolic risk factors by sub-regions. All models were adjusted by sex and age (within each age group). Age groups based on age at risk (i.e. at outcome). RR: relative risk; 95% CI: 95% confidence interval; BMI: body mass index; SBP: systolic blood pressure. Only results adjusted for regression dilution bias are presented. Results as per multiple imputation. Insufficient observations to reliably compute these risk estimates for fatal plus non-fatal cardiovascular events. RR: relative risk; 95% CI: 95% confidence interval.

In regional sub-group analyses, we did not observe substantial differences in the magnitude of the RRs for fatal outcomes. In both sub-regions, we could not observe a clear age gradient with larger RRs in younger groups; except for SBP where there was an age gradient from age 45 years (Figure 2, Supplementary Table 6).

For fatal and non-fatal outcomes, the 13 cohorts contributed with 27,554 eligible individuals. Almost two thirds were men (64.1%), and the mean age was 52.3 (10.5) years. The mean BMI, SBP, FPG and total cholesterol and non-HDL cholesterol was: 27.2 kg/m^2^ (SD = 5.1), 131 mmHg (SD = 21.0), 5.3 mmol/L (SD = 1.7), 5.3 mmol/L (SD = 1.1) and 4.1 mmol/L (SD = 1.1), respectively (Supplementary Table 3). The mean follow-up was 8.5 (SD = 5.3) years. In the 13 cohorts analysed for fatal and non-fatal events, there were 577 non-fatal events (246 (95% CI: 227-267) per 100,000 person-years) and 677 fatal events (288 (95% CI: 267-311) per 100,000 person-years).

We observed an age gradient in the magnitude of the RDB-adjusted RRs across all cardio-metabolic risk factors for fatal and non-fatal cardiovascular outcomes, with smaller RRs in older ages (Figure 1, Supplementary Table 5). The magnitude of the RRs in the youngest group (35-44) was at or above 1.2 (Figure 1, Supplementary Table 5). In the youngest age group, the largest RR for fatal and non-fatal cardiovascular outcomes was observed for SBP (RR = 1.7, 95% CI: 1.2-2.4); conversely, the smallest RR was for FPG (RR = 1.2, 95% CI: 0.8-1.9) (Figure 1, Supplementary Table 5).

**Figure 3:**
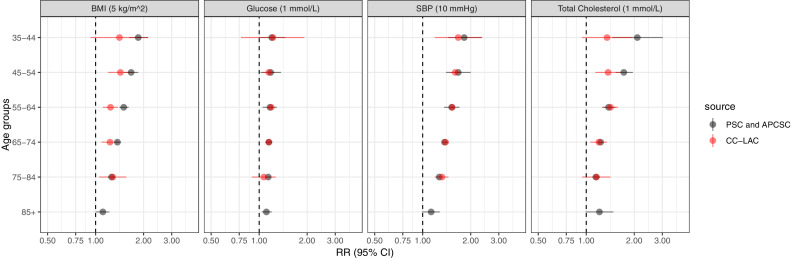
Relative risks from the pooled analysis of PSC and APCSC [5] compared with those from LAC cohort pooling. Estimates from LAC cohorts are those adjusted by regression dilution bias and based on multiple imputation for fatal and non-fatal cardiovascular outcomes. Our estimates for fatal plus non-fatal outcomes were computed for the first five age groups only (insufficient observations in the oldest age group). PSC: Prospective Studies Collaboration; APCSC: Asia Pacific Cohort Studies Collaboration. RR: relative risk; 95% CI: 95% confidence interval.

The age-specific RRs for fatal and non-fatal CVD for SBP and FPG were remarkably similar to those reported from cohorts mostly conducted in high-income countries (Figure 3) [5]. For TC, our RRs appeared to be smaller for the two youngest age groups, though these differences were statistically insignificant (Figure 3). For BMI, the RRs were consistently smaller in magnitude for participants younger than 75 years old, with the largest difference for those in the age group 55-64 years (Figure 3): 1.24 (95% CI: 1.11-1.38) vs. 1.50 (95% CI: 1.41-1.61).

Non-optimal SBP was responsible for the largest proportion of attributable cardiovascular deaths across countries, with a proportional effect ranging from 30.7% among Cuban women to 58.0% among men from Grenada. The second largest proportion for attributable CVD mortality was due to non-optimal non-HDL cholesterol, which proportional effect varied between 13.9% (Chile, women) and 31.2% (Guyana, women). The proportional effect of non-optimal BMI and total cholesterol were smaller. For BMI the proportional effect ranged from 6.1% in women from Cuba to 19.6% in men from Saint Kitts and Nevis, whereas for total cholesterol these numbers were 4.4% (Guatemala, men) and 18.1% (Guyana, women; Supplementary Figure 3).

**Figure 4:**
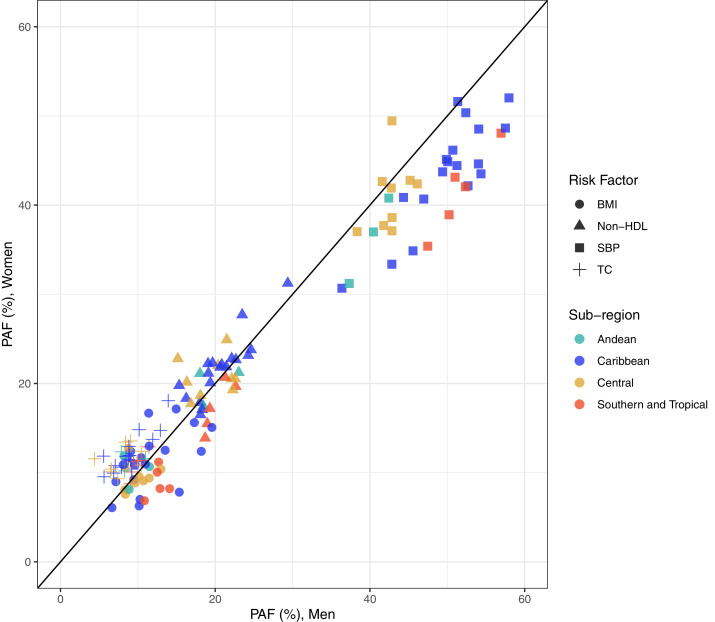
Population attributable fraction (PAF, %) in women compared to men by risk factor and sub-regions. BMI: body mass index; SBP: systolic blood pressure.

The proportional effect of non-optimal total cholesterol tended to be larger among women than among men, as was observed in 34 countries (Figure 4); the largest absolute difference between women and men was observed in Guatemala (11.6% in women vs 4.4% in men; Supplementary Figure 3). On the other hand, the proportional effect of non-optimal SBP was higher among men than among women in most countries (Figure 4), with the largest difference in Uruguay (47.5% in men vs 35.4% in women; Supplementary Figure 3).

Over half a million deaths (502,913 (95% credible interval = 340,637-653,242) out of a total of 1,094,795 CVD deaths in LAC) were attributable to non-optimal SBP in 2019. The second largest effect was estimated for non-optimal non-HDL cholesterol at 224,118 (95% credible interval = 83,755-388,176) deaths and the lowest for non-optimal BMI at 119,498 (95% credible interval = 61,201-200,824) (Table 1). Brazil, Mexico and Argentina, in that order, had the largest numbers of attributable deaths across the four risk factors for both women and men; Colombia displaced Argentina from the third place regarding non-HDL cholesterol in women (Supplementary Figure 3).

**Table 1:** Number of cardiovascular deaths in 2019 attributable to each risk factor by sub-region and sex in Latin America and the Caribbean

**Region**	**Sex**	**BMI**			**SBP**			**TC**			**Non-HDL**		
		**Estimate**	**Lower CI**	**Upper CI**	**Estimate**	**Lower CI**	**Upper CI**	**Estimate**	**Lower CI**	**Upper CI**	**Estimate**	**Lower CI**	**Upper CI**
**All ages (ages 20 and above)**													
Andean Latin America	Men	3299	1605	5687	13590	8107	18597	3118	562	7135	6841	2548	11916
Andean Latin America	Women	3124	1602	5450	11216	5757	16342	3532	622	8193	6189	2025	11376
Caribbean	Men	6176	2832	10630	27503	16887	36885	6178	1123	13847	12863	5229	21835
Caribbean	Women	5134	2312	9475	24135	12787	34567	7710	1593	17115	13125	4588	23590
Central Latin America	Men	21147	11158	34447	81481	53892	107084	16548	3844	35438	39095	15439	65842
Central Latin America	Women	16734	8649	28854	67962	40603	93829	20079	4416	43640	35646	11593	64701
Southern and Tropical Latin America	Men	36418	19372	58019	155845	121477	187436	30617	9504	58140	61208	25953	99449
Southern and Tropical Latin America	Women	27466	13672	48261	121181	81127	158501	32048	7881	66512	49151	16381	89467
**Premature (below age 70)**													
Andean Latin America	Men	2659	1494	3920	6072	3905	7854	1851	473	3519	3886	2003	5631
Andean Latin America	Women	2390	1474	3335	3466	1848	4883	1580	495	2855	2813	1490	4051
Caribbean	Men	5136	2651	7880	14327	9244	18297	3828	930	7373	7824	4119	11345
Caribbean	Women	3932	2104	5920	9617	5259	13180	3697	1248	6596	6342	3397	9102
Central Latin America	Men	17209	10396	24328	40272	28791	49875	10278	3269	18462	22925	12156	32755
Central Latin America	Women	12342	7816	16888	21806	14077	28469	8553	3340	14340	15098	8294	21297
Southern and Tropical Latin America	Men	29324	17847	41325	83297	69701	94929	19949	7806	33399	38343	20426	54830
Southern and Tropical Latin America	Women	19765	12201	27444	41732	31403	50817	14050	5875	22882	22272	11925	31983
**Premature-to-all-ages ratio (%)**													
Andean Latin America	Men	80.60	93.13	68.92	44.68	48.16	42.23	59.37	84.25	49.33	56.81	78.60	47.26
Andean Latin America	Women	76.52	92.02	61.19	30.91	32.10	29.88	44.74	79.54	34.85	45.45	73.56	35.61
Caribbean	Men	83.16	93.61	74.13	52.09	54.74	49.61	61.97	82.80	53.24	60.82	78.78	51.96
Caribbean	Women	76.59	90.99	62.47	39.85	41.12	38.13	47.95	78.35	38.54	48.32	74.04	38.59
Central Latin America	Men	81.38	93.17	70.62	49.42	53.42	46.58	62.11	85.04	52.10	58.64	78.74	49.75
Central Latin America	Women	73.75	90.36	58.53	32.09	34.67	30.34	42.60	75.62	32.86	42.35	71.54	32.92
Southern and Tropical Latin America	Men	80.52	92.13	71.23	53.45	57.38	50.65	65.16	82.14	57.45	62.64	78.71	55.13
Southern and Tropical Latin America	Women	71.96	89.25	56.86	34.44	38.71	32.06	43.84	74.54	34.40	45.31	72.80	35.75

BMI: body mass index; SBP: systolic blood pressure; TC: total cholesterol; Non-HDL: non-HDL cholesterol; CI: 95% credible interval. All ages included observations aged ≥20 years, whereas premature refers to ages between 20 and 69 years. The premature-to-all-ages ratio quantifies the ratio of the estimated attributable deaths below age 70 to the corresponding estimate for all ages expressed as a percentage.

Most of the cardiovascular disease deaths attributable to non-optimal BMI were premature (<70 years of age; Table 1), ranging from 72% among women in Southern and Tropical Latin America, to 83% among men in the Caribbean (Table 1). On the other extreme, the 44% of all cardiovascular disease deaths attributable to non-optimal SBP was premature; this ranged from 31% (women in Andean Latin America) to 54% (men in Southern and Tropical Latin America; Table 1).

**Figure 5:**
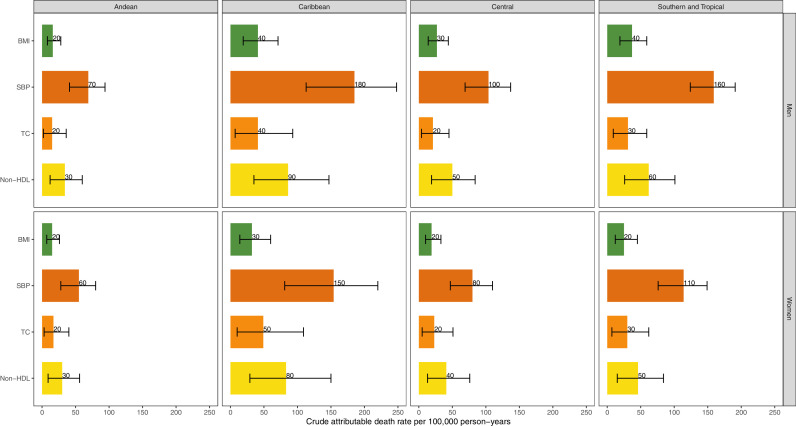
Crude attributable death rates per 100,000 person-years by risk factors, sub-regions and sex. Results at the sub-region level are mean averaged accounting for the population size of the countries within each sub-region. BMI: body mass index; SBP: systolic blood pressure; TC: total cholesterol. The vertical lines along the numbers at the top of the bars represent the 95% credible interval.

Across the four sub-regions, the crude attributable death rate per 100,000 person-years due to non-optimal risk factors was consistently larger among men for BMI, SBP and non-HDL cholesterol (Figure 5). Consistently across all sub-regions, non-optimal SBP was responsible for the largest number of attributable deaths for both men and women. Similarly, non-optimal non-HDL cholesterol always ranked second (Figure 5).

**Figure 6:**
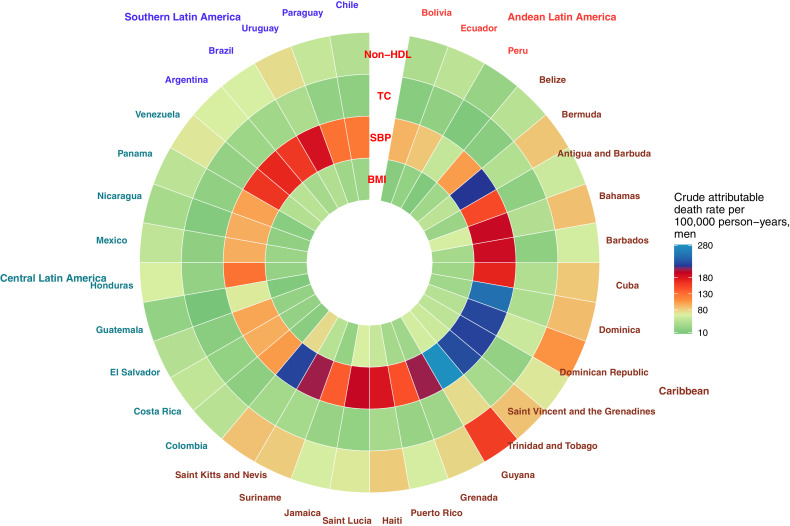

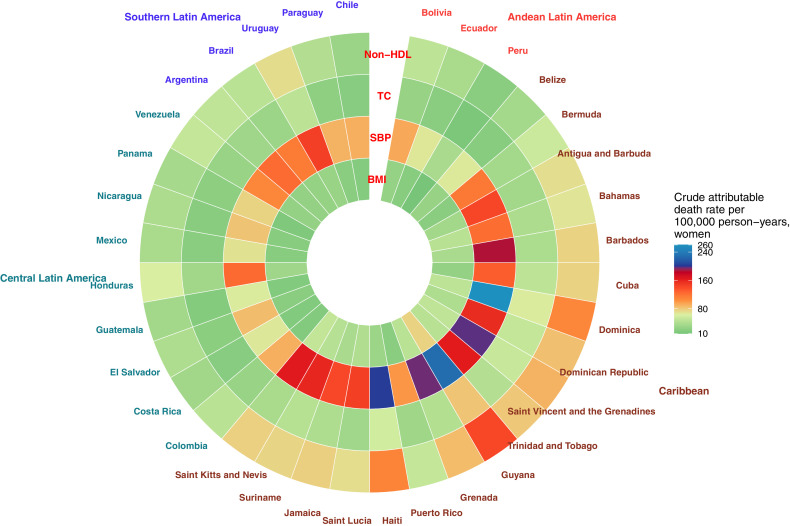
Crude attributable death rates per 100,000 person-years by risk factor and country in Latin America and the Caribbean in men (A) and women (B). Countries are clustered within sub-regions (Andean Latin America, the Caribbean, Central Latin America as well as Southern Latin America). Colour scale allows comparison within each wheel (risk factor) as well as within each column (country).

For men, country-specific attributable death rates to non-optimal SBP (Figure 6A, Supplementary Figure 3), was the smallest in Peru (54 per 100,000 person-years) and Guatemala (67), and the largest in Dominica (249) and Guyana (282). For non-optimal non-HDL cholesterol, we observed the smallest attributable death rates in Guatemala (25) and Peru (27), and the largest in Dominican Republic (107) and Guyana (158). Finally, for BMI, the lowest attributable death rates were observed in Peru (13) and Guatemala (14), and the largest in Saint Lucia (64) and Saint Kitts and Nevis (75).

For women (Figure 6B, Supplementary Figure 3), the smallest attributable death rate to non-optimal SBP was estimated in Peru (38 per 100,000 person-years) and Guatemala (64), while Guyana (228) and Dominica (261) had the largest rates. For non-HDL cholesterol, the lowest attributable rates were observed in Peru (21) and Chile (30), while the largest rates were in Haiti (109) and Guyana (142). For non-optimal BMI, the lowest attributable death rates were observed in Peru (10) and Panama (14), whilst the largest rates were in Saint Kitts and Nevis (51) and Guyana (76).

## 4 Discussion

Benefiting from a unique database of pooled individual-level data from 31 cohort studies in 13 LAC countries [27], we estimated age-specific RRs for major cardiovascular disease risk factors. We observed smaller RRs for BMI compared with those used in the global estimates of disease burden based on cohort collaborations originating mostly in high-income countries. We observed an age gradient whereby young people had higher RRs than older individuals. Our results suggested that the RRs did not differ between Central America & the Caribbean sub-region compared with South America. The largest attributable CVD deaths across the selected risk factors were due to non-optimal SBP, followed by non-HDL cholesterol. These risk factors had a much larger impact on cardiovascular deaths in the Caribbean and Southern and Tropical sub-regions.

The age gradient of the estimated RRs in our analysis is consistent with prior pooled analysis of large cohort studies [2,5,9,10,13,40]. The magnitude of age-specific RRs was similar in our analyses compared with prior pooling projects of cohorts in high-income countries and those of the Asia-Pacific region [5]; however, for BMI, our estimated RRs were smaller for many age groups below the age of 75, particularly for people aged 55-64. The reported RRs for BMI from the Prospective Studies Collaboration, and the Asia Pacific Cohort Studies Collaboration did not account for RDB [10,41]. Adjusting for RDB, would have led to even higher RRs compared with ours. Such similarity may reflect the same underlying biology of these risk factors and lack of major modifications by lifestyle or environmental risk factors that do differ across regions. In fact, where patterns or lengths of exposure matter as it is the case for smoking and alcohol use, RRs of cardiovascular outcomes differ substantially by region [25]. In contrast, the observed differences in RRs for BMI may be explained by the shorter duration of the weight gain in the LAC region compared with high-income countries. That is, high-income populations have been exposed to non-optimal BMI levels longer than most populations in LAC [22], and are therefore experiencing the larger cumulative harmful effects of BMI on cardiovascular health. Alternatively, the same level of BMI may correspond to a healthier body fat distribution in LAC compared with high-income populations. The current evidence on such a difference in fat distribution at the same level of BMI is mixed [42,43] and further research is needed using larger population-based surveys with measurements of body composition in LAC. Our RRs for non-HDL cholesterol are consistent with a recent analysis of the PURE study, which did not find substantial differences in RRs for non-HDL cholesterol between high-, middle- and low-income countries [4].

The observed differences in RRs for BMI may explain the differences in our estimates of attributable deaths to cardio-metabolic risk factors in LAC versus those reported by the GBD Study, which uses RRs mostly informed by epidemiological studies in high-income countries. For example, we estimated a crude attributable death rate for non-optimal BMI in women in Peru of 10 per 100,000, compared with 18 cardiovascular disease deaths reported by the GBD Study [44] for Guyana we estimated 76 compared to 86 [44]. Notably, the GBD Study risk estimates include other cardiovascular outcomes besides those herein analysed- partly explaining the differences.

Our results show that non-optimal SBP was responsible for the largest number of cardiovascular disease deaths, followed by non-HDL cholesterol, total cholesterol and BMI. This ranking is similar to the one proposed by the GBD Study in 2019, in which SBP ranked first, followed by LDL-Cholesterol, fasting plasma glucose and BMI [45]. This suggests that the ranking based on global risk estimates still apply to LAC, yet the burden attributable to each risk factor may be different. That difference, as herein proposed, may be overestimating the cardiovascular disease mortality attributable to non-optimal BMI in LAC.

Arguably, LAC-based risk estimates -particularly for BMI- provide more valid metrics for countries in LAC to quantify the burden of key cardio-metabolic risk factors. This evidence could allow prioritizing the risk factor(s) with the largest burden, develop policies and interventions to address these priorities, and set up surveillance systems to monitor the progress towards international and local goals. Our results could be taken as parameters upon which goals can be set to reduce cardiovascular burden in LAC and in each country in the region given that metrics to measure the progress and surveillance of cardiovascular diseases were mostly informed by countries outside LAC. Considering the sharp rise in obesity and diabetes in the region [15], despite our evidence that shows lower RRs compared with high-income countries, overweight/obesity remains one of the highest-ranking risk factors for CVD; obesity control and prevention policies should continue to remain top priorities.

Our work has several strengths. The risk estimates are age-specific and were computed following consistent methods using the largest pooled database of cohorts in LAC. We analysed data from 13 countries including at least one from each sub-region in LAC, a work never conducted before. Analysing individual level data, in contrast to published estimates [18,46], allowed us to examine interactions between different variables. The RRs were adjusted for regression dilution bias using LAC data providing the RR of “usual” exposure to risk factors. We used multiple imputation to handle missing data for risk factors at baseline. Nevertheless, we acknowledge several limitations. Due to data availability, we could not study other risk factors such as LDL-cholesterol. Likewise, some outcomes were not available, preventing us from disentangling, for example, ischaemic from haemorrhagic stroke. We were also unable to examine RRs in all sub-regions (e.g., Andean Latin America and southern Latin America) due to the small numbers of events. We therefore only explored risk estimates from South America with those from Central America and the Caribbean, and even in this case, confidence intervals were wide, particularly in the youngest and oldest age groups. Many cohorts did not collect data on non-fatal events (possibly due to the younger age of participants or complexities and costs of identifying and adjudicating non-fatal events), precluding a separate analysis. The limited number of non-fatal events could have also affected the main results (RRs for both fatal and non-fatal CVD), as these estimates could have been mostly driven by fatal events; however, results for fatal outcomes only showed the same age pattern and the RRs had a similar magnitude as those including both fatal and non-fatal events. Mortality risk may also be confounded by health care access and control of non-communicable diseases, variables that were not included in the regression models. A few variables had a large proportion of missing values across cohorts mostly because a subset of cohorts did not include these measurements in their protocol, as opposed to non-response or missing measurements within each cohort. We used modelled estimates of CVD deaths by country, age and sex from the GBD 2019 Study to calculate the attributable number of deaths which makes our results comparable and consistent across countries [38]. However, the estimated CVD mortality may be biased in countries especially if local data is not incorporated in the GBD analyses and/or if modelling assumptions are not valid for a particular region/sub-region. Also in relation to the GBD Study, we acknowledge that GBD deliver estimates for several years whereas we only used their most recent estimates (2019); we focused on the most recent year because we aimed to provide estimates to inform policies and goal setting, rather than showing time patterns. Cohorts herein analysed for fatal outcomes included more women than men; interpretation of these estimates should be made in light of this profile. We only presented results at the country level. Future work should also study cardiovascular disease burden at the subnational level, ideally in all countries in LAC considering its substantial geographical and socioeconomic diversity. We encourage researchers in LAC to use the risk estimates herein reported to conduct subnational analysis of cardiovascular disease burden. We pooled multiple cohorts which included a random sample of the general population or studied specific groups (e.g., The Mexican Teachers' Cohort). We studied cardio-metabolic risk factors (e.g., blood pressure and total cholesterol) which were collected following objective, standard and comparable methods between cohorts. The risk of selection bias is quite low because the probability of being selected in these studies is unlikely to be simultaneously related to the exposure and outcome. Regarding BMI, except for one cohort we used measured weight and height which reduces measurement error; this is method is consistent with other cohort pooling projects.

In conclusion, using data from the first pooling project of cohort studies in LAC we found that RRs of cardiovascular disease per unit increase in blood pressure, glucose and cholesterol are remarkably similar to previous pooling projects that used data mostly from high-income countries. In contrast, we observed smaller age-specific RRs for BMI. The estimated RRs offer region-specific evidence that can be used to update estimates of attributable burden of disease to better inform regional policies and goals. One of the strategic lines of action in Pan American Health Organization's Plan of Action for the Prevention and Control of Non-communicable Diseases in the Americas 2013-2019, was to strengthen country capacity for surveillance on non-communicable diseases and their risk factors [47]. Our results can help improve the validity of such surveillance efforts by emphasizing the use of local data and evidence in prioritizing and implementing CVD prevention programs.

## 5 Contributions

GD, RMCL and ME conceived the study. RMCL curated the data and conducted all analysis with input from GD and ME. All members of the CC-LAC Steering committee contributed to the design of the analysis and interpretation of the results and conclusions. RMCL wrote the first draft of the manuscript and all co-authors contributed to the revisions. GD and RMCL have access to and have verified the underlying data.

## 6 Data sharing

Data is currently only available to CC-LAC collaborators. Expressions of interest to access the CC-LAC data are welcomed and will be handled by the CC-LAC steering committee.

## 7 Cohorts Consortium of Latin America and the Caribbean (CC-LAC)

Steering committee

Rodrigo M Carrillo-Larco (Imperial College London, UK); Dalia Stern (National Institute of Public Health, Mexico); Ian R Hambleton (The University of the West Indies, Barbados); Anselm Hennis (Pan American Health Organization, USA); Mariachiara Di Cesare (Middlesex University, UK); Paulo Lotufo (University of São Paulo, Brazil); Catterina Ferreccio (Pontificia Universidad Católica de Chile, Chile); Vilma Irazola (Institute for Clinical Effectiveness and Health Policy, Argentina); Pablo Perel (London School of Hygiene and Tropical Medicine, UK); Edward W Gregg (Imperial College London, UK); J Jaime Miranda (Universidad Peruana Cayetano Heredia, Peru); Majid Ezzati (Imperial College London, UK); Goodarz Danaei (Harvard T.H. Chan School of Public Health, USA)

Country and Regional Data (* equal contribution; listed alphabetically by surname)

Carlos A Aguilar-Salinas (Instituto Nacional de Ciencias Médicas y Nutrición, México)*; Ramón Alvarez-Váz (Universidad de la República, Uruguay)*; Marselle B Amadio (Centro Universitario Senac Santo Amaro, Brazil)*; Cecilia Baccino (Universidad de la República, Uruguay)*; Claudia Bambs (Pontificia Universidad Católica de Chile, Chile)*; João Luiz Bastos (Universidade Federal de Santa Catarina, Brazil)*; Gloria Beckles (Centers for Disease Control and Prevention, USA)*; Antonio Bernabe-Ortiz (Universidad Peruana Cayetano Heredia, Perú)*; Carla DO Bernardo (The University of Adelaide, Australia)*; Katia V Bloch (Universidade Federal do Rio de Janeiro (UFRJ), Brazil)*; Juan E Blümel (Universidad de Chile, Chile)*; Jose G Boggia (Universidad de la República, Uruguay)*; Pollyanna K Borges (Universidade Estadual de Ponta Grossa, Brazil)*; Miguel Bravo (MELISA Institute, Chile)*; Gilbert Brenes-Camacho (Universidad de Costa Rica, Costa Rica)*; Horacio A Carbajal (Universidad Nacional de la Plata, Argentina)*; Maria S Castillo Rascon (Universidad Nacional de Misiones, Argentina)*; Blanca H Ceballos (Hospital Dr Ramon Madariaga, Argentina)*; Veronica Colpani (Federal University of Rio Grande do Sul, Brazil)*; Jackie A Cooper (Queen Mary University of London, UK)*; Sandra Cortes (Pontificia Universidad Católica de Chile, Chile)*; Adrian Cortes-Valencia (National Institute of Public Health, Mexico)*; Roberto S Cunha (Federal University of Espírito Santo, Brazil)*; Eleonora d'Orsi (Universidade Federal de Santa Catarina, Brazil)*; William H Dow (University of California, Berkeley, USA)*; Walter G Espeche (Universidad Nacional de la Plata, Argentina)*; Flavio D Fuchs (Universidade Federal do Rio Grande do Sul, Brazil)*; Sandra C Fuchs (Universidade Federal do Rio Grande do Sul, Brazil)*; Suely GA Gimeno (Universidad Federal de São Paulo, Brazil)*; Donaji Gomez-Velasco (Instituto Nacional de Ciencias Médicas y Nutrición, México)*; David A Gonzalez-Chica (The University of Adelaide, Australia)*; Clicerio Gonzalez-Villalpando (Instituto Nacional de Salud Pública, México)*; María-Elena Gonzalez-Villalpando (Centro de Estudios en Diabetes A.C., México)*; Gonzalo Grazioli (Hospital Churruca Visca, Argentina)*; Ricardo O Guerra (Federal University of Rio Grande do Norte, Brazil)*; Laura Gutierrez (Institute for Clinical Effectiveness and Health Policy, Argentina)*; Fernando L Herkenhoff (Federal University of Espírito Santo, Brazil)*; Andrea RVR Horimoto (University of São Paulo, Brazil)*; Andrea Huidobro (Universidad Católica del Maule, Chile)*; Elard Koch (MELISA Institute, Chile)*; Martin Lajous (Harvard T.H. Chan School of Public Health, USA; National Institute of Public Health, Mexico)*; Maria Fernanda Lima-Costa (Oswaldo Cruz Foundation, Brazil)*; Ruy Lopez-Ridaura (National Institute of Public Health, Mexico)*; Alvaro CC Maciel (Federal University of Rio Grande do Norte, Brazil)*; Betty S Manrique-Espinoza (National Institute of Public Health, Mexico)*; Larissa P Marques (Universidade Federal de Santa Catarina, Brazil)*; Jose G Mill (Federal University of Espírito Santo, Brazil)*; Leila B Moreira (Universidade Federal do Rio Grande do Sul, Brazil)*; Oscar M Muñoz (Pontificia Universidad Javeriana, Colombia)*; Lariane M Ono (Universidade Federal do Paraná, Brazil)*; Karen Oppermann (Passo Fundo University, Brazil)*; Karina M Paiva (Universidade Federal de Santa Catarina, Brazil)*; Sergio V Peixoto (Oswaldo Cruz Foundation, Brazil)*; Alexandre C Pereira (University of São Paulo, Brazil)*; Karen G Peres (NDRIS/NDCS Duke-NUS Medical School, Singapore)*; Marco A Peres (NDRIS/NDCS Duke-NUS Medical School, Singapore)*; Paula Ramírez-Palacios (IMSS Epidemiology and Health Services Research Unit, Mexico)*; Cassiano R Rech (Universidade Federal de Santa Catarina, Brazil)*; Berenice Rivera-Paredez (National Autonomous University of Mexico, Mexico)*; Nohora I Rodriguez (Clinica de Marly, Colombia)*; Rosalba Rojas-Martinez (Instituto Nacional de Salud Pública, México)*; Luis Rosero-Bixby (Universidad de Costa Rica, Costa Rica)*; Adolfo Rubinstein (Institute for Clinical Effectiveness and Health Policy, Argentina)*; Alvaro Ruiz-Morales (Pontificia Universidad Javeriana, Colombia)*; Martin R Salazar (Universidad Nacional de la Plata, Argentina)*; Aaron Salinas-Rodriguez (National Institute of Public Health, Mexico)*; Jorge Salmerón (National Autonomous University of Mexico, Mexico)*; Ramon A Sanchez (Universidad Nacional de Misiones, Argentina)*; Nelson AS Silva (Universidade Federal do Rio de Janeiro (UFRJ), Brazil)*; Thiago LN Silva (Universidade federal do Rio de Janeiro (UFRJ), Brazil)*; Liam Smeeth (London School of Hygiene & Tropical Medicine, UK)*; Poli M Spritzer (Federal University of Rio Grande do Sul, Brazil)*; Fiorella Tartaglione (Hospital Churruca Visca, Argentina)*; Jorge Tartaglione (Hospital Churruca Visca, Argentina)*; Rafael Velázquez-Cruz (National Institute of Genomic Medicine (INMEGEN), Mexico)*

## Declaration of Competing Interest

The authors declare no conflict of interests. The funders had no role in study design, data collation and analysis, decision to publish, or preparation of the manuscript. The authors alone are responsible for the views expressed in this paper, which do not necessarily represent the views, decisions, or policies of the institutions with which the authors are affiliated.

## Appendix Supplementary materials

Image, application 1
