# Supplementary material for: Impact of common cardio-metabolic risk factors on fatal and non-fatal cardiovascular disease in Latin America and the Caribbean: an individual-level pooled analysis of 31 cohort studies
Source: Lancet Reg Health Am. 2021 Sep 17;4:100068. doi: 10.1016/j.lana.2021.100068 (PMC8669782; doi:10.1016/j.lana.2021.100068)

Supplementary Material

Impact of common cardio-metabolic risk factors on fatal and non-fatal cardiovascular disease in Latin America and the Caribbean: an individual-level pooled analysis of 31 cohort studies

Expanded methods 3

Data sources 3

Poisson regression 5

Regression dilution bias (RDB) 6

Multiple imputation 7

Interpolation of age-specific relative risks 8

Population attributable fractions 8

References 10

Supplementary Table 1: Ascertainment of cardiovascular events by analysed cohort study 12

Supplementary Table 2: Number (%) of missing observations per risk factor by pooled cohort 13

Supplementary Table 3A: Key characteristics of pooled cohorts at baseline and number of fatal and non-fatal events 14

Supplementary Table 3B: Key characteristics of pooled cohorts at baseline by sex 16

Supplementary Table 4: Number of participants eligible at baseline and events analysed in the complete-case approach by outcome, risk factor and age group 18

Supplementary Table 5: Age-specific relative risk estimates (95% CI), by outcome and risk factor, with and without correction for regresssion dilution bias (RDB), complete-case analysis and multiple imputation 20

Supplementary Figure 1: Comparison of relative risk as per complete-case analysis versus multiple imputation analysis by outcome, risk factor and age group 22

Supplementary Table 6: Relative risk estimates (95% CI) for each cardio-metabolic risk factor on fatal cardiovascular events by sub-region (Central America & Caribbean vs South America) and age group 23

Supplementary Figure 2: Interpolation of age-specific relative risks for each cardio-metabolic risk factor 24

Supplementary Table 7: Age-specific relative risks (95% confidence interval) for fatal cardiovascular diseases estimated through interpolation 25

Supplementary Figure 3: Country charts (alphabetical order) 38

Expanded methods

All analysis codes in R are available as supplementary files with this manuscript. All analyses were conducted with R statistical software (version 3.6.1).

Data sources

We pooled and analysed population-based cohort studies of cardio-metabolic risk factors. These were defined by cohorts where participants were not recruited based on disease (e.g., cohort of stroke survivors) or risk factor history (e.g., cohort of smokers). Data were collated by the Cohorts Consortium of Latin America and the Caribbean (CC-LAC), a LAC network of health researchers and practitioners. The database was collated using multiple data identification sources. First, we accessed publicly available cohort data through each study's website or data repository. Second, we conducted a systematic search in Medline, Embase and SciELO, a LAC-based search engine to identify cohort studies with peer-reviewed publications in regional journals. The search query included country terms (countries in LAC), cohort studies (e.g., cohort stud*), and cardiovascular outcomes (e.g., stroke). Third, enquiries were sent to LAC researchers to identify principal investigators of the selected cohorts. LAC members of the NCD Risk Factor Collaboration (http://ncdrisc.org) also helped identify additional data sources. We invited all eligible cohorts to join the CC-LAC requesting access to anonymised individual-level data.

For the systematic search we used these three sets of terms: i) terms referring to countries in LAC; ii) terms referring to prospective/cohort studies; and iii) terms addressing cardiovascular outcomes. These three sets of terms were combined with the "*and"* Bolean connector.

("Antigua and Barbuda" OR "Argentina" OR "Bahamas" OR "Barbados" OR "Belize" OR "Bermuda" OR "Bolivia" OR "Brazil" OR "Chile" OR "Colombia" OR "Costa Rica" OR "Cuba" OR "Dominica" OR "Dominican Republic" OR "Ecuador" OR "El Salvador" OR "Grenada" OR "Guatemala" OR "Guyana" OR "Haiti" OR "Honduras" OR "Jamaica" OR "Mexico" OR "Nicaragua" OR "Panama" OR "Paraguay" OR "Peru" OR "Puerto Rico" OR "Saint Kitts and Nevis" OR "Saint Vincent and the Grenadines" OR "Saint Lucia" OR "Suriname" OR "Trinidad and Tobago" OR "Uruguay" OR "Venezuela")

("Follow-Up Studies" OR "Longitudinal Studies" OR "Prospective Studies" OR Cohort stud* OR Prospective cohort* OR Prospective stud*)

("Stroke" OR "Cardiovascular Diseases" OR Cardiovascular diseas* OR Cardiovascular event* OR stroke* OR brain-vascular accident* OR CVA OR cerebrovascular accident* OR heart attack* OR myocardial infarction* OR "Myocardial Infarction")

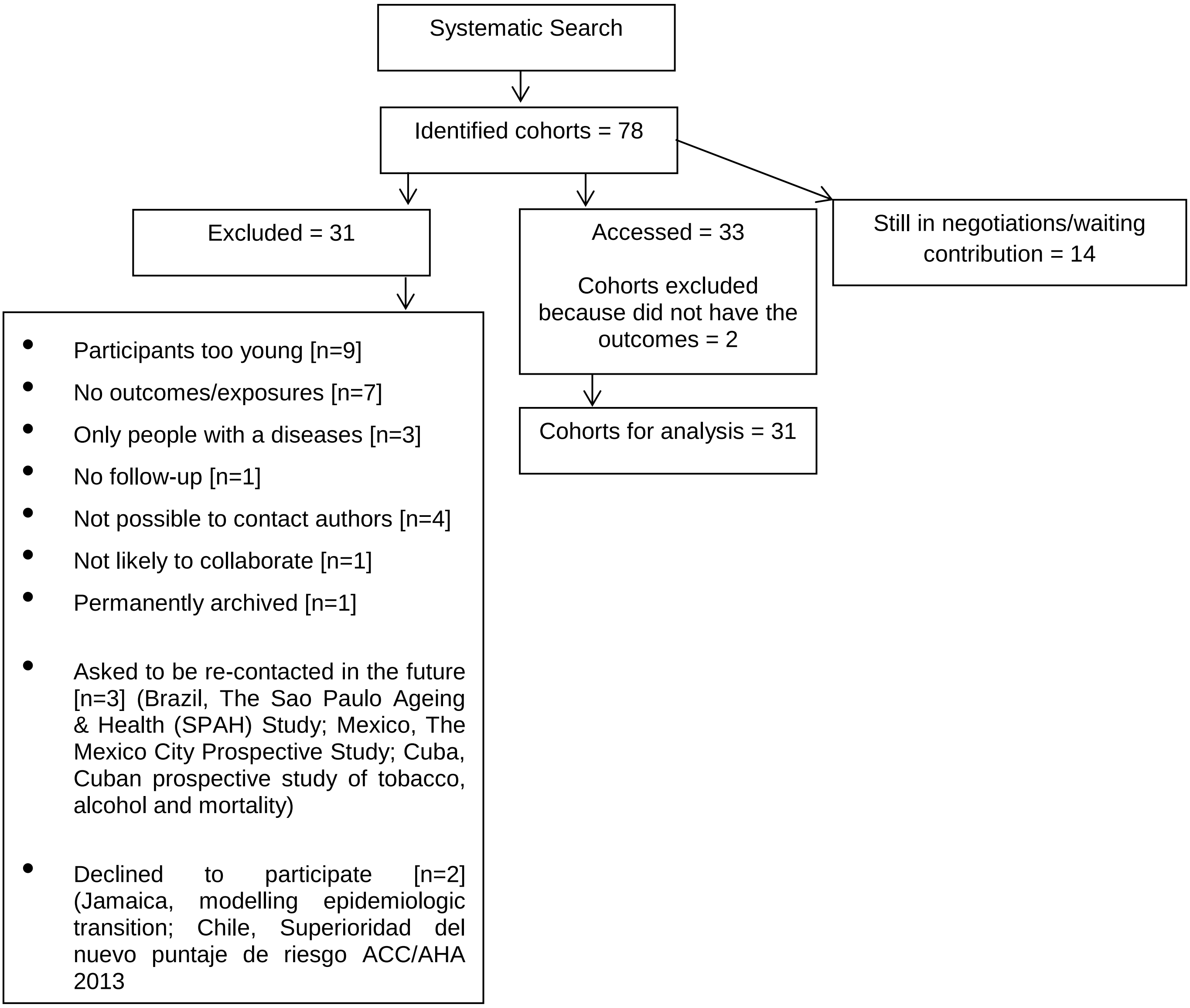

Not likely to collaborate, referred to other research groups with known conflict of interest with our work and would most likely not accept to be involved in the collaboration. *Not possible to contact the authors,* referred to when the original publication did not have contact information (e.g., email address) or the contact information was no longer valid and we could not find other ways to contact them.

Cohort studies were included if they had at least one baseline and one follow-up measurement, and included LAC populations living in LAC. No restrictions were set regarding a minimum sample size at baseline or follow-up duration, sex or age profiles. Anonymised individual record data were received, harmonized and pooled. During the harmonization process, we excluded participants who had history of a cardiovascular event at baseline (stroke or myocardial infarction); though no specific information was available about percutaneous transluminal coronary angioplasty or coronary artery bypass graft. Implausible values of cardio-metabolic risk factors were set to missing based on the following lower and upper limits:[1-4] body mass index (BMI, <10 Kg/m^2 or >80 Kg/m^2), systolic blood pressure (<70

mmHg or >270 mmHg), diastolic blood pressure (<30 mmHg or >150 mmHg), total cholesterol (<1.75 mmol/L or >20.00 mmol/L), HDL-Cholesterol (<0.40 mmol/L or >5.00 mmol/L), and fasting glucose (<2.50 mmol/L or >30.00 mmol/L). BMI was computed based on measured weight and height in all but one cohort;[5] and the self-reported weight and height in that cohort showed little bias based on a validation study.[6] Blood pressure was measured in the resting position and blood biomarkers (i.e., fasting glucose and lipid profiles) were measured in laboratory facilities by each cohort. Non-HDL cholesterol was estimated as total cholesterol minus HDL cholesterol. Regarding the outcome variables (i.e., cardiovascular events), we did not pool cohorts in which this information was based solely on self-reported information (e.g., informed by a relative without further verification with death certificates or clinical records). We did this to secure outcome data of the highest quality.

In this work, we analysed data from 31 cohorts to inform the risk estimates for fatal outcomes, whereas the risk estimates for fatal and non-fatal outcomes were informed by 13 cohorts.

Poisson regression

Having pooled all cohorts into one dataset, we used a Poisson regression model to estimate incidence rate ratios, which were reported as relative risks (RR) along with 95% confidence intervals (95% CI).[7] The regression model included an offset term with the natural logarithm of the follow-up time which allows the estimated incidence rate ratios to approximate hazard ratios from a Cox proportional hazard model.

The RR were adjusted by sex and age (at risk). We did not adjust for other variables because we aimed to compare our RR with those from global cohorts which were sex- and age-adjusted only.[7] In a sensitivity analysis, we adjusted for smoking as a confounder and the RRs were materially unchanged:

	**RR (95% confidence interval)**	
	**Original analysis**	**Additionally adjusted for smoking**
	**Fatal cardiovascular events**	
Body mass index (5 kg/^{2})	1.16 (1.10-1.23)	1.18 (1.11-1.25)
Systolic blood pressure (10 mmHg)	1.23 (1.20-1.25)	1.23 (1.20-1.25)
Fasting glucose (1 mmol/l)	1.11 (1.09-1.13)	1.11 (1.09-1.13)
Total cholesterol (1 mmol/l)	1.07 (1.02-1.11)	1.06 (1.02-1.11)
Non-HDL cholesterol (1 mmol/l)	1.04 (0.99-1.10)	1.03 (0.98-1.09)
	**Non-fatal and fatal cardiovascular events**	

Body mass index (5 kg/^{2})	1.29 (1.18-1.41)	1.29 (1.18-1.41)
Systolic blood pressure (10 mmHg)	1.33 (1.30-1.36)	1.33 (1.30-1.37)
Fasting glucose (1 mmol/l)	1.12 (1.09-1.15)	1.12 (1.09-1.15)
Total cholesterol (1 mmol/l)	1.19 (1.12-1.27)	1.19 (1.12-1.27)
Non-HDL cholesterol (1 mmol/l)	1.28 (1.10-1.50)	1.28 (1.09-1.50)

Results for complete-case analysis.

Regression dilution bias (RDB)

We adjusted our relative risk estimates for short-term variations in cardio-metabolic risk factors by examining the associations of *usual levels* of the risk factor of interest, rather than levels measured at baseline, which would lead to lower RRs due to the larger variations in one-off measurements, known as regression dilution bias.[8]

Following standard methods (e.g., MacMahon) for computing RDB coefficients,[9] we estimated these for the cardio-metabolic risk factors of interest using individual-level data from 10 cohorts that had at least two measurements; that is, we pooled subject-level data of the 10 cohorts and conducted the following methods. We identified the last available measurement of each cardio-metabolic risk factor. We then divided the baseline values of each risk factor in quintiles and estimated the mean of the baseline and last available measurements in each quintile. Finally, we used the following formula to compute the RDB coefficient.

$$\frac{Mean\ at\ the\ top\ quintile\ at\ baseline - mean\ at\ the\ bottom\ quantile\ at\ baseline}{Mean\ at\ the\ top\ quintile\ at\ latest\ available - mean\ at\ the\ bottom\ quantile\ at\ latest\ available}$$

For example, for BMI we computed:

Quintile (baseline)	Baseline (mean, Kg/m^2)	Latest available (mean, Kg/m^2)
1st	20.25	21.19
2nd	23.44	24.15
3rd	25.78	26.22
4th	28.18	28.35
5th	32.94	32.70

$$\frac{32.94 - 20.25}{32.70 - 21.19} = 1.10$$

These procedures were repeated for each cardio-metabolic risk factor yielding the following RDB coefficients:

Risk factor	Regression dilution bias coefficient
Body mass index	1.10
Systolic blood pressure	1.50
Fasting glucose	1.53
Total cholesterol	1.75
Non-HDL cholesterol	1.85

These estimated RDB coefficients were very similar in magnitude to those used by the Asian Pacific Cohort Studies Collaboration: 1.9 for systolic blood pressure, 1.6 for glucose, 1.7 for total cholesterol, 1.9 for HDL-cholesterol and 1.2 for body mass index.[10]

These RDB coefficients were multiplied by both the coefficients of the Poisson regression models (i.e. the relative risks in the logarithmic scale) as well as the corresponding standard errors (also in the logarithmic scale); see an example below for BMI (RDB coefficient: 1.10).

Log (RR)	Lower 95% CI	Upper 95% CI	RDB: Log (RR)	RDB: Lower 95% CI	RDB: Upper 95% CI
0.451895	0.158102	0.745688	0.451895 *1.10 = 0.497084	0.158102 * 1.10 = 0.173912	0.745688 *1.10 = 0.820256

Results presented in the manuscript as RDB-adjusted RR would be (in the log scole): 0.49 (0.17-0.82).

Multiple imputation

Multiple imputation was conducted in R with the *MICE* package, where *MICE* stands for multiple imputation by chained equations.[11] The multiple imputation was applied to the pooled dataset containing all cohorts, as opposed to conducting imputations within each cohort separately, allowing us to borrow information across cohorts. The number of missing observations by variables and cohort is presented in Supplementary Table 2.

Before running the multiple imputation models, missing observations in sex (n=12) were dropped. The prediction matrix was modified as follows: variables with complete data (i.e. without missing observations) should not be predicted; these included: sex; age at baseline and age at event along

with the corresponding age categories; time at event; and outcome variables (fatal and non-fatal events).

We also specified the imputation method for each variable. Continuous variables were imputed with predictive mean matching (*pmm* option); whereas categorical variables were imputed with the method proposed by Jolani and colleagues for individual-participant meta-analysis.[12] These methods were specified in a matrix and included in the multiple imputation syntax.

We restricted the sample size to those aged 20 years and above, thus the pooled database for multiple imputation included 175,791 observations. We specified 50 iterations (*maxit* option) and 2 multiple imputations (*m* option). We ran 25 rounds of multiple imputations, which were then pooled (*miceadds* package in R). Therefore, we had 50 multiple imputation datasets (2 x 25).

Interpolation of age-specific relative risks

To maximize statistical power and number of outcome events, we computed the age-specific relative risks in 10-year age bands (35-44, 45-54…85+). However, to estimate population attributable fractions and absolute number of attributable deaths with higher granularity, we interpolated relative risks of fatal CVD in 5-year age groups (35-39, 40-44….85+) using a log-linaer model.[7] This process was conducted with the relative risks adjusted by RDB based on the multiple imputation analysis. The fit of these models is shown on Supplementary Figure 2 and the estimated relative risks are presented on Supplementary Table 7.

Population attributable fractions

We followed a comparative risk assessment approach to estimate the population attributable fractions for each cardio-metabolic risk factor on fatal cardiovascular outcomes.[13] The inputs for this process were:

i) Age-specific relative risk as per the interpolation analysis explained above.
ii) Age-sex-specific mean of the selected cardio-metabolic risk factors for each country in Latin America and the Caribbean, which were extracted from the NCD-RisC.[2, 3, 14]
iii) Number of cardiovascular deaths, which were extracted from the Global Burden of Disease (IHME) 2019 results.[15]

We assumed the following theoretical minimum-risk exposure distribution:[7]

Cardio-metabolic risk factor	Theoretical minimum-risk exposure distribution
Systolic blood pressure (mmHg)	110-115
Body mass index (kg/m^2)	21-23
Total cholesterol (mmol/L)	3.8-4.0
Non-HDL total cholesterol (mmol/L)	1.8-2.2

The estimation of the population attributable fractions was ran 1,000 times. The mean of this distribution was extracted as the main results (i.e., population attributable fractions shown in the main text), while the 0.025 and 0.975 percentiles were extracted to compute the 95% credible interval.

To compute the absolute number of attributable deaths to each cardio-metabolic risk factor, we multiplied the population attributable fractions by the number of cardiovascular deaths in each country in Latin America and the Caribbean. Results are presented as crude attributable death rates per 100,000. The number of age-specific cardiovascular disease deaths and population size by country and sex is shown in Supplementary Table 8 (source: GBD Study 2017).

References

1. NCD Risk Factor Collaboration (NCD-RisC). Worldwide trends in diabetes since 1980: a pooled analysis of 751 population-based studies with 4.4 million participants. *Lancet (London, England)* 2016; **387**(10027): 1513-30.

2. NCD Risk Factor Collaboration (NCD-RisC). Worldwide trends in body-mass index, underweight, overweight, and obesity from 1975 to 2016: a pooled analysis of 2416 population-based measurement studies in 128.9 million children, adolescents, and adults. *Lancet (London, England)* 2017; **390**(10113): 2627-42.

3. NCD Risk Factor Collaboration (NCD-RisC). Worldwide trends in blood pressure from 1975 to 2015: a pooled analysis of 1479 population-based measurement studies with 19.1 million participants. *Lancet (London, England)* 2017; **389**(10064): 37-55.

4. Farzadfar F, Finucane MM, Danaei G, et al. National, regional, and global trends in serum total cholesterol since 1980: systematic analysis of health examination surveys and epidemiological studies with 321 country-years and 3.0 million participants. *Lancet (London, England)* 2011; **377**(9765): 578-86.

5. Lajous M, Ortiz-Panozo E, Monge A, et al. Cohort Profile: The Mexican Teachers' Cohort (MTC). *International journal of epidemiology* 2017; **46**(2): e10.

6. Ortiz-Panozo E, Yunes-Diaz E, Lajous M, Romieu I, Monge A, Lopez-Ridaura R. Validity of self-reported anthropometry in adult Mexican women. *Salud publica de Mexico* 2017; **59**(3): 266-75.

7. Singh GM, Danaei G, Farzadfar F, et al. The age-specific quantitative effects of metabolic risk factors on cardiovascular diseases and diabetes: a pooled analysis. *PloS one* 2013; **8**(7): e65174.

8. Woodward M. Epidemiology: Study Design and Data Analysis, Third Edition: Taylor & Francis; 2013.

9. Frost C, Thompson SG. Correcting for Regression Dilution Bias: Comparison of Methods for a Single Predictor Variable. *Journal of the Royal Statistical Society Series A (Statistics in Society)* 2000; **163**(2): 173-89.

10. Woodward M, Barzi F, Martiniuk A, et al. Cohort profile: the Asia Pacific Cohort Studies Collaboration. *International journal of epidemiology* 2006; **35**(6): 1412-6.

11. van Buuren S, Groothuis-Oudshoorn K. mice: Multivariate Imputation by Chained Equations in R. *2011* 2011; **45**(3): 67.

12. Jolani S, Debray TP, Koffijberg H, van Buuren S, Moons KG. Imputation of systematically missing predictors in an individual participant data meta-analysis: a generalized approach using MICE. *Statistics in medicine* 2015; **34**(11): 1841-63.

13. Ezzati M, Lopez AD, Rodgers A, Vander Hoorn S, Murray CJ. Selected major risk factors and global and regional burden of disease. *Lancet (London, England)* 2002; **360**(9343): 1347-60.

14. NCD Risk Factor Collaboration (NCD-RisC). Repositioning of the global epicentre of non-optimal cholesterol. *Nature* 2020; **582**(7810): 73-7.

15. Global Burden of Disease Collaborative Network. Global Burden of Disease Study 2019 (GBD 2019) Results. Seattle, United States: Institute for Health Metrics and Evaluation (IHME), 2020. Available from http://ghdx.healthdata.org/gbd-results-tool.

Supplementary Table 1: Ascertainment of cardiovascular events by analysed cohort study

Cohort	Fatal events	Non-fatal events
CRELES (Costa Rica)	Linked to national vital registration (death certificates)	Not analysed
CRELES Retirement (Costa Rica)	Not analysed	Not analysed
CRONICAS (Peru)	Not analysed	Not analysed
PERU MIGRANT Study (Peru)	Linked to national vital registration (death certificates)	Not analysed
Mexican Health & Aging Study (Mexico)	Not analysed	Not analysed
Puerto Rico Heart Health Program (Puerto Rico)	Adjudication	Adjudication
Rauch City (Argentina)	Adjudication	Adjudication
St James Survey (Trinidad & Tobago)	Adjudication	Adjudication
Puerto Rican Elderly: Health Conditions (Puerto Rico)	Adjudication	Adjudication
10/66 Dementia Study (Multi-country)	Adjudication	Not analysed
Validación de los modelos de predicción de Framingham y PROCAM como estimadores del riesgo cardiovascular en una población colombiana (Colombia)	Adjudication	Clinical records
The Bambuí Cohort Study of Ageing (Brazil)	Linked to national vital registration (death certificates)	Not analysed
Eventos cardiovasculares en una población cerrada (Argentina)	Linked to national vital registration (death certificates)	Adjudication
Impact of cognitive deficit on survival among elderly residents in the community (Brazil)	Not analysed	Not analysed
Anthropometric Indexes Predicting Cardiometabolic Risk. Prospective Cohort Study in a Population of Employees of Public Hospitals (Argentina)	Linked to national vital registration (death certificates)	Adjudication
Estudio Barros Luco (Chile)	Linked to national vital registration (death certificates)	Clinical records
GEnotipo, Fenotipo y Ambiente de la HiperTensión Arterial en UruguaY (GEFA-HT-UY, Uruguay)	Clinical records	Clinical records
Japanese-Brazilian Diabetes Study Group (Brazil)	Linked to national vital registration (death certificates)	Not analysed
Centro de Excelencia en Salud Cardiovascular para América del Sur (CESCAS) (Mutli-country)	Linked to national vital registration (death certificates) or adjudication	Adjudication
Mexican Teacher's Cohort (Mexico)	Linked to national vital registration (death certificates)	Not analysed
The Mexico City Diabetes Study (Mexico)	Linked to national vital registration (death certificates) or adjudication	Not analysed
Porto Alegre Cohort (Brazil)	Linked to national vital registration (death certificates) or adjudication	Adjudication
The Passo Fundo Cohort Study (Brazil)	Clinical records	Not analysed
Epifloripa Cohort Study of Ageing (Brazil)	Linked to national vital registration (death certificates))	Not analysed
WHO Study on Global Ageing and Adult Health – Mexico (Mexico)	Linked to national vital registration (death certificates)	Not analysed
St Francisco Project (Chile)	Linked to national vital registration (death certificates)	Clinical records
Baependi Heart Study (Brazil)	Not analysed	Not analysed
Maule Cohort of chronic diseases (MAUCO, Chile)	Linked to national vital registration (death certificates)	Clincial records
Epifloripa Cohort Study of Adults (Brazil)	Linked to national vital registration (death certificates)	Not analysed
Metabolic Syndrome Cohort (Mexico)	Clinical records	Clinical records
MONICA-VITÓRIA (Brazil)	Not analysed	Not analysed
HAIG-Long (Brazil)	Linked to national vital registration (death certificates)	Not analysed
Health Workers Cohort Study (HWCS, Mexico)	Linked to national vital registration (death certificates)	Not analysed

Adjudication refers to the process in which researchers or clinicians revise documentation and agree on the outcome; for example, researchers could revise the clinical chart of a hospitalization and agree that the most likely outcome was myocardial infarction.

Supplementary Table 2: Number (%) of missing observations per risk factor by pooled cohort

Study	Sample size	Sex		BMI		Total cholesterol		HDL-cholesterol		Fasting glucose		Systolic blood pressure	
	N	N	%	N	%	N	%	N	%	N	%	N	%
Eventos cardiovasculares en una población cerrada	2358	0	0.0	8	0.3	74	3.1	116	4.9	73	3.1	6	0.3
Rauch City	1105	0	0.0	11	1.0	202	18.3	1105	100.0	202	18.3	0	0.0
Anthropometric Indexes Predicting Cardiometabolic Risk. Prospective Cohort Study in a…	401	0	0.0	0	0.0	0	0.0	0	0.0	30	7.5	1	0.2
CESCAS – Argentina	3836	0	0.0	9	0.2	60	1.6	62	1.6	61	1.6	5	0.1
Porto Alegre Cohort	855	0	0.0	0	0.0	855	100.0	855	100.0	855	100.0	0	0.0
Hipertensão Arterial na Ilha do Governador - HAIG-Long (Hypertension in Ilha do Governador)	1224	0	0.0	3	0.2	1224	100.0	1224	100.0	1224	100.0	0	0.0
JBDSG	1333	1	0.1	14	1.1	1	0.1	3	0.2	1	0.1	14	1.1
The Bambuí Cohort Study of Ageing	1364	0	0.0	29	2.1	1	0.1	1	0.1	2	0.1	0	0.0
The Passo Fundo Cohort Study	342	0	0.0	0	0.0	2	0.6	2	0.6	2	0.6	14	4.1
Epifloripa Cohort Study of Adults	1241	0	0.0	12	1.0	1241	100.0	1241	100.0	1241	100.0	30	2.4
Epifloripa Cohort Study of Ageing	1257	0	0.0	31	2.5	702	55.8	702	55.8	704	56.0	37	2.9
Estudio Barros Luco	1198	0	0.0	0	0.0	0	0.0	4	0.3	0	0.0	0	0.0
St Francisco Project	866	0	0.0	866	100.0	636	73.4	638	73.7	463	53.5	866	100.0
CESCAS – Chile	1881	0	0.0	0	0.0	67	3.6	67	3.6	67	3.6	6	0.3
Maule Cohort Study (MAUCO)	3182	0	0.0	16	0.5	18	0.6	20	0.6	18	0.6	5	0.2
Validación de los modelos de predicción de Framingham y PROCAM como…	847	0	0.0	22	2.6	0	0.0	0	0.0	847	100.0	0	0.0
CRELES	2542	0	0.0	251	9.9	149	5.9	154	6.1	153	6.0	76	3.0
10/66 – Cuba	2307	0	0.0	2307	100.0	460	19.9	879	38.1	475	20.6	6	0.3
10/66 – Dominican Republic	1509	1	0.1	1509	100.0	377	25.0	1509	100.0	377	25.0	13	0.9
The Mexico City Diabetes Study	1914	0	0.0	1	0.1	0	0.0	15	0.8	0	0.0	1	0.1
10/66 – Mexico	1555	0	0.0	1555	100.0	209	13.4	216	13.9	211	13.6	5	0.3
Health Workers Cohort Study (HWCS)	2057	0	0.0	12	0.6	121	5.9	37	1.8	19	0.9	260	12.6
The metabolic syndrome cohort	6119	0	0.0	0	0.0	0	0.0	4	0.1	2	0.0	0	0.0
MTC	115312	0	0.0	10785	9.4	109561	95.0	110583	95.9	109578	95.0	109336	94.8
WHO Study on Global Ageing and Adult Health (SAGE)	2624	10	0.4	300	11.4	2624	100.0	2624	100.0	2624	100.0	220	8.4
10/66 – Peru	1326	0	0.0	1326	100.0	769	58.0	1326	100.0	768	57.9	7	0.5
PERU MIGRANT Study	977	0	0.0	1	0.1	1	0.1	1	0.1	1	0.1	1	0.1
PRHHP	9699	0	0.0	20	0.2	60	0.6	9699	100.0	46	0.5	14	0.1
10/66 – Puerto Rico	1385	0	0.0	1385	100.0	200	14.4	200	14.4	209	15.1	202	14.6
St James Survey	623	0	0.0	0	0.0	5	0.8	31	5.0	2	0.3	1	0.2
CESCAS – Uruguay	1496	0	0.0	5	0.3	32	2.1	31	2.1	31	2.1	1	0.1
GEFA-HT-UY	202	0	0.0	2	1.0	2	1.0	2	1.0	2	1.0	0	0.0
10/66 – Venezuela	1300	0	0.0	1300	100.0	393	30.2	1300	100.0	393	30.2	247	19.0

No missing observations in age at baseline. Total sample size adds up to 176,237 individuals. This number is larger than the analysed sample size (reported in the main paper, n=168,287) beause the latter number excluded people younger than 20 years (n=434), observations with missing sex (n=12), and two exclude cohorts (n=7,504). BMI: body mass index (kg/m^2).

Supplementary Table 3A: Key characteristics of pooled cohorts at baseline and number of fatal and non-fatal events

Study	Eligible Sample	Women	Men	Age	BMI	SBP	Glucose	Total cholesterol	Non-HDL cholesterol	Time	Outcome: Non Fatal	Outcome: Fatal
Eventos cardiovasculares en una población cerrada	2357	0	2357	40 (8.86)	28.52 (4.24)	133 (12.69)	4.85 (1.3)	5.26 (1.14)	4.1 (1.13)	8.77 (1.08)	34	20
Rauch City	1021	665	356	47 (14.74)	26.11 (4.5)	137 (20.33)	4.45 (1.09)	4.88 (0.73)	NM	13.81 (1.89)	61	23
Anthropometric Indexes Predicting Cardiometabolic Risk. Prospective Cohort Study in a Population of Employees of Public Hospitals	401	304	97	4 (8.81)	26.9 (5.05)	119 (15.82)	4.95 (0.66)	5.02 (0.98)	3.81 (1.02)	8.79 (1.81)	9	4
CESCAS – Argentina	3836	2333	1503	54 (10.5)	29.28 (5.85)	130 (20.22)	5.48 (1.8)	5.22 (1.08)	4.03 (1.03)	4.5 (1.06)	23	16
Porto Alegre Cohort	812	436	376	43 (15.83)	25.19 (4.59)	127 (22.55)	NM	NM	NM	5.63 (1.46)	11	8
Hipertensão Arterial na Ilha do Governador - HAIG-Long (Hypertension in Ilha do Governador)	1224	688	536	43 (15.41)	25.05 (4.45)	132 (21.65)	NM	NM	NM	17.1 (3.35)	NI	28
JBDSG	1332	709	623	55 (11.89)	24.46 (3.69)	127 (21.11)	6.16 (1.57)	5.49 (1.09)	4.27 (0.99)	8.15 (3.3)	NI	143
The Bambuí Cohort Study of Ageing	1364	830	534	69 (7.21)	25.13 (4.85)	138 (22.5)	6.01 (2.18)	6.05 (1.27)	4.77 (1.29)	10.83 (4.74)	NI	66
The Passo Fundo Cohort Study	342	342	0	48 (5.57)	27.51 (5.57)	126 (21.64)	4.67 (1.78)	5.17 (1.05)	3.81 (1.06)	9.43 (1.19)	NI	7
Epifloripa Cohort Study of Adults	1241	703	538	39 (11.4)	25.73 (4.74)	133 (19.02)	NM	NM	NM	4.3 (1.22)	NI	1
Epifloripa Cohort Study of Ageing	1257	818	439	711 (8.08)	27.9 (5.05)	143 (21.59)	5.79 (1.72)	4.87 (1.04)	3.57 (1.02)	6.43 (2.35)	NI	27
Estudio Barros Luco	1198	1198	0	49 (5.47)	25.93 (4)	120 (17.76)	4.91 (1.14)	5.72 (1.14)	4.36 (1.14)	24.93 (4.56)	53	32
St Francisco Project	774	445	329	42 (15.1)	NM	NM	5.48 (1.34)	5.33 (1.16)	4.24 (1.15)	8.14 (0.91)	NI	11
CESCAS – Chile	1881	996	885	55 (10.96)	28.97 (4.75)	129 (19.67)	5.73 (2.02)	5.26 (1.1)	4.09 (1.06)	5.06 (0.85)	25	2
Maule Cohort Study (MAUCO)	3182	2402	780	56 (9.46)	29.77 (4.98)	132 (20.11)	5.73 (1.97)	5.03 (1.03)	3.82 (0.99)	2.32 (0.47)	7	10
Validación de los modelos de predicción de Framingham y PROCAM como estimadores del riesgo cardiovascular en una población colombiana	847	548	299	54 (9.01)	27.22 (3.74)	142 (20.6)	NM	6.09 (1.26)	4.7 (1.27)	9.51 (1.6)	29	5
CRELES	2542	1384	1158	76 (10.33)	26.14 (5.08)	146 (24.32)	6.02 (2.26)	5.55 (1.27)	4.38 (1.22)	6.4 (2.57)	NI	153
10/66 – Cuba	2307	1528	779	75 (7.06)	NM	146 (25.72)	5.62 (2.06)	5.33 (1.24)	4.19 (1.27)	4.16 (1.29)	NI	123
10/66 – Dominican Republic	1508	1011	497	75 (7.53)	NM	137 (20.34)	5.22 (1.92)	5.09 (1.43)	NM	4.39 (1.41)	NI	110
The Mexico City Diabetes Study	1914	1122	792	47 (8.28)	28.06 (4.35)	118 (17.53)	5.49 (2.79)	4.95 (1.12)	4.11 (1.09)	12.96 (5.4)	NI	72
10/66 – Mexico	1555	1002	553	74 (6.66)	NM	131 (22.12)	5.83 (2.67)	4.76 (1.05)	3.47 (0.97)	2.9 (0.55)	NI	16
Health Workers Cohort Study (HWCS)	1844	1364	480	50 (14.16)	27 (4.6)	116 (12.67)	5.59 (2)	5.99 (3.21)	4.99 (3.22)	9.51 (3.35)	NI	46
MTC	115312	115312	0	43 (7.53)	27.32 (4.67)	125 (18.33)	5.47 (1.81)	5.36 (1.05)	4.07 (1.01)	9.1 (1.56)	NI	124
WHO Study on Global Ageing and Adult Health (SAGE)	2614	1622	992	64 (14.4)	28.37 (5.39)	144 (25.01)	NM	NM	NM	8.61 (2)	NI	52
10/66 – Peru	1326	814	512	75 (7.51)	NM	121 (13.53)	5.63 (1.45)	5.26 (0.8)	NM	3.09 (0.8)	NI	21
PERU MIGRANT Study	977	513	464	48 (11.89)	26.53 (4.61)	122 (18.48)	4.94 (1.39)	4.78 (1.06)	3.64 (1.07)	10.24 (1.31)	NI	6

PRHHP	9699	0	9699	54 (6.66)	25.08 (3.99)	132 (22.81)	5.34 (1.7)	5.22 (1.07)	NM	11.03 (2.46)	287	539
St James Survey	623	0	623	52 (9.61)	23.92 (3.85)	135 (22.63)	4.92 (1.69)	5.72 (1.17)	4.8 (1.18)	8.42 (1.69)	10	15
CESCAS – Uruguay	1496	882	614	55 (11.24)	28.95 (6.05)	131 (20.47)	5.39 (1.59)	5.45 (1.13)	4.18 (1.1)	4.73 (0.99)	27	3
GEFA-HT-UY	201	128	73	57 (16.33)	29.56 (5.98)	129 (21.53)	5.68 (1.6)	5.5 (1.24)	4.18 (1.19)	2.98 (0.83)	1	0
10/66 – Venezuela	1300	830	470	72 (6.81)	NM	137 (24.75)	5.5. (2.58)	5.57 (1.07)	NM	4.15 (0.93)	0	27

Results for outcomes, gender and total sample size are shown in absolute numbers. Results for age (years), BMI (km/m^2), SBP (mmHg), glucose (mmol/L), total cholesterol (mmol/L), Non-HDL cholesterol (mmol/L) and time (follow-up time in years) are shown as mean with standard deviation. BMI: body mass index; CVDs: cardiovascular diseases; SBP: systolic blood pressure; NM: not measured; NI: cohorts not included in the fatal plus non-fatal analysis.

Supplementary Table 3B: Key characteristics of pooled cohorts at baseline by sex

Study	Age (mean (SD))		BMI (mean (SD))		SBP (mean (SD))		Glucose (mean (SD))		Total cholesterol (mean (SD))		Non-HDL cholesterol (mean (SD))	
	Women	Men	Women	Men	Women	Men	Women	Men	Women	Men	Women	Men
Eventos cardiovasculares en una población cerrada	NM	40 (8.86)	NM	28.52(4.24)	NM	133 (12.69)	NM	4.85(1.3)	NM	5.26(1.14)	NM	4.1(1.13)
Rauch City	47 (14.99)	48 (14.21)	25.76(4.76)	26.77(3.89)	135 (20.27)	142 (19.51)	4.33(0.94)	4.66(1.28)	4.76(0.63)	5.1(0.85)	NM	NM
Anthropometric Indexes Predicting Cardiometabolic Risk. Prospective Cohort Study in a Population of Employees of Public Hospitals	44 (8.34)	43 (10.13)	26.84(5.43)	27.07(3.65)	118 (15.99)	124 (14.28)	4.89(0.65)	5.16(0.65)	4.96(0.94)	5.19(1.1)	3.71(0.96)	4.13(1.13)
CESCAS – Argentina	54 (10.46)	55 (10.56)	29.49(6.43)	28.94(4.8)	128 (20.86)	132 (18.98)	5.41(1.8)	5.6(1.79)	5.23(1.08)	5.21(1.08)	3.97(1.03)	4.11(1.02)
Porto Alegre Cohort	44 (16.29)	42 (15.2)	25.27(5.03)	25.09(4.02)	124 (25.01)	129 (19.03)	NM	NM	NM	NM	NM	NM
Hipertensão Arterial na Ilha do Governador - HAIG-Long (Hypertension in Ilha do Governador)	43 (15.41)	43 (15.42)	25.28(4.71)	24.77(4.09)	130 (23.83)	133 (18.33)	NM	NM	NM	NM	NM	NM
JBDSG	55 (11.5)	56 (12.3)	24.34(3.7)	24.59(3.67)	126 (21.42)	128 (20.73)	6.12(1.5)	6.21(1.65)	5.52(1.1)	5.45(1.09)	4.29(0.99)	4.25(0.99)
The Bambuí Cohort Study of Ageing	69 (7.27)	69 (7.1)	25.85(5.11)	24.04(4.17)	137 (22.23)	138 (22.93)	6.08(2.39)	5.92(1.82)	6.27(1.27)	5.7(1.19)	4.96(1.32)	4.48(1.18)
The Passo Fundo Cohort Study	48 (5.57)	NM	27.51(5.57)	NM	125 (21.64)	NM	4.67(1.78)	NM	5.17(1.05)	NM	3.81(1.06)	NM
Epifloripa Cohort Study of Adults	39 (11.34)	38 (11.48)	25.51(5.07)	26.02(4.27)	127 (18.05)	139 (18.03)	NM	NM	NM	NM	NM	NM
Epifloripa Cohort Study of Ageing	71 (8.18)	70 (7.89)	28.46(5.33)	26.87(4.29)	141 (21.28)	146 (21.84)	5.76(1.83)	5.83(1.5)	5.02(1.01)	4.58(1.05)	3.65(1.01)	3.41(1.02)
Estudio Barros Luco	49 (5.47)	NM	25.93(4)	NM	120 (17.76)	NM	4.91(1.14)	NM	5.72(1.14)	NM	4.36(1.14)	NM
St Francisco Project	42 (15.3)	41 (14.84)	NM	NM	NM	NM	5.39(1.05)	5.64(1.7)	5.44(1.21)	5.13(1.02)	4.34(1.21)	4.07(1.04)
CESCAS – Chile	55 (11.04)	54 (10.88)	29.35(5.28)	28.54(4.04)	126 (20.1)	132 (18.69)	5.61(2.04)	5.87(2)	5.33(1.12)	5.18(1.07)	4.08(1.09)	4.1(1.03)
Maule Cohort Study (MAUCO)	56 (9.34)	57 (9.77)	29.98(5.14)	29.1(4.36)	130 (19.82)	138 (19.98)	5.7(1.99)	5.85(1.91)	5.07(1.03)	4.9(1.02)	3.84(1)	3.78(0.97)
Validación de los modelos de predicción de Framingham y PROCAM como estimadores del riesgo cardiovascular en una población colombiana	53 (8.99)	56 (8.78)	27.42(4.08)	26.84(2.99)	141 (20.72)	144 (20.28)	NM	NM	6.1(1.26)	6.06(1.27)	4.69(1.26)	4.71(1.28)
CRELES	76 (10.28)	76 (10.4)	26.79(5.74)	25.37(4.04)	147 (24.79)	145 (23.71)	6.17(2.25)	5.84(2.27)	5.76(1.29)	5.29(1.2)	4.52(1.25)	4.2(1.15)
10/66 – Cuba	75 (7.27)	75 (6.61)	NM	NM	146 (25.98)	145 (25.2)	5.67(2.15)	5.51(1.86)	5.49(1.24)	5(1.16)	4.33(1.26)	3.91(1.24)
10/66 – Dominican Republic	76 (7.78)	75 (6.99)	NM	NM	136 (20.78)	137 (19.42)	5.18(1.71)	5.3(2.32)	5.24(1.43)	4.74(1.36)	NM	NM
The Mexico City Diabetes Study	47 (8.23)	47 (8.35)	28.73(4.67)	27.12(3.64)	116 (17.94)	120 (16.66)	5.47(2.77)	5.51(2.82)	4.94(1.14)	4.96(1.09)	4.04(1.09)	4.2(1.08)
10/66 – Mexico	74 (6.82)	75 (6.32)	NM	NM	132 (22.73)	129 (20.91)	5.93(2.72)	5.64(2.55)	4.93(1.05)	4.44(0.98)	3.58(0.99)	3.27(0.9)
Health Workers Cohort Study (HWCS)	50 (14.07)	49 (14.4)	26.99(4.79)	27.01(4.01)	115 (12.31)	121 (12.74)	5.5(1.92)	5.86(2.21)	5.72(3.04)	6.74(3.52)	4.69(3.06)	5.84(3.5)
MTC	43 (7.53)	NM	27.32(4.67)	NM	125 (18.33)	NM	5.47(1.81)	NM	5.36(1.05)	NM	4.07(1.01)	NM
WHO Study on Global Ageing and Adult Health (SAGE)	63 (14.69)	64 (13.88)	29.03(5.6)	27.27(4.83)	144 (25.46)	145 (24.22)	NM	NM	NM	NM	NM	NM

10/66 – Peru	75 (7.36)	76 (7.7)	NM	NM	122 (13.94)	121 (12.85)	5.6(1.6)	5.69(1.09)	5.37(0.82)	5.05(0.72)	NM	NM
PERU MIGRANT Study	48 (11.88)	48 (11.91)	27.45(5.17)	25.51(3.63)	118 (18.91)	125 (17.29)	4.95(1.4)	4.93(1.37)	4.81(1.09)	4.74(1.03)	3.64(1.09)	3.63(1.04)
PRHHP	NM	54 (6.66)	NM	25.08(3.99)	NM	132 (22.81)	NM	5.34(1.7)	NM	5.22(1.07)	NM	NM
St James Survey	NM	52 (9.61)	NM	23.92(3.85)	NM	135 (22.63)	NM	4.92(1.69)	NM	5.72(1.17)	NM	4.8(1.18)
CESCAS – Uruguay	56 (11.17)	55 (11.33)	29.74(6.4)	27.83(5.32)	129 (20.63)	133 (19.99)	5.28(1.56)	5.54(1.63)	5.51(1.13)	5.35(1.14)	4.17(1.1)	4.18(1.1)
GEFA-HT-UY	58(16.59)	57 (15.96)	29.71(6.6)	29.28(4.72)	129 (22.74)	128 (19.38)	5.7(1.89)	5.64(0.87)	5.61(1.22)	5.3(1.24)	4.2(1.19)	4.14(1.2)
VEN_2004_10/66	73 (7.24)	72 (5.94)	NaN(NA)	NaN(NA)	137 (24.04)	138 (26.03)	5.43(2.51)	5.62(2.71)	5.67(1.09)	5.4(1.02)	NaN(NA)	NaN(NA)

Results for age (years), BMI (km/m^2), SBP (mmHg), glucose (mmol/L), total cholesterol (mmol/L) and Non-HDL cholesterol (mmol/L) are shown as mean with standard deviation. BMI: body mass index; SBP: systolic blood pressure; NM: not measured.

Supplementary Table 4: Number of participants eligible at baseline and events analysed in the complete-case approach by outcome, risk factor and age group

Age group	Events	Eligible at baseline
Outcome: Fatal (31 cohorts analysed)		
Body mass index (5 kg/m2)		
35-44	26	23590
45-54	158	52654
55-64	368	47019
65-74	430	15666
75-84	279	5822
85+	99	1987
Systolic blood pressure (10 mmHg)		
35-44	17	3966
45-54	109	10618
55-64	311	14820
65-74	470	14718
75-84	391	8877
85+	216	3480
Fasting glucose (1 mmol/L)		
35-44	17	3143
45-54	107	9297
55-64	304	13621
65-74	435	12422
75-84	324	6842
85+	160	2447
Total cholesterol (1 mmol/L)		
35-44	17	3117
45-54	108	9378
55-64	304	13935
65-74	436	12652
75-84	323	6939
85+	159	2447
Non-HDL cholesterol (1 mmol/L)		
35-44	15	2938
45-54	55	8007
55-64	102	8348
65-74	172	7640
75-84	205	4995
85+	115	1870
Outcome: Fatal plus non-fatal (13 cohorts analysed)		
Body mass index (5 kg/m2)		
35-44	23	2442
45-54	174	5056
55-64	482	9973

65-74	429	7638
75-84	134	1990
Systolic blood pressure (10 mmHg)		
35-44	23	2442
45-54	174	5067
55-64	481	9980
65-74	433	7663
75-84	134	1999
Fasting glucose (1 mmol/L)		
35-44	22	2178
45-54	165	4637
55-64	452	9399
65-74	410	7234
75-84	120	1804
Total cholesterol (1 mmol/L)		
35-44	22	2200
45-54	169	4761
55-64	469	9750
65-74	424	7494
75-84	121	1906
Non-HDL cholesterol (1 mmol/L)		
35-44	14	2065
45-54	51	4018
55-64	102	4520
65-74	100	3735
75-84	43	1246

Supplementary Table 5: Age-specific relative risk estimates (95% CI), by outcome and risk factor, with and without correction for regression dilution bias (RDB), complete-case analysis and multiple imputation

Outcome	Risk factor	Age group	Complete-case: RR (95% CI)	Complete-case: RDB-RR (95% CI)	Multiple imputation: RDB-RR (95% CI)
Fatal	Body Mass Index (5 kg/m^2)	35-44	1.67 (1.25-2.23)	1.76 (1.28-2.42)	1.75 (1.27-2.41)
Fatal	Body Mass Index (5 kg/m^2)	45-54	1.21 (1.2-1.21)	1.23 (1.23-1.23)	1.22 (1.14-1.31)
Fatal	Body Mass Index (5 kg/m^2)	55-64	1.22 (1.09-1.37)	1.25 (1.1-1.42)	1.24 (1.1-1.41)
Fatal	Body Mass Index (5 kg/m^2)	65-74	1.17 (1.06-1.3)	1.19 (1.06-1.34)	1.16 (1.04-1.3)
Fatal	Body Mass Index (5 kg/m^2)	75-84	1 (0.88-1.15)	1 (0.86-1.16)	1.03 (0.91-1.16)
Fatal	Body Mass Index (5 kg/m^2)	85+	0.94 (0.75-1.17)	0.93 (0.73-1.19)	1 (0.84-1.18)
Fatal	Fasting Glucose (1 mmol/L)	35-44	1.11 (0.81-1.51)	1.17 (0.73-1.88)	1.3 (0.91-1.86)
Fatal	Fasting Glucose (1 mmol/L)	45-54	1.14 (1.07-1.21)	1.22 (1.11-1.35)	1.23 (1.21-1.26)
Fatal	Fasting Glucose (1 mmol/L)	55-64	1.14 (1.1-1.19)	1.23 (1.16-1.3)	1.23 (1.17-1.3)
Fatal	Fasting Glucose (1 mmol/L)	65-74	1.11 (1.08-1.14)	1.18 (1.13-1.22)	1.18 (1.14-1.23)
Fatal	Fasting Glucose (1 mmol/L)	75-84	1.08 (1.03-1.13)	1.13 (1.05-1.21)	1.12 (1.05-1.19)
Fatal	Fasting Glucose (1 mmol/L)	85+	0.95 (0.86-1.04)	0.92 (0.79-1.07)	0.98 (0.86-1.11)
Fatal	non-HDL-Cholesterol (1 mmol/L)	35-44	1.26 (1.04-1.54)	1.54 (1.07-2.22)	1.54 (1.06-2.24)
Fatal	non-HDL-Cholesterol (1 mmol/L)	45-54	0.92 (0.72-1.16)	0.85 (0.55-1.32)	1.13 (0.97-1.31)
Fatal	non-HDL-Cholesterol (1 mmol/L)	55-64	1.17 (1.04-1.3)	1.33 (1.08-1.63)	1.3 (1.13-1.49)
Fatal	non-HDL-Cholesterol (1 mmol/L)	65-74	1.02 (0.92-1.13)	1.04 (0.86-1.26)	1.17 (1.04-1.33)
Fatal	non-HDL-Cholesterol (1 mmol/L)	75-84	0.97 (0.87-1.08)	0.94 (0.78-1.15)	1.03 (0.88-1.2)
Fatal	non-HDL-Cholesterol (1 mmol/L)	85+	1.08 (0.96-1.21)	1.15 (0.93-1.43)	1.16 (0.96-1.4)
Fatal	Systolic Blood Pressure (10 mmHg)	35-44	1.53 (1.26-1.85)	1.89 (1.41-2.52)	1.86 (1.43-2.41)
Fatal	Systolic Blood Pressure (10 mmHg)	45-54	1.39 (1.3-1.49)	1.64 (1.49-1.82)	1.65 (1.59-1.71)
Fatal	Systolic Blood Pressure (10 mmHg)	55-64	1.38 (1.33-1.43)	1.63 (1.54-1.72)	1.62 (1.54-1.71)
Fatal	Systolic Blood Pressure (10 mmHg)	65-74	1.24 (1.2-1.28)	1.37 (1.31-1.44)	1.38 (1.32-1.45)
Fatal	Systolic Blood Pressure (10 mmHg)	75-84	1.13 (1.08-1.17)	1.2 (1.13-1.27)	1.21 (1.14-1.29)
Fatal	Systolic Blood Pressure (10 mmHg)	85+	1.04 (0.98-1.09)	1.06 (0.97-1.14)	1.08 (0.99-1.17)
Fatal	Total Cholesterol (1 mmol/L)	35-44	1.27 (1.02-1.57)	1.52 (1.04-2.21)	1.46 (1-2.11)
Fatal	Total Cholesterol (1 mmol/L)	45-54	1.03 (0.88-1.21)	1.06 (0.81-1.39)	1.11 (0.99-1.25)
Fatal	Total Cholesterol (1 mmol/L)	55-64	1.14 (1.05-1.24)	1.26 (1.09-1.46)	1.25 (1.09-1.43)
Fatal	Total Cholesterol (1 mmol/L)	65-74	1.08 (1.01-1.16)	1.14 (1.01-1.29)	1.14 (1.01-1.28)
Fatal	Total Cholesterol (1 mmol/L)	75-84	0.97 (0.89-1.06)	0.95 (0.82-1.11)	0.99 (0.85-1.15)
Fatal	Total Cholesterol (1 mmol/L)	85+	1.08 (0.98-1.2)	1.15 (0.96-1.38)	1.16 (0.96-1.38)
Fatal + Non-Fatal	Body Mass Index (5 kg/m2)	35-44	1.47 (0.99-2.19)	1.53 (0.99-2.37)	1.41 (0.93-2.14)
Fatal + Non-Fatal	Body Mass Index (5 kg/m2)	45-54	1.36 (1.16-1.61)	1.41 (1.18-1.68)	1.43 (1.2-1.7)
Fatal + Non-Fatal	Body Mass Index (5 kg/m2)	55-64	1.2 (1.08-1.33)	1.22 (1.09-1.37)	1.24 (1.11-1.38)
Fatal + Non-Fatal	Body Mass Index (5 kg/m2)	65-74	1.23 (1.1-1.36)	1.25 (1.11-1.41)	1.23 (1.09-1.38)
Fatal + Non-Fatal	Body Mass Index (5 kg/m2)	75-84	1.22 (1.02-1.48)	1.25 (1.02-1.53)	1.28 (1.05-1.56)
Fatal + Non-Fatal	Fasting Glucose (1 mmol/L)	35-44	1.2 (0.87-1.65)	1.31 (0.8-2.15)	1.22 (0.77-1.92)
Fatal + Non-Fatal	Fasting Glucose (1 mmol/L)	45-54	1.09 (1.03-1.16)	1.15 (1.05-1.26)	1.15 (1.05-1.26)
Fatal + Non-Fatal	Fasting Glucose (1 mmol/L)	55-64	1.11 (1.07-1.15)	1.18 (1.12-1.24)	1.19 (1.13-1.25)
Fatal + Non-Fatal	Fasting Glucose (1 mmol/L)	65-74	1.09 (1.05-1.13)	1.14 (1.08-1.2)	1.15 (1.09-1.21)
Fatal + Non-Fatal	Fasting Glucose (1 mmol/L)	75-84	1.02 (0.89-1.16)	1.02 (0.83-1.26)	1.07 (0.9-1.27)
Fatal + Non-Fatal	non-HDL-Cholesterol (1 mmol/L)	35-44	1.67 (1.12-2.5)	2.58 (1.23-5.44)	1.41 (0.97-2.06)
Fatal + Non-Fatal	non-HDL-Cholesterol (1 mmol/L)	45-54	1.37 (1.09-1.71)	1.78 (1.17-2.71)	1.42 (1.16-1.73)
Fatal + Non-Fatal	non-HDL-Cholesterol (1 mmol/L)	55-64	1.4 (1.2-1.62)	1.86 (1.41-2.45)	1.47 (1.31-1.64)
Fatal + Non-Fatal	non-HDL-Cholesterol (1 mmol/L)	65-74	1.35 (1.15-1.59)	1.74 (1.29-2.35)	1.22 (1.08-1.39)
Fatal + Non-Fatal	non-HDL-Cholesterol (1 mmol/L)	75-84	1.09 (0.85-1.4)	1.18 (0.74-1.86)	1.19 (0.96-1.47)
Fatal + Non-Fatal	Systolic Blood Pressure (10 mmHg)	35-44	1.39 (1.09-1.76)	1.64 (1.14-2.34)	1.67 (1.19-2.36)
Fatal + Non-Fatal	Systolic Blood Pressure (10 mmHg)	45-54	1.36 (1.29-1.43)	1.59 (1.47-1.72)	1.6 (1.48-1.73)

Fatal + Non-Fatal	Systolic Blood Pressure (10 mmHg)	55-64	1.33 (1.29-1.37)	1.53 (1.46-1.6)	1.53 (1.46-1.6)
Fatal + Non-Fatal	Systolic Blood Pressure (10 mmHg)	65-74	1.24 (1.2-1.29)	1.39 (1.32-1.46)	1.39 (1.32-1.46)
Fatal + Non-Fatal	Systolic Blood Pressure (10 mmHg)	75-84	1.19 (1.12-1.28)	1.3 (1.18-1.44)	1.32 (1.19-1.45)
Fatal + Non-Fatal	Total Cholesterol (1 mmol/L)	35-44	1.4 (1.02-1.94)	1.81 (1.03-3.18)	1.35 (0.94-1.94)
Fatal + Non-Fatal	Total Cholesterol (1 mmol/L)	45-54	1.32 (1.16-1.5)	1.62 (1.29-2.02)	1.37 (1.14-1.64)
Fatal + Non-Fatal	Total Cholesterol (1 mmol/L)	55-64	1.26 (1.18-1.36)	1.5 (1.33-1.7)	1.42 (1.28-1.58)
Fatal + Non-Fatal	Total Cholesterol (1 mmol/L)	65-74	1.19 (1.1-1.29)	1.36 (1.18-1.57)	1.2 (1.06-1.35)
Fatal + Non-Fatal	Total Cholesterol (1 mmol/L)	75-84	1.24 (1.07-1.44)	1.45 (1.12-1.89)	1.16 (0.94-1.42)

All estimates were adjusted by sex and age at risk (i.e., at event); age groups based on age at risk.

Supplementary Figure 1: Comparison of relative risk as per complete-case analysis versus multiple imputation analysis by outcome, risk factor and age group

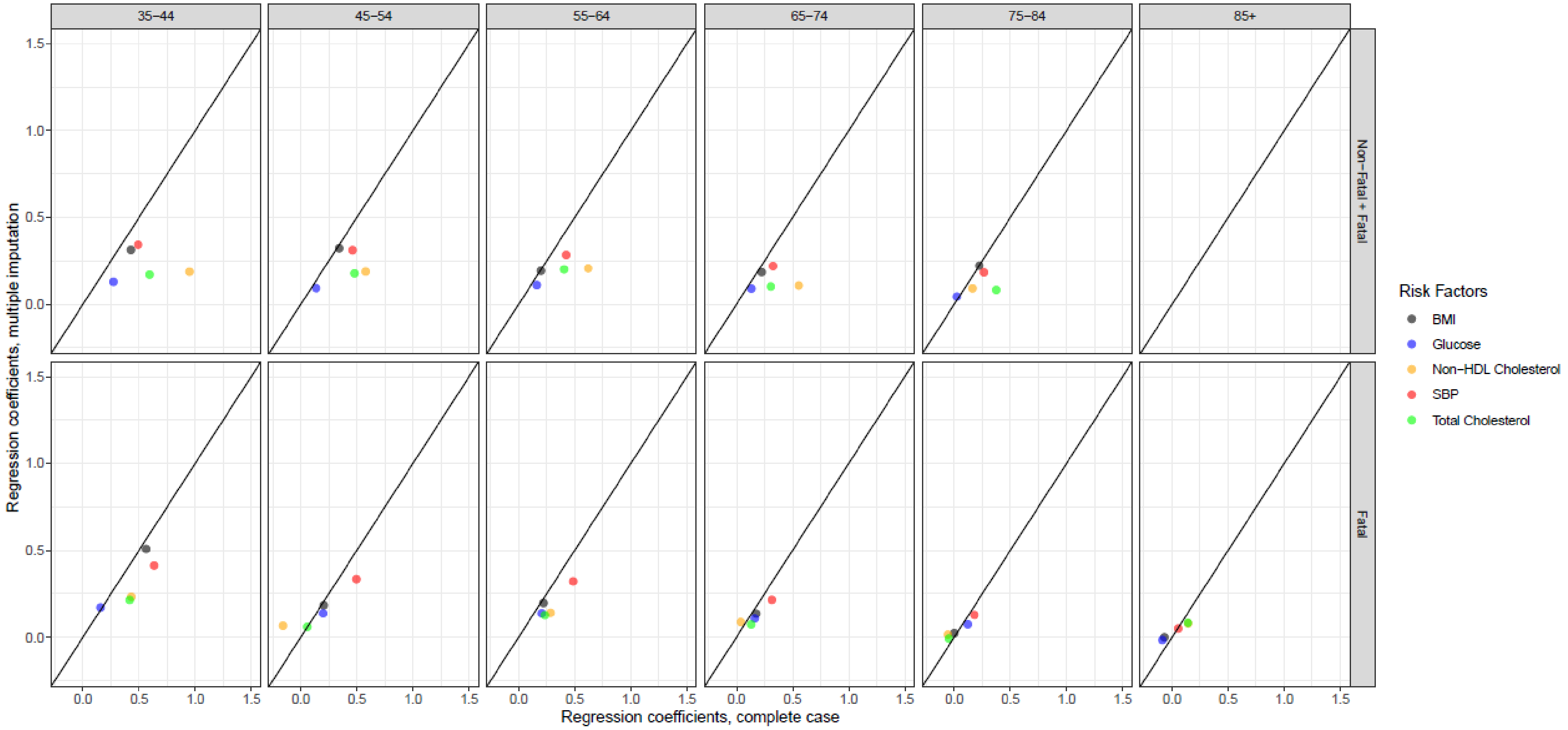

BMI: body mass index; SBP: systolic blood pressure.

Supplementary Table 6: Relative risk estimates (95% CI) for each cardio-metabolic risk factor on fatal cardiovascular events by sub-region (Central America & Caribbean vs South America) and age group

Risk factor	Age group	RR (95% CI)	
		Central America & Caribbean	South America
Body Mass Index (5 kg/m^2)	35-44	0.74 (0.23-2.4)	insufficient events
	45-54	0.64 (0.29-1.42)	0.81 (0.28-2.33)
	55-64	1.23 (0.9-1.7)	1.52 (0.78-2.96)
	65-74	1.65 (1.43-1.9)	0.97 (0.56-1.66)
	75-84	1.03 (0.68-1.57)	1.09 (0.61-1.95)
	85+	1.09 (0.73-1.64)	1.07 (0.38-3)
Systolic Blood Pressure (10 mmHg)	35-44	1.43 (0.51-4.02)	1.52 (0.31-7.4)
	45-54	1.72 (1.22-2.42)	1.6 (1.16-2.23)
	55-64	1.63 (1.44-1.84)	1.92 (1.37-2.69)
	65-74	1.58 (1.55-1.62)	1.35 (1.05-1.73)
	75-84	1.26 (1.07-1.48)	1.62 (1.23-2.14)
	85+	1.25 (1.01-1.55)	1.15 (0.86-1.54)
Fasting Glucose (1 mmol/L)	35-44	insufficient events	insufficient events
	45-54	1.03 (0.49-2.13)	0.5 (0.09-2.87)
	55-64	1.11 (0.91-1.35)	1.49 (1.15-1.94)
	65-74	1.21 (1.19-1.24)	1.18 (0.99-1.41)
	75-84	1.08 (0.88-1.34)	1.18 (0.66-2.1)
	85+	0.7 (0.36-1.38)	1.53 (0.94-2.49)
Total Cholesterol (1 mmol/L)	35-44	insufficient events	insufficient events
	45-54	0.86 (0.35-2.11)	0.99 (0.26-3.74)
	55-64	1.44 (1.11-1.86)	0.91 (0.36-2.31)
	65-74	1.28 (1.16-1.42)	1.83 (0.92-3.63)
	75-84	1 (0.67-1.48)	0.75 (0.32-1.75)
	85+	0.81 (0.59-1.13)	0.78 (0.27-2.26)
Non-HDL-Cholesterol (1 mmol/L)	35-44	insufficient events	insufficient events
	45-54	0.79 (0.29-2.13)	1.16 (0.28-4.84)
	55-64	1.46 (1.11-1.93)	1.22 (0.48-3.15)
	65-74	1.35 (1.18-1.54)	1.94 (0.93-4.06)
	75-84	0.98 (0.64-1.5)	0.83 (0.33-2.06)
	85+	0.82 (0.58-1.17)	0.81 (0.26-2.53)

All relative risk estimates were adjusted by regression dilution bias. Only estimated for fatal outcomes. Results shown are from the multiple imputation model.

Supplementary Figure 2: Interpolation of age-specific relative risks for each cardio-metabolic risk factor

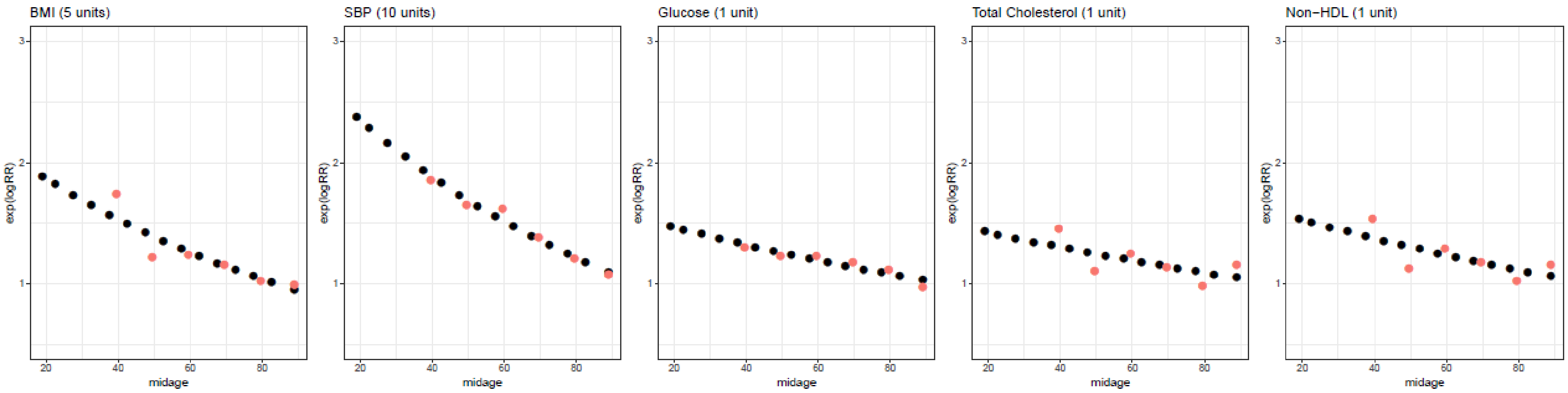

Red dots represented the estimates as per the multiple imputation analysis; the black dots are the coefficients after the interpolation analysis (i.e. the age-specific regression coefficients in 5-year age groups, which were used to estimate population attributable fractions).

Supplementary Table 7: Age-specific relative risks (95% confidence interval) for fatal cardiovascular diseases estimated through interpolation

Age group	RR (95% CI)
Body mass index (5 kg/m^2)	
20-24	1.82 (1.4-2.36)
25-29	1.73 (1.37-2.19)
30-34	1.65 (1.34-2.04)
35-39	1.57 (1.31-1.89)
40-44	1.5 (1.28-1.76)
45-49	1.43 (1.24-1.64)
50-54	1.36 (1.21-1.52)
55-59	1.29 (1.18-1.42)
60-64	1.23 (1.14-1.33)
65-69	1.17 (1.1-1.26)
70-74	1.12 (1.05-1.19)
75-79	1.06 (0.99-1.15)
80-84	1.01 (0.93-1.11)
85+	0.95 (0.85-1.07)
Systolic blood pressure (10 mmHg)	
20-24	2.29 (1.85-2.83)
25-29	2.17 (1.79-2.62)
30-34	2.05 (1.73-2.43)
35-39	1.94 (1.67-2.26)
40-44	1.84 (1.61-2.09)
45-49	1.74 (1.55-1.94)
50-54	1.64 (1.5-1.8)
55-59	1.56 (1.45-1.67)
60-64	1.47 (1.39-1.56)
65-69	1.39 (1.34-1.45)
70-74	1.32 (1.28-1.36)
75-79	1.25 (1.2-1.29)
80-84	1.18 (1.13-1.24)
85+	1.1 (1.03-1.18)
Fasting glucose (1 mmol/L)	
20-24	1.45 (1.09-1.93)
25-29	1.41 (1.09-1.83)
30-34	1.38 (1.09-1.74)
35-39	1.34 (1.09-1.65)
40-44	1.31 (1.1-1.56)
45-49	1.27 (1.1-1.48)
50-54	1.24 (1.1-1.41)
55-59	1.21 (1.1-1.34)
60-64	1.18 (1.1-1.27)
65-69	1.15 (1.09-1.22)
70-74	1.12 (1.07-1.17)
75-79	1.09 (1.04-1.15)
80-84	1.07 (0.99-1.14)
85+	1.03 (0.93-1.14)
Total cholesterol (1 mmol/L)	
20-24	1.41 (1.03-1.92)
25-29	1.38 (1.04-1.82)
30-34	1.35 (1.05-1.73)
35-39	1.32 (1.06-1.64)
40-44	1.29 (1.07-1.56)
45-49	1.26 (1.07-1.48)
50-54	1.23 (1.08-1.41)
55-59	1.21 (1.08-1.35)
60-64	1.18 (1.08-1.29)
65-69	1.16 (1.07-1.25)
70-74	1.13 (1.05-1.22)
75-79	1.11 (1.01-1.2)
80-84	1.08 (0.97-1.2)
85+	1.05 (0.92-1.21)
Non-HDL cholesterol (1 mmol/L)	
20-24	1.51 (1.1-2.07)
25-29	1.47 (1.11-1.95)
30-34	1.43 (1.11-1.84)
35-39	1.4 (1.12-1.74)
40-44	1.36 (1.12-1.65)
45-49	1.32 (1.12-1.56)

50-54	1.29 (1.12-1.48)
55-59	1.26 (1.12-1.4)
60-64	1.22 (1.12-1.34)
65-69	1.19 (1.1-1.29)
70-74	1.16 (1.07-1.25)
75-79	1.13 (1.03-1.24)
80-84	1.1 (0.99-1.23)
85+	1.06 (0.92-1.23)

These age-specific relative risks were used to compute the population attributable fractions. The age 88.88 was assumed for the open age group 85+.

Supplementary Table 8: Number of cardiovascular disease (CVD) deaths and population size by country, age group and gender[1]

Country	Age group	CVD deaths, men	Population size, men	CVD deaths, women	Population size, women
Antigua and Barbuda	20-24	0.28	3602	0.14	3541
	25-29	0.28	3391	0.18	3549
	30-34	0.51	3262	0.29	3602
	35-39	0.88	3166	0.52	3535
	40-44	1.13	3111	1.04	3554
	45-49	2.42	3133	2.09	3479
	50-54	4.37	2888	3.34	3176
	55-59	7.77	2339	4.43	2564
	60-64	7.81	1814	6.41	1938
	65-69	9.35	1394	8.41	1485
	70-74	11.74	995	10.07	1071
	75-79	11.7	610	12.39	732
	80-84	10.81	386	16.44	535
	85+	15.06	415	36.37	561
Argentina	20-24	121.85	1774280	75.88	1773776
	25-29	159.05	1721964	98.71	1760837
	30-34	252.34	1628984	148.19	1685307
	35-39	414.25	1577535	231.23	1638560
	40-44	707.82	1437286	381.52	1499596
	45-49	1253.42	1246206	585.91	1309546
	50-54	2017.94	1072935	854.46	1144903
	55-59	3157.02	980043	1347.36	1066069
	60-64	4660.08	873303	2176.16	980874
	65-69	6093.42	723974	3254.72	852330
	70-74	7136.86	547692	4655.84	695489
	75-79	7575.33	371242	6671.65	528319
	80-84	7846.63	227795	9628.12	381176
	85+	10575.89	171854	23341.98	385285
Bahamas	20-24	1.58	15690	0.89	15860
	25-29	2.78	14395	1.8	15105
	30-34	4.69	13914	3.18	14897
	35-39	7.86	13373	5.15	14516
	40-44	14.2	13608	7.95	14642
	45-49	26.38	12959	12.28	13876
	50-54	36.2	11656	19.63	12702
	55-59	44.96	9292	24.67	10477
	60-64	44.47	6885	28.89	7954
	65-69	48.73	4743	37.12	5505
	70-74	44.76	3130	43.56	3988
	75-79	46.39	2134	49.44	2854
	80-84	41.91	1295	55.53	1751
	85+	47.93	947	94.97	1509

Barbados	20-24	0.39	9411	0.58	9478
	25-29	0.91	9840	0.61	10160
	30-34	1.62	9538	1.11	10115
	35-39	2.69	9696	1.97	10424
	40-44	4.2	9841	3.21	10581
	45-49	11.58	10121	6.23	11099
	50-54	16.1	10234	8.86	11538
	55-59	25.35	9831	14.63	11200
	60-64	32.17	8661	22.55	9865
	65-69	41.01	6563	29.28	7549
	70-74	46.86	4945	41.47	6144
	75-79	43.66	3393	56.9	4396
	80-84	53.33	2263	75.01	3154
	85+	98.66	1867	198.2	3160
Belize	20-24	1.41	18719	0.89	19194
	25-29	2.16	16926	1.45	17559
	30-34	3.74	14445	1.98	15299
	35-39	4.78	12760	2	13591
	40-44	6.87	11258	3.8	11862
	45-49	10.56	9729	6.84	9984
	50-54	14.05	8283	9.63	8176
	55-59	24.27	6683	13.32	6465
	60-64	25.63	4924	14.64	4687
	65-69	26.52	3579	16.03	3243
	70-74	24.76	2421	16.8	2144
	75-79	26.46	1671	19.71	1506
	80-84	31.16	1086	24.72	1057
	85+	41.83	915	44.23	1092
Bermuda	20-24	0.08	1646	0.03	1781
	25-29	0.1	1840	0.04	2022
	30-34	0.2	2133	0.07	2276
	35-39	0.42	2321	0.11	2366
	40-44	0.88	2402	0.21	2391
	45-49	1.97	2548	0.46	2557
	50-54	3.8	2631	0.91	2773
	55-59	5.85	2499	1.41	2820
	60-64	8.37	2112	2.16	2459
	65-69	10.8	1667	3.08	1973
	70-74	14.24	1283	4.89	1598
	75-79	15.8	865	7.8	1196
	80-84	18.87	549	13.31	865
	85+	27.65	418	40.87	959
Bolivia	20-24	47.17	537291	36.95	527051
	25-29	47.74	492459	42.37	485263
	30-34	53.56	421816	56.48	422152
	35-39	77.6	375537	85.08	380939

	40-44	128.16	323898	132.81	327752
	45-49	210.34	270530	208	272540
	50-54	347.69	227475	291.42	232480
	55-59	524.67	194229	383.81	202035
	60-64	729.38	157863	547.44	168944
	65-69	910.29	122278	737.54	135742
	70-74	1056.99	88779	949.53	100036
	75-79	1127.27	58059	1156.75	67795
	80-84	1070.28	32079	1219.67	41551
	85+	1213.15	19468	1695.44	29786
Brazil	20-24	672.18	8735943	412.02	8679302
	25-29	992.65	8508880	590.65	8715050
	30-34	1759.4	8653420	1067.42	8966219
	35-39	3008.92	8292903	1970.38	8668900
	40-44	4784.89	7222247	3211.32	7646776
	45-49	7815.85	6424010	4981.78	6854141
	50-54	12392.49	5889456	7317.1	6439981
	55-59	17418.25	4999295	10123.14	5612871
	60-64	21472.12	3986395	13400.02	4624595
	65-69	24946.71	3081566	16712.76	3597163
	70-74	25981.13	2117602	19980.25	2648631
	75-79	26913.56	1446409	24813	1906888
	80-84	23762.82	841817	27558.35	1265399
	85+	31232.8	693110	51055.65	1228797
Chile	20-24	27.95	721371	14.19	702553
	25-29	44.81	761034	20.13	749142
	30-34	68.39	661390	28.97	662814
	35-39	117.77	610931	57.78	626049
	40-44	216.89	600946	106.31	624800
	45-49	372.08	574306	180.51	609213
	50-54	681.64	578976	307.83	623638
	55-59	967.64	504868	467.49	553380
	60-64	1277.21	403031	643.9	450321
	65-69	1624.36	306368	923.04	352454
	70-74	1949.06	235381	1301.37	285128
	75-79	2079.06	157087	1787.74	209343
	80-84	2185.45	97760	2404.58	143601
	85+	3499.95	84061	6504.57	143995
Colombia	20-24	117.88	2218517	68.01	2190718
	25-29	164.69	2019288	82.67	2019943
	30-34	219.66	1791444	112.41	1846411
	35-39	317.15	1657780	188.4	1751108
	40-44	463.23	1466433	313.56	1582034
	45-49	753.53	1386162	513.75	1532364
	50-54	1360.2	1338359	887.58	1496516
	55-59	2011.98	1144521	1328.6	1303933

	60-64	2758.2	903232	1869.01	1045368
	65-69	3472.81	700574	2467.45	817430
	70-74	4166.44	515701	3367.11	617471
	75-79	4802.98	370689	4577.52	469747
	80-84	5247.61	247116	5894.51	328479
	85+	8265.97	231290	11859.61	319918
Costa Rica	20-24	10.76	198374	6.86	204929
	25-29	16.59	199738	9.72	211430
	30-34	25.02	183705	12.74	199321
	35-39	40.31	165047	19.65	181387
	40-44	64.8	137995	26.98	151462
	45-49	105.65	131717	48.54	144411
	50-54	193.89	127396	78.73	141809
	55-59	258.72	112119	116.46	126697
	60-64	344.86	86962	163.16	98805
	65-69	380.79	64917	205.51	73992
	70-74	447.4	47085	279.44	53283
	75-79	490.11	32389	337.08	37847
	80-84	513.33	20731	467.21	26386
	85+	994.96	20866	1175.1	31053
Cuba	20-24	14.51	368153	7.17	342724
	25-29	30.71	428672	13.61	401769
	30-34	49.58	388229	23.7	369311
	35-39	80.52	317035	34.37	307149
	40-44	202.16	417258	97.01	417836
	45-49	472.25	498745	234.71	509619
	50-54	896.28	510291	447.52	532883
	55-59	1116.97	367846	579.8	389804
	60-64	1468.99	283941	896.92	307235
	65-69	1981.47	251526	1310.02	272565
	70-74	2601.61	201290	1907.73	228220
	75-79	2983.95	148150	2650.41	171175
	80-84	2978.5	91898	3050.15	112028
	85+	4835.8	86946	6603.61	124547
Dominica	20-24	0.25	2730	0.28	2568
	25-29	0.32	2617	0.36	2546
	30-34	0.57	2510	0.46	2462
	35-39	0.71	2218	0.45	2098
	40-44	1.7	2055	0.94	1917
	45-49	2.9	2368	1.72	2261
	50-54	4.53	2369	2.19	2155
	55-59	7.22	2131	2.6	1774
	60-64	10.05	1786	5.3	1512
	65-69	9.7	1203	7.02	1183
	70-74	12.52	896	10.49	975
	75-79	16.69	784	18.17	863

	80-84	15.86	509	24.76	662
	85+	18.43	336	46.13	711
Dominican Republic	20-24	34.88	489970	34.01	488554
	25-29	60.13	462594	49.12	454500
	30-34	107.33	413038	80.07	398304
	35-39	189.71	375825	130.07	361025
	40-44	319.87	332499	194.53	323928
	45-49	531.3	290414	302.15	287538
	50-54	853.93	251168	447.54	252222
	55-59	1015.32	203107	525.87	205535
	60-64	1372.43	159484	753.41	164326
	65-69	1476.18	123879	928.21	131550
	70-74	1722.2	90158	1203.46	97994
	75-79	1521.66	61663	1240.32	69959
	80-84	1524.01	40962	1494.11	48865
	85+	2613.86	35880	3136.58	49589
Ecuador	20-24	93.08	747563	42.59	743881
	25-29	113.32	676046	52.49	688893
	30-34	131.72	606372	74.59	628094
	35-39	156.93	560460	102.33	587353
	40-44	229.38	491322	140.79	515402
	45-49	309.52	422669	208.16	443394
	50-54	459.9	379770	307.84	399000
	55-59	584.67	316478	398.99	332556
	60-64	730.29	256863	548.86	268913
	65-69	875.65	206356	673.44	215313
	70-74	1042.96	154822	848.15	165389
	75-79	1173.86	106803	1114.95	117650
	80-84	1265.01	66145	1397.76	75275
	85+	2975.05	54873	3421.78	70271
El Salvador	20-24	30.82	296559	16.91	318536
	25-29	41.77	239256	17.85	272334
	30-34	56.15	187763	24.12	229527
	35-39	73.4	174711	38.19	225476
	40-44	96.78	155011	61.52	208726
	45-49	141.61	134941	94.79	183121
	50-54	190.62	115811	144.89	159672
	55-59	251.31	97485	192.89	133501
	60-64	319.96	82163	270.09	110385
	65-69	395.32	68541	354.1	89618
	70-74	525.64	53381	469.13	69554
	75-79	595.84	38918	649.94	52841
	80-84	694.25	26002	811.53	35853
	85+	1384.21	23185	1837.84	37080
Grenada	20-24	0.18	5109	0.35	4684
	25-29	0.68	4769	0.5	4491

	30-34	0.71	3697	0.65	3515
	35-39	1.44	3126	0.98	2961
	40-44	2.6	3337	1.74	3196
	45-49	4.58	3679	3.04	3482
	50-54	10.21	3593	5.29	3322
	55-59	15.42	3034	6.89	2760
	60-64	15.29	2211	8.51	2038
	65-69	15.32	1568	12.11	1539
	70-74	20.32	1677	21.28	1635
	75-79	27.24	1776	38.52	1723
	80-84	26.1	1161	49.1	1254
	85+	34.78	784	86.07	1148
Guatemala	20-24	101.6	906852	65.48	907536
	25-29	125.98	749827	69.32	793358
	30-34	156.54	581904	86.95	663723
	35-39	187.46	496965	124.02	606202
	40-44	195.04	372434	140.83	474392
	45-49	243.51	292227	184.86	372705
	50-54	289.59	241860	254.59	301012
	55-59	362.23	211010	346.34	255458
	60-64	505.44	175505	461.34	206041
	65-69	637.13	136868	551.76	157439
	70-74	812.39	103733	653.32	118864
	75-79	1058.97	78899	836.98	94840
	80-84	1302.08	51488	1345.46	67231
	85+	1600.12	26380	2003.3	39289
Guyana	20-24	4.02	36194	4.09	37045
	25-29	6	27321	4.8	29080
	30-34	7.69	23021	6.64	24860
	35-39	14.21	23649	10.95	25141
	40-44	27.01	22860	20.23	23805
	45-49	50.98	21440	36.15	21402
	50-54	85.59	19175	56.35	19479
	55-59	131.82	16164	78.22	16805
	60-64	146.35	11916	103.6	13281
	65-69	156.22	8671	115.28	9506
	70-74	133.61	5079	110.68	5744
	75-79	113.98	3315	128.31	4203
	80-84	89.31	1795	119.98	2504
	85+	83.78	1383	176.91	2133
Haiti	20-24	37.29	523674	72.41	560315
	25-29	58.8	501821	127.85	565801
	30-34	97.56	448509	199.34	529277
	35-39	177.3	372688	319.5	448174
	40-44	302.11	299654	486.82	350073
	45-49	478.78	238181	669.09	267521

	50-54	725.95	196992	948.83	218509
	55-59	997.7	161294	1172.26	179790
	60-64	1258.23	125470	1517.62	143261
	65-69	1389.63	89639	1751	103324
	70-74	1400.26	59826	1832.13	67834
	75-79	1222.62	36566	1806.24	43065
	80-84	994.37	19961	1500.2	22759
	85+	834.66	10818	1145.44	10876
Honduras	20-24	36.93	459889	33.22	496083
	25-29	70.78	375063	49.52	427736
	30-34	84.62	301643	72.17	352784
	35-39	102.72	263378	119.32	307615
	40-44	139.89	225686	182.29	257061
	45-49	183.22	185514	228.46	205753
	50-54	270.43	152037	299.32	167725
	55-59	393.25	127218	390.46	140187
	60-64	525.13	102301	521.89	111741
	65-69	657.21	81434	691.27	89679
	70-74	758.45	59364	853.03	64925
	75-79	795.7	42853	1090.03	46132
	80-84	811.51	26497	1167.05	27277
	85+	936.27	20612	1540.51	18716
Jamaica	20-24	12.22	137422	5.98	134164
	25-29	13.37	124425	6.99	127519
	30-34	18.99	103808	11.34	112122
	35-39	29.54	87737	21.63	98455
	40-44	49.13	83056	32.65	89453
	45-49	85.92	82952	56.88	83017
	50-54	148.66	76135	83.49	73888
	55-59	209.08	64650	120.67	66447
	60-64	269.29	49692	175.21	50841
	65-69	349.9	39305	215.14	39920
	70-74	425.56	28568	280.31	30111
	75-79	420.35	19401	356.77	22710
	80-84	432.3	13138	485.79	17685
	85+	671.71	14139	1266.05	21069
Mexico	20-24	375.78	5391865	198.12	5498719
	25-29	538.88	4946472	257.05	5181242
	30-34	795.6	4581146	347.31	4912491
	35-39	1197.1	4265401	557.34	4638102
	40-44	1970.33	4039845	950.9	4405960
	45-49	2863.34	3596269	1443.51	3951439
	50-54	4264.83	3029829	2184.83	3352498
	55-59	5650.4	2510437	3122.78	2811799
	60-64	7038.05	1995222	4401.27	2239918
	65-69	8207.7	1477240	5674.99	1673280

	70-74	9401.69	1124723	7534.64	1292665
	75-79	10590.19	769459	9911.34	918200
	80-84	11359.42	476211	12293.72	587494
	85+	19992.58	392614	27566.27	498125
Nicaragua	20-24	14.42	311295	12.51	306727
	25-29	18.64	283966	11.63	287679
	30-34	33.34	246082	18.06	255244
	35-39	42.53	209965	32.81	223331
	40-44	60.43	170184	47.52	187511
	45-49	77.11	139743	70.77	159461
	50-54	126.78	116555	95.99	135750
	55-59	174.58	95855	121.52	112041
	60-64	221.9	75610	186.8	87692
	65-69	264.53	56408	224.88	65037
	70-74	293.29	39980	287.14	46406
	75-79	342.82	28409	378.2	33704
	80-84	394.23	19277	497.95	23585
	85+	636.43	19876	1312.96	22822
Panama	20-24	8.66	160463	5.49	158007
	25-29	10.86	147230	8.71	146183
	30-34	19.95	141599	11.66	141368
	35-39	26.58	132717	15.37	133777
	40-44	43.03	125121	30.39	125525
	45-49	72.53	118176	39.92	117323
	50-54	118.84	104213	51.81	104084
	55-59	163.82	84121	77.76	85310
	60-64	213.45	69039	102.73	69894
	65-69	262.12	55338	146.44	55819
	70-74	318.81	41200	194.66	42834
	75-79	380.9	29774	255.4	32017
	80-84	406.75	18244	332.98	21472
	85+	768.88	17883	1121.94	23998
Paraguay	20-24	17.24	334921	9.19	324574
	25-29	24.29	305976	11.18	296022
	30-34	40.47	278866	18.88	268861
	35-39	55.19	235609	33.59	228139
	40-44	103.78	190722	62.21	187238
	45-49	170.24	164080	104.49	162984
	50-54	287.23	148566	163.11	147548
	55-59	440.13	128029	223.41	126934
	60-64	544.9	101215	291.48	101582
	65-69	637.77	73512	370.78	76236
	70-74	685.36	50276	451.04	55601
	75-79	696.26	33490	569.45	40001
	80-84	695.84	20100	701.1	26524
	85+	955.35	16487	1431.61	26653

Peru	20-24	120.47	1424675	68.26	1414266
	25-29	159.31	1342417	78.98	1371693
	30-34	187.52	1218414	93.45	1256428
	35-39	223.85	1131287	123.57	1175651
	40-44	293.09	1012749	179.19	1051796
	45-49	388.54	897415	252.47	925600
	50-54	542.85	778334	335.36	800712
	55-59	679.05	646566	431.21	667042
	60-64	865.23	520676	590.93	539236
	65-69	1104.44	405683	773.17	421151
	70-74	1346.25	310018	1006.46	321503
	75-79	1739.9	225594	1390.26	237159
	80-84	2151.78	154812	1851.09	172076
	85+	5012.15	152102	5409.99	186323
Puerto Rico	20-24	5.42	129267	2.67	127734
	25-29	7.97	120522	3.86	123925
	30-34	13.09	109036	5.92	116546
	35-39	26.28	113658	12.31	124144
	40-44	46.96	112673	21.24	122447
	45-49	81.87	111413	35.94	122711
	50-54	138.41	115959	72.04	131993
	55-59	227.41	108196	111.38	126853
	60-64	307.91	100803	160.64	119661
	65-69	441.52	96678	259.72	116115
	70-74	548.83	78553	377.25	96394
	75-79	590.7	54955	499.31	69758
	80-84	660.82	35947	707.14	49565
	85+	1238.46	33793	1834.1	58845
Saint Lucia	20-24	0.55	7412	0.33	7257
	25-29	0.9	6807	0.4	6722
	30-34	1.58	6456	0.7	6555
	35-39	2.26	6501	1.02	6569
	40-44	4.66	6416	2.64	6557
	45-49	7.37	6461	4.61	6570
	50-54	12.26	6055	7.74	5906
	55-59	17.78	4744	8.88	4867
	60-64	18.46	3545	13.49	3697
	65-69	20.9	2741	14.91	2833
	70-74	25.41	2234	23.27	2295
	75-79	27.68	1489	25.72	1712
	80-84	26.75	908	34.26	1190
	85+	41.06	818	75.66	1135
Saint Vincent and the Grenadines	20-24	0.47	4650	0.28	4383
	25-29	0.46	4162	0.56	4041
	30-34	1.22	3909	0.84	3919
	35-39	1.95	3909	1.25	3986

	40-44	3.88	3904	1.74	3800
	45-49	6.56	3885	3.96	3545
	50-54	8.93	3746	5.32	3334
	55-59	14.01	3381	5.57	3029
	60-64	17.49	2701	10.85	2464
	65-69	19.76	1838	11.97	1705
	70-74	24.5	1397	16.01	1317
	75-79	28.56	1086	23.06	1060
	80-84	27.45	823	29.03	857
	85+	27.86	564	53.38	813
Suriname	20-24	1.5	21921	1.23	22005
	25-29	2.81	20913	2.46	22005
	30-34	5.42	21131	3.57	21908
	35-39	9.96	19117	6.18	19354
	40-44	17.1	18156	8.56	18234
	45-49	33.09	18920	17.55	19191
	50-54	63.97	17909	27.34	18138
	55-59	79.79	14710	40.19	15035
	60-64	84.96	10364	49.01	11291
	65-69	93.79	7093	55.77	8409
	70-74	88.67	5217	75.93	6561
	75-79	94.62	3756	95.46	4830
	80-84	88.8	2242	105.67	3175
	85+	88.83	1509	146.19	2543
Trinidad and Tobago	20-24	3.79	47040	2.57	46086
	25-29	7.63	54367	4.32	53432
	30-34	16.69	61664	7.06	60259
	35-39	24.41	56017	12.43	53879
	40-44	39.72	47787	18.3	45761
	45-49	66.82	43360	33.39	41961
	50-54	124.19	46710	65.41	45904
	55-59	190.58	43623	100.02	43420
	60-64	252.05	36214	152.7	36883
	65-69	297.47	28561	178.06	29394
	70-74	311.58	19392	210.09	21392
	75-79	274.69	11488	238.26	14058
	80-84	231.28	6631	264.44	9242
	85+	289.76	5220	491.42	9376
Uruguay	20-24	6.24	129214	3.74	128866
	25-29	7.35	115213	3.35	119430
	30-34	11.22	112585	7.76	117884
	35-39	23.19	114319	14.63	121165
	40-44	45.65	109185	27.48	115618
	45-49	75.75	99250	37.39	106175
	50-54	140.75	94777	64.92	103902
	55-59	242.55	90328	109.57	100460

	60-64	347.79	79626	166.95	91766
	65-69	468.72	64694	258.63	78786
	70-74	583.07	51497	397.44	68418
	75-79	678.52	38752	628.84	58379
	80-84	752.19	26081	1008.78	46879
	85+	1122.02	21284	2665.04	51537
Venezuela	20-24	102.82	1341461	56.88	1326042
	25-29	174.63	1365827	87.08	1391955
	30-34	256.52	1222640	117.83	1239823
	35-39	424.18	1164175	199.01	1165482
	40-44	667.95	1006125	324.65	1010340
	45-49	1099.43	882019	515.51	884552
	50-54	1749.16	766409	768.41	798122
	55-59	2476.78	655939	1100.19	704563
	60-64	3165.02	528425	1526.65	583829
	65-69	3583.13	401934	1972.71	448500
	70-74	3577.95	256369	2283.93	296731
	75-79	3706.91	165453	2843.82	203912
	80-84	3506.7	96524	3450.24	134625
	85+	5092.66	80230	7708.52	137668

[1] Global Burden of Diseases/Institute for Health Metrics. URL: http://ghdx.healthdata.org/record/ihme-data/gbd-2017-population-estimates-1950-2017

Supplementary Figure 3: Country charts (alphabetical order)

Antigua and Barbuda

(Caribbean)

Legend: BMI = body mass index;
SBP = systolic blood pressure;
TC = total cholesterol;
Non−HDL = Non−HDL cholesterol.
Upper values are the largest
observed across countries,
risk factor− and sex−specific.
Sex− and age−specific results
are available through authors.

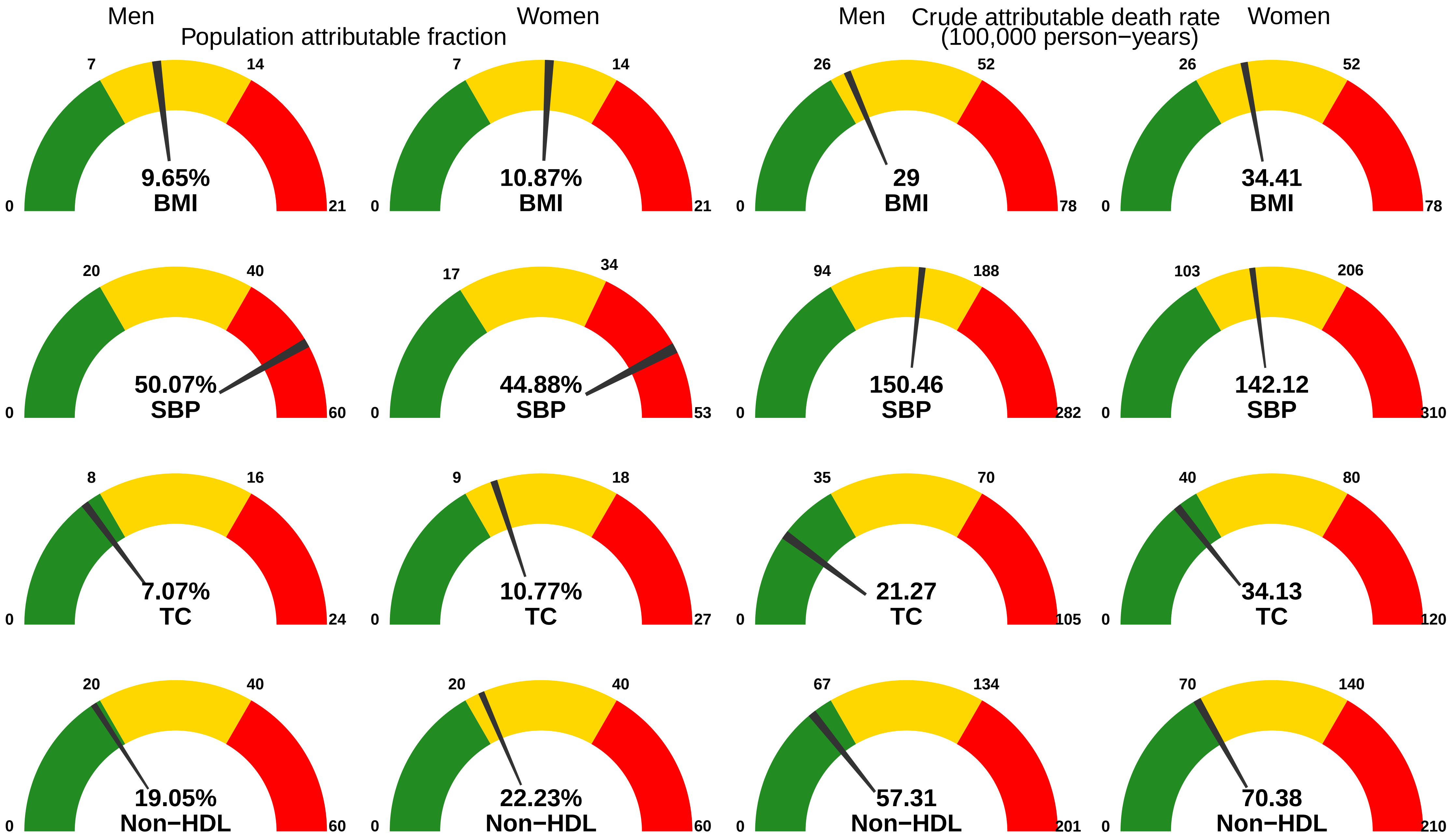

Argentina

(Southern and Tropical Latin America)

Legend: BMI = body mass index;
SBP = systolic blood pressure;
TC = total cholesterol;
Non−HDL = Non−HDL cholesterol.
Upper values are the largest observed across countries, risk factor− and sex−specific.
Sex− and age−specific results are available through authors.

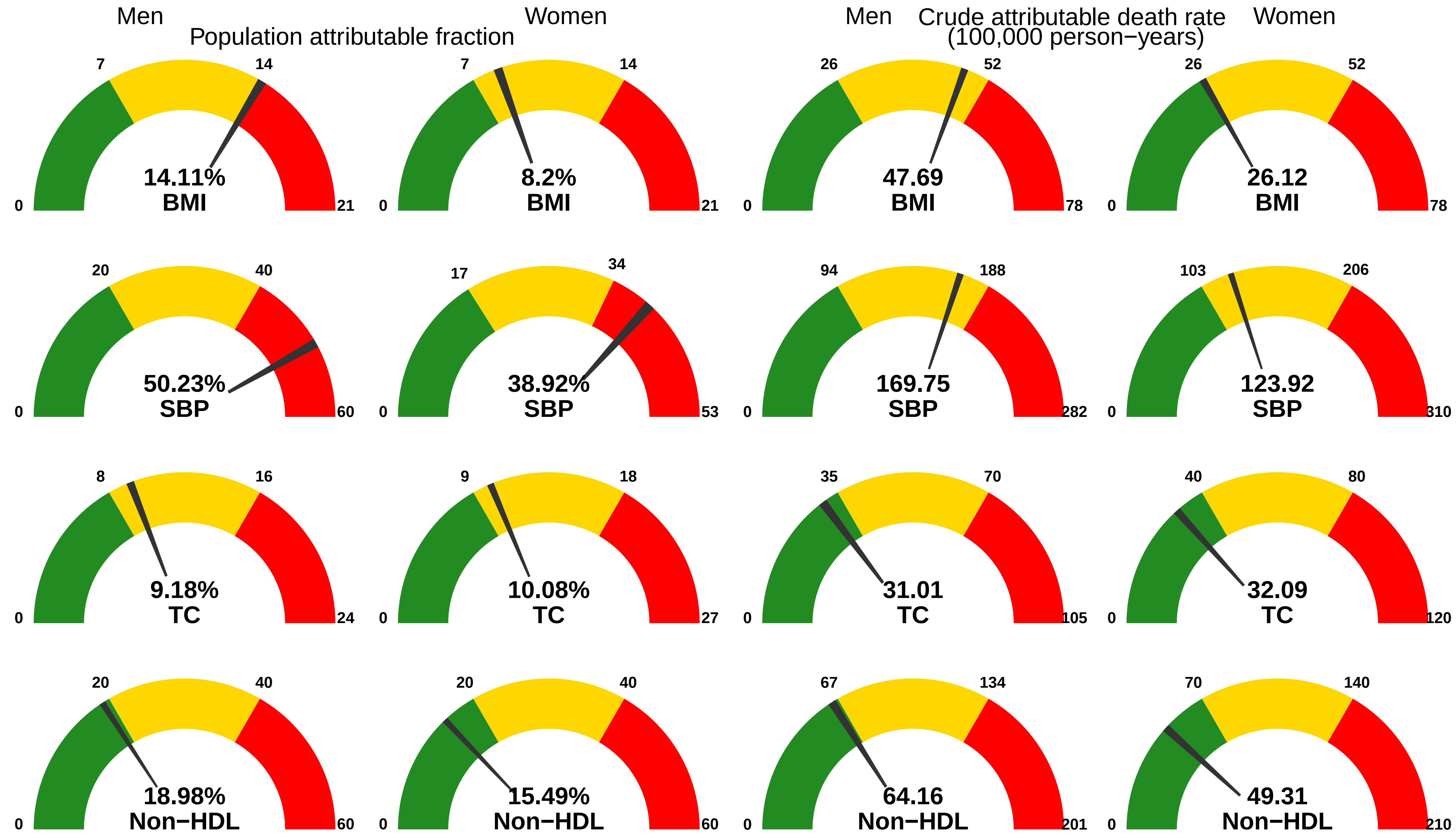

Bahamas
(Caribbean)

Legend: BMI = body mass index;
SBP = systolic blood pressure;
TC = total cholesterol;
Non−HDL = Non−HDL cholesterol.
Upper values are the largest
observed across countries,
risk factor− and sex−specific.
Sex− and age−specific results
are available through authors.

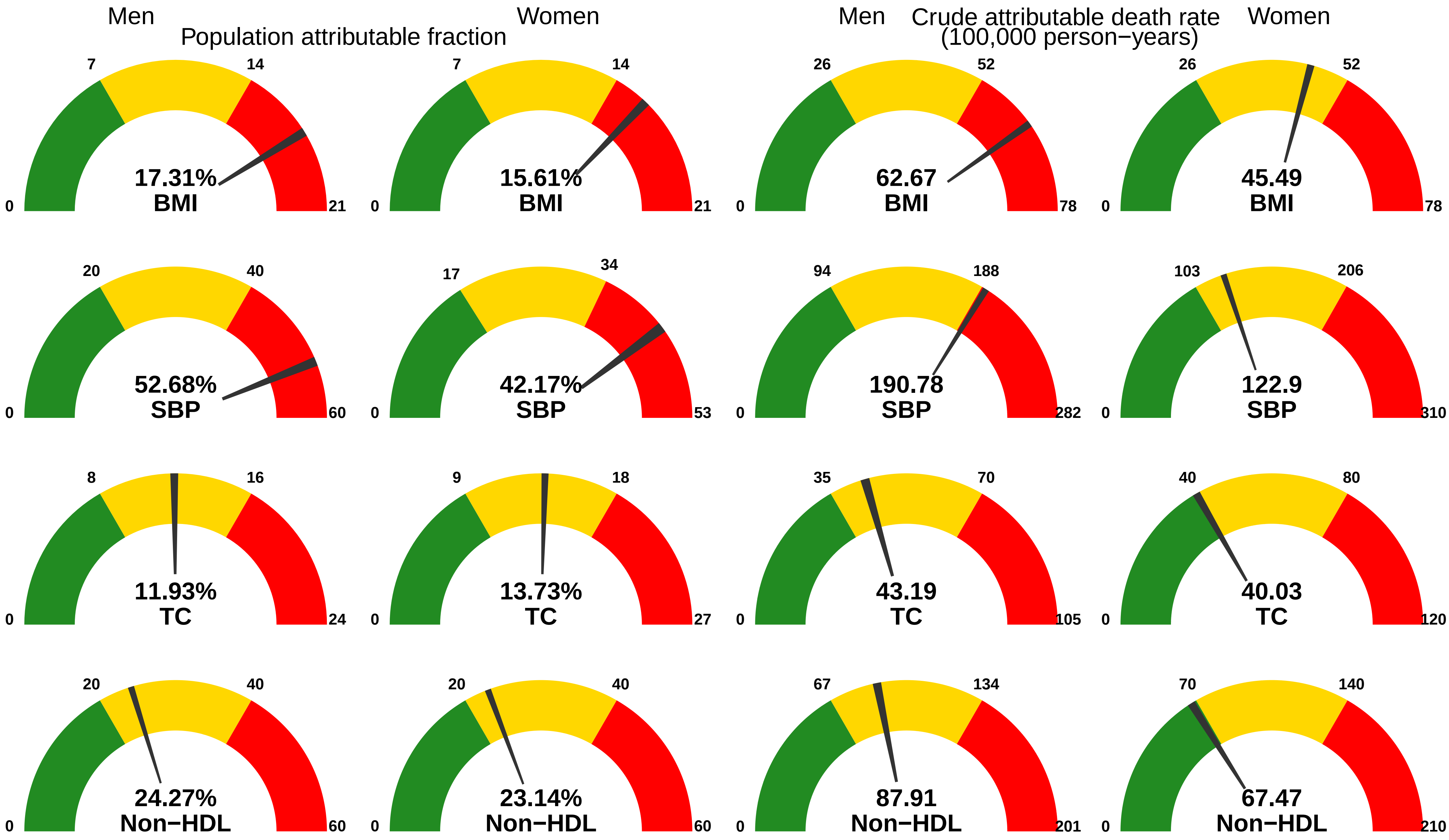

Barbados

(Caribbean)

Legend: BMI = body mass index;
SBP = systolic blood pressure;
TC = total cholesterol;
Non−HDL = Non−HDL cholesterol.
Upper values are the largest observed across countries, risk factor− and sex−specific.
Sex− and age−specific results are available through authors.

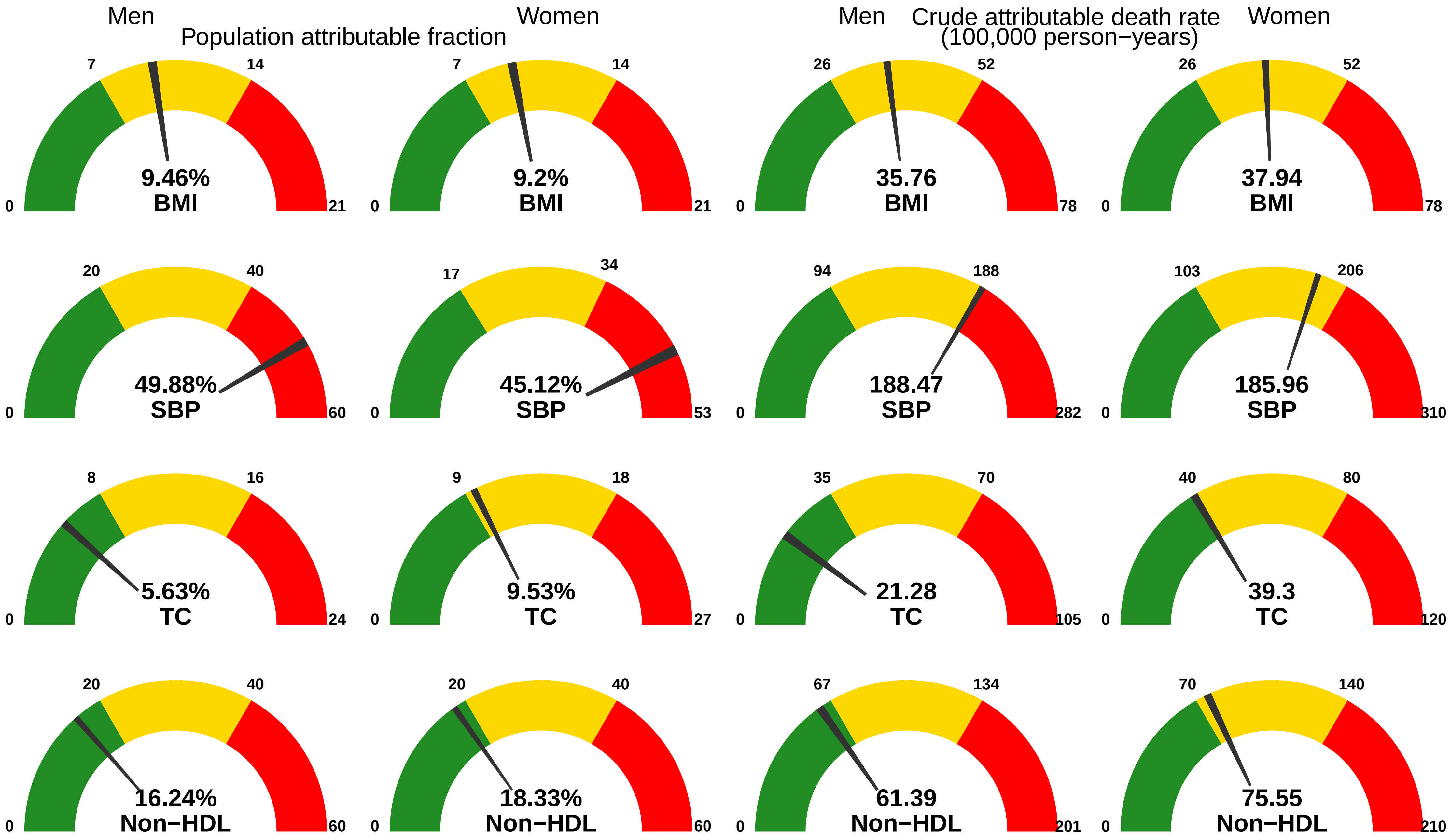

Belize
(Caribbean)

Legend: BMI = body mass index;
SBP = systolic blood pressure;
TC = total cholesterol;
Non−HDL = Non−HDL cholesterol.
Upper values are the largest
observed across countries,
risk factor− and sex−specific.
Sex− and age−specific results
are available through authors.

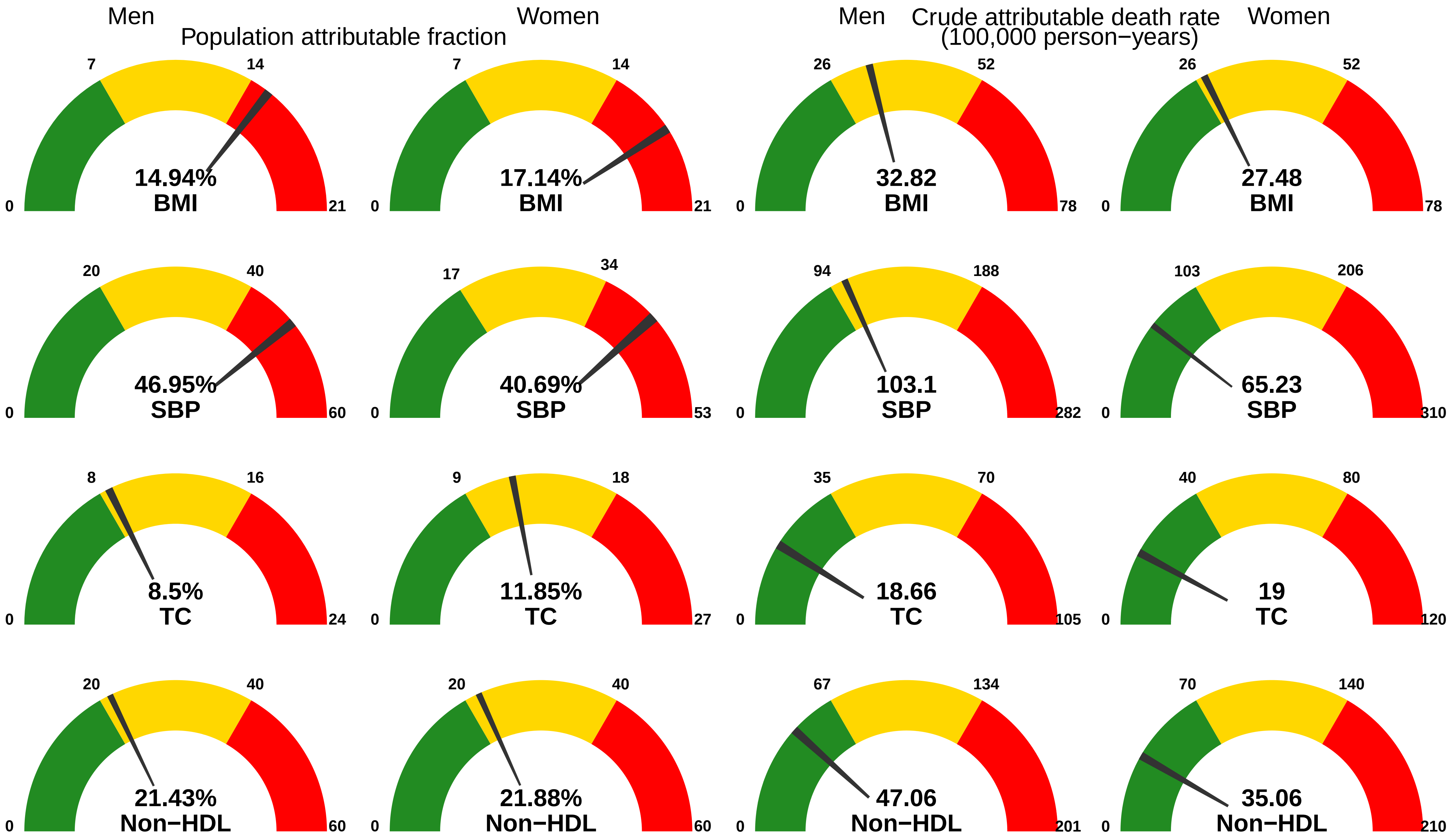

Bermuda

(Caribbean)

Legend: BMI = body mass index;
SBP = systolic blood pressure;
TC = total cholesterol;
Non−HDL = Non−HDL cholesterol.
Upper values are the largest
observed across countries,
risk factor− and sex−specific.
Sex− and age−specific results
are available through authors.

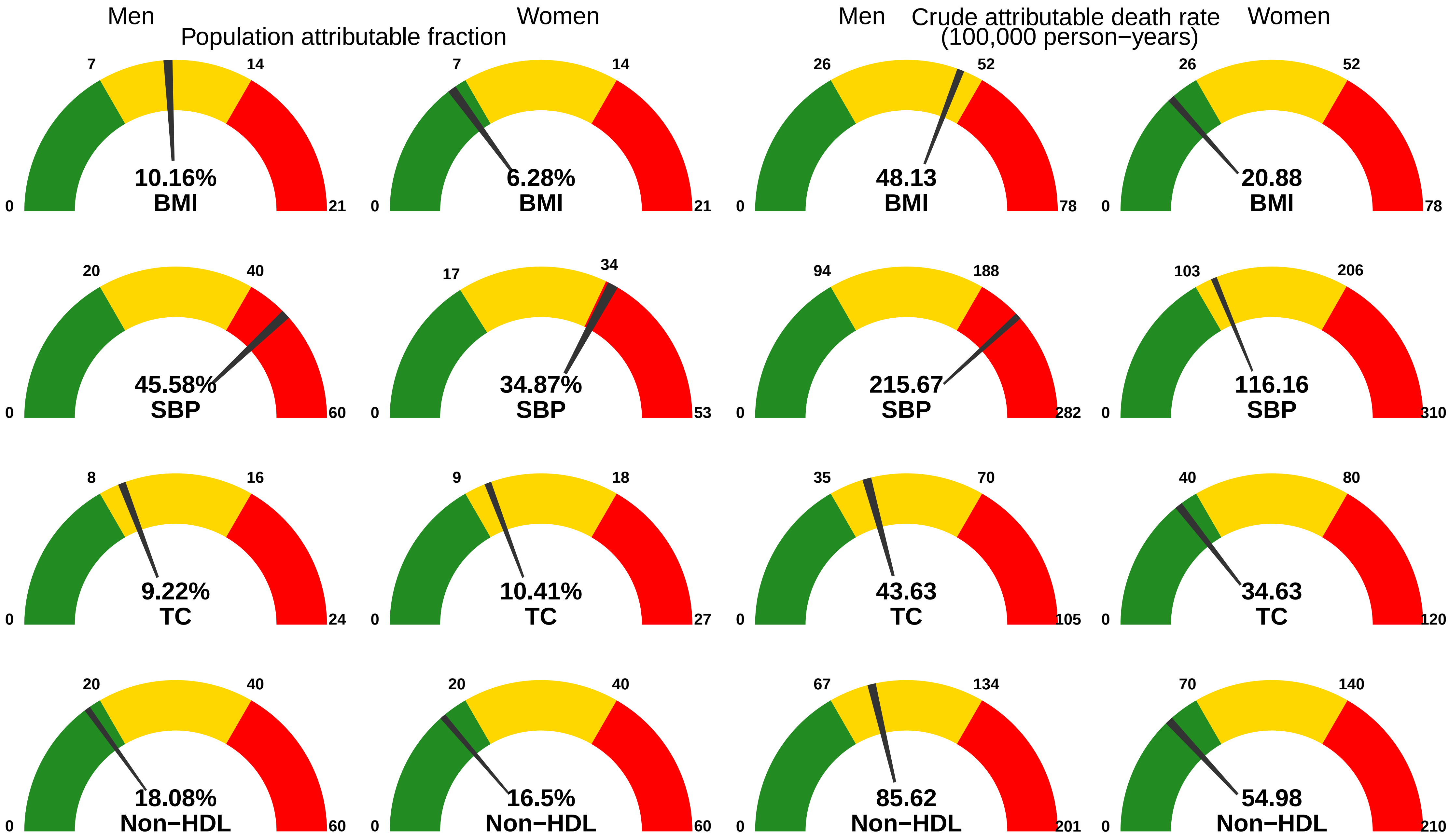

Bolivia

(Andean Latin America)

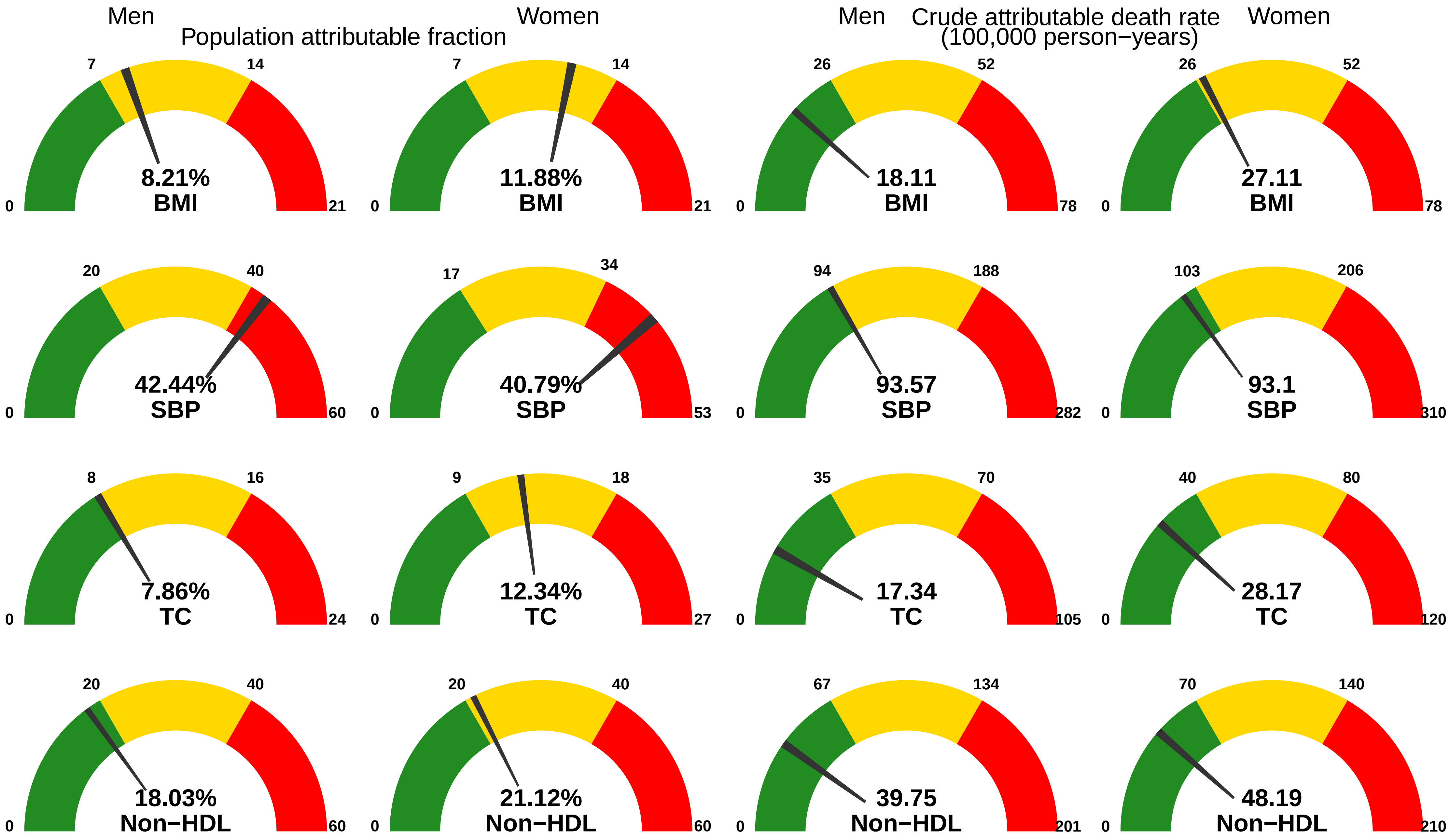

Brazil

(Southern and Tropical Latin America)

Legend: BMI = body mass index;
SBP = systolic blood pressure;
TC = total cholesterol;
Non−HDL = Non−HDL cholesterol.
Upper values are the largest
observed across countries,
risk factor− and sex−specific.
Sex− and age−specific results
are available through authors.

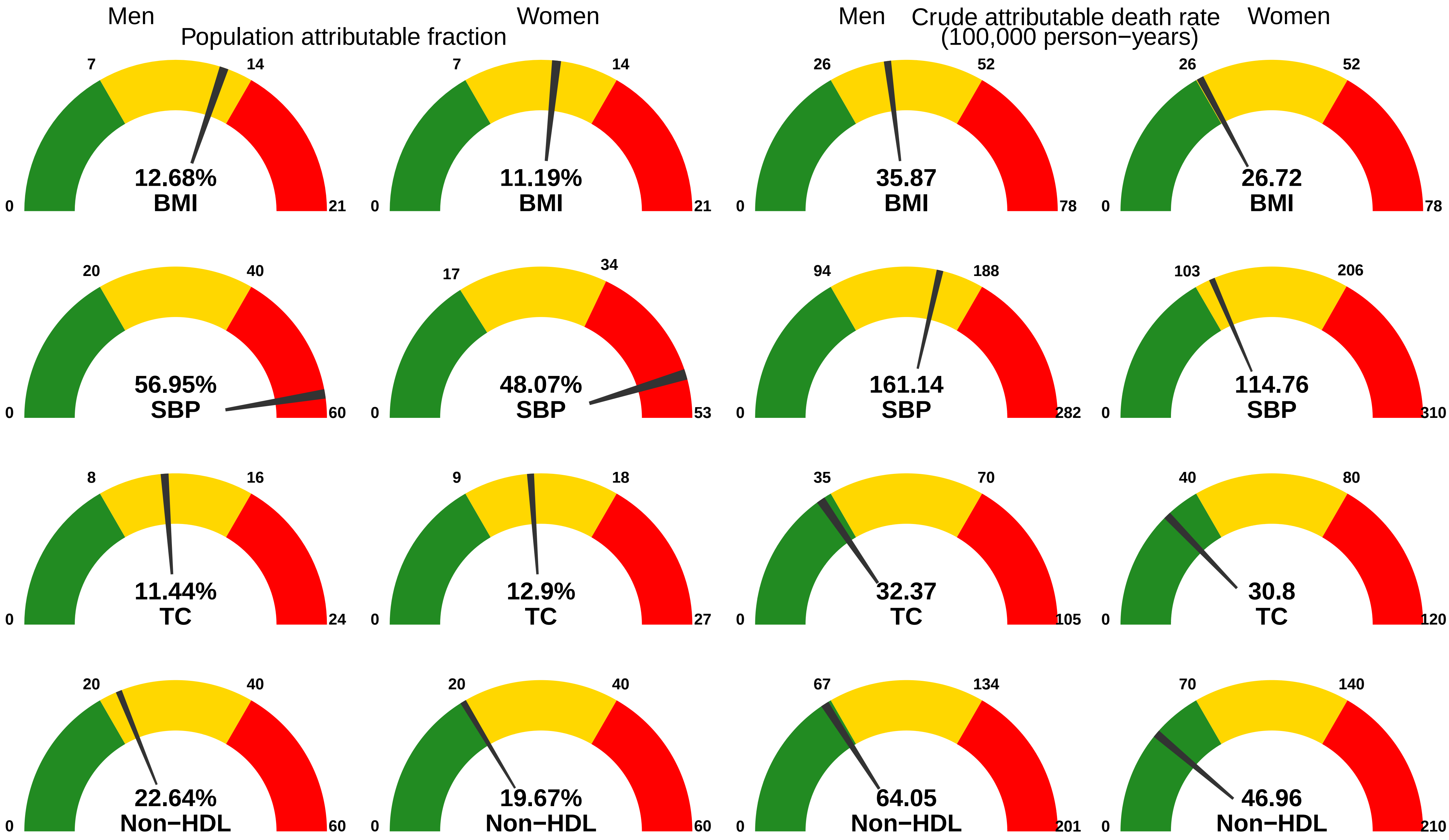

Chile

(Southern and Tropical Latin America)

Legend: BMI = body mass index;
SBP = systolic blood pressure;
TC = total cholesterol;
Non−HDL = Non−HDL cholesterol.
Upper values are the largest
observed across countries,
risk factor− and sex−specific.
Sex− and age−specific results
are available through authors.

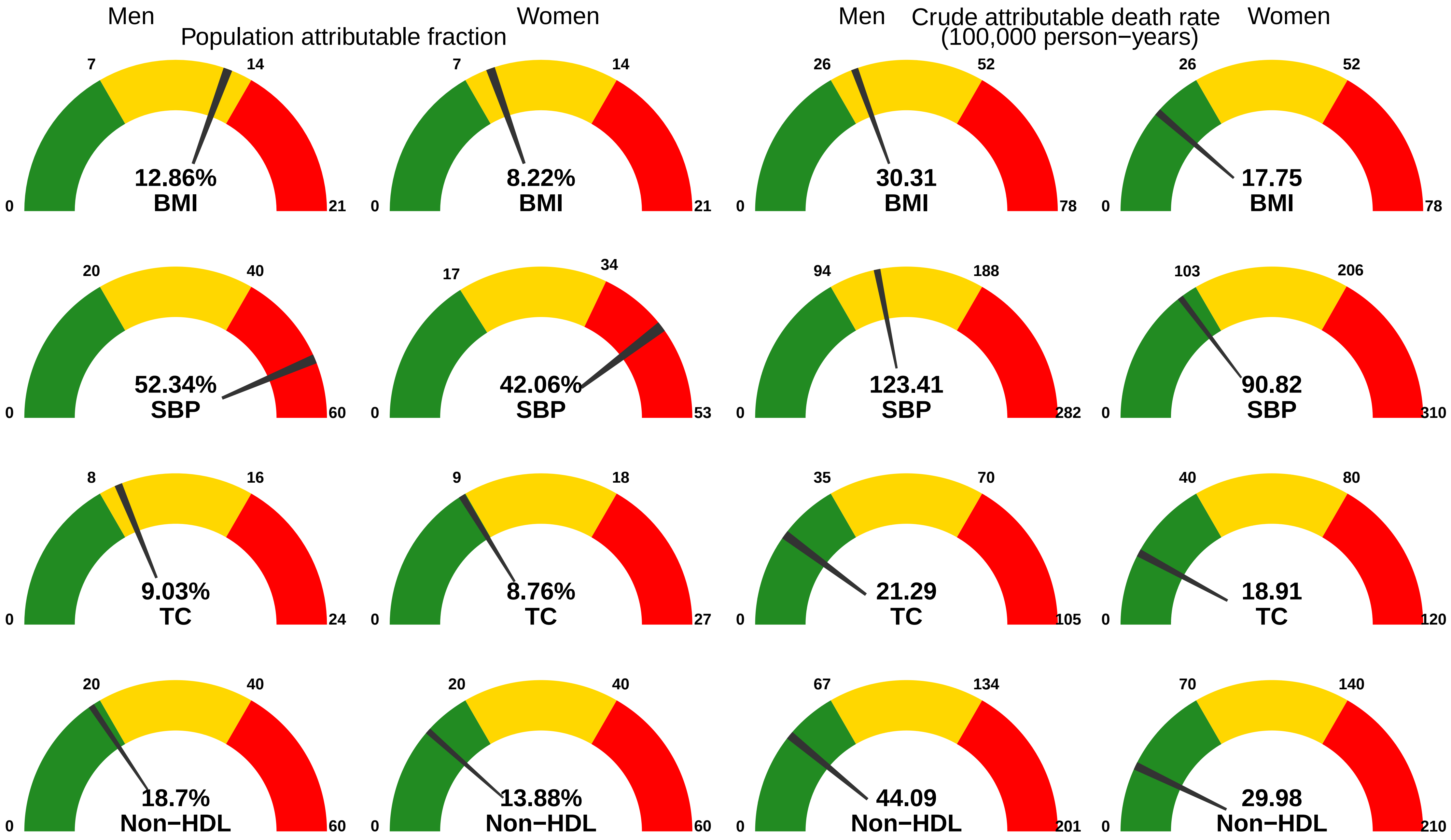

Colombia

(Central Latin America)

Legend: BMI = body mass index;
SBP = systolic blood pressure;
TC = total cholesterol;
Non−HDL = Non−HDL cholesterol.
Upper values are the largest
observed across countries,
risk factor− and sex−specific.
Sex− and age−specific results
are available through authors.

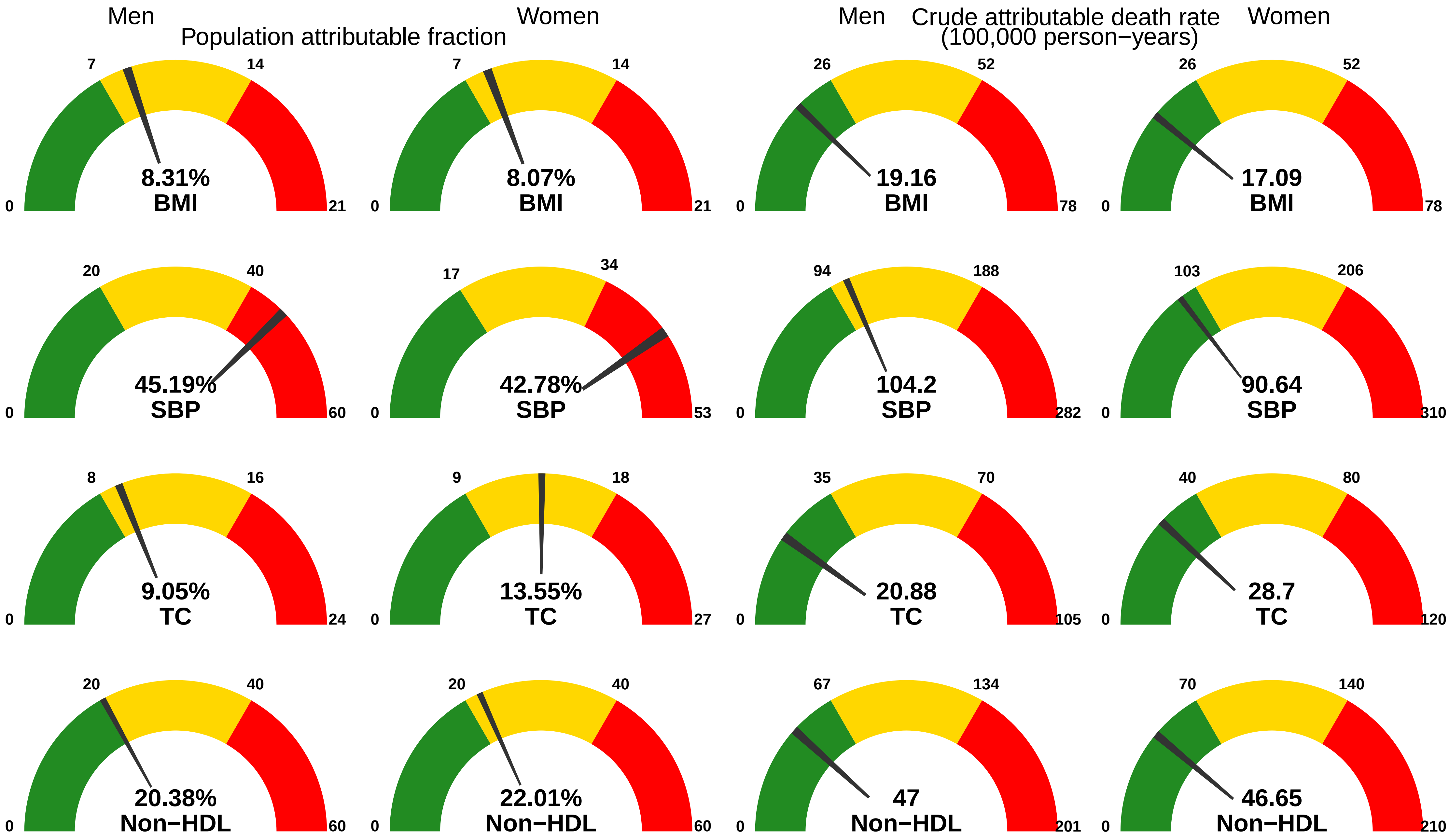

Costa Rica

(Central Latin America)

Legend: BMI = body mass index;
SBP = systolic blood pressure;
TC = total cholesterol;
Non−HDL = Non−HDL cholesterol.
Upper values are the largest observed across countries, risk factor− and sex−specific.
Sex− and age−specific results are available through authors.

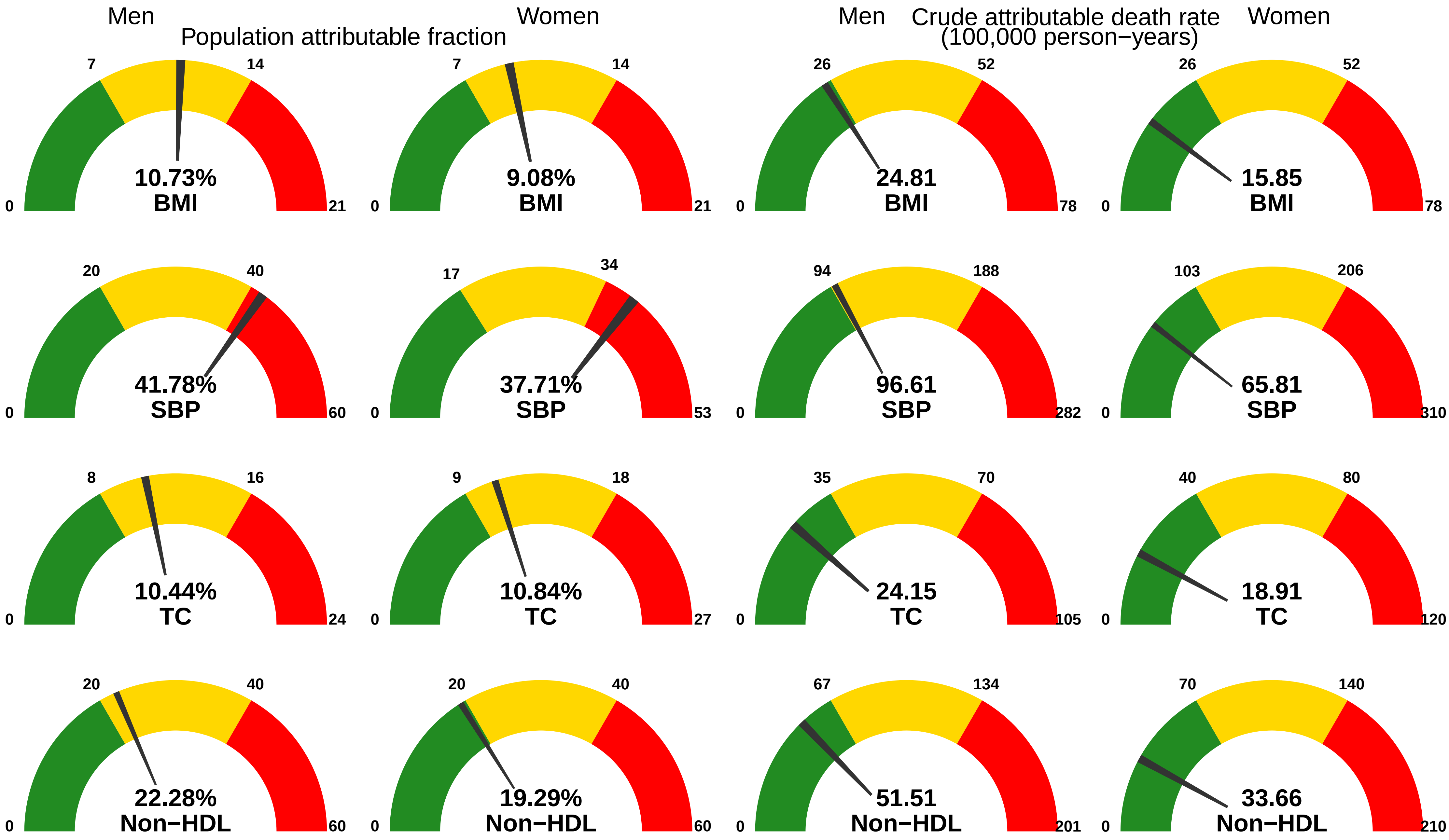

Cuba
(Caribbean)

Legend: BMI = body mass index;
SBP = systolic blood pressure;
TC = total cholesterol;
Non−HDL = Non−HDL cholesterol.
Upper values are the largest
observed across countries,
risk factor− and sex−specific.
Sex− and age−specific results
are available through authors.

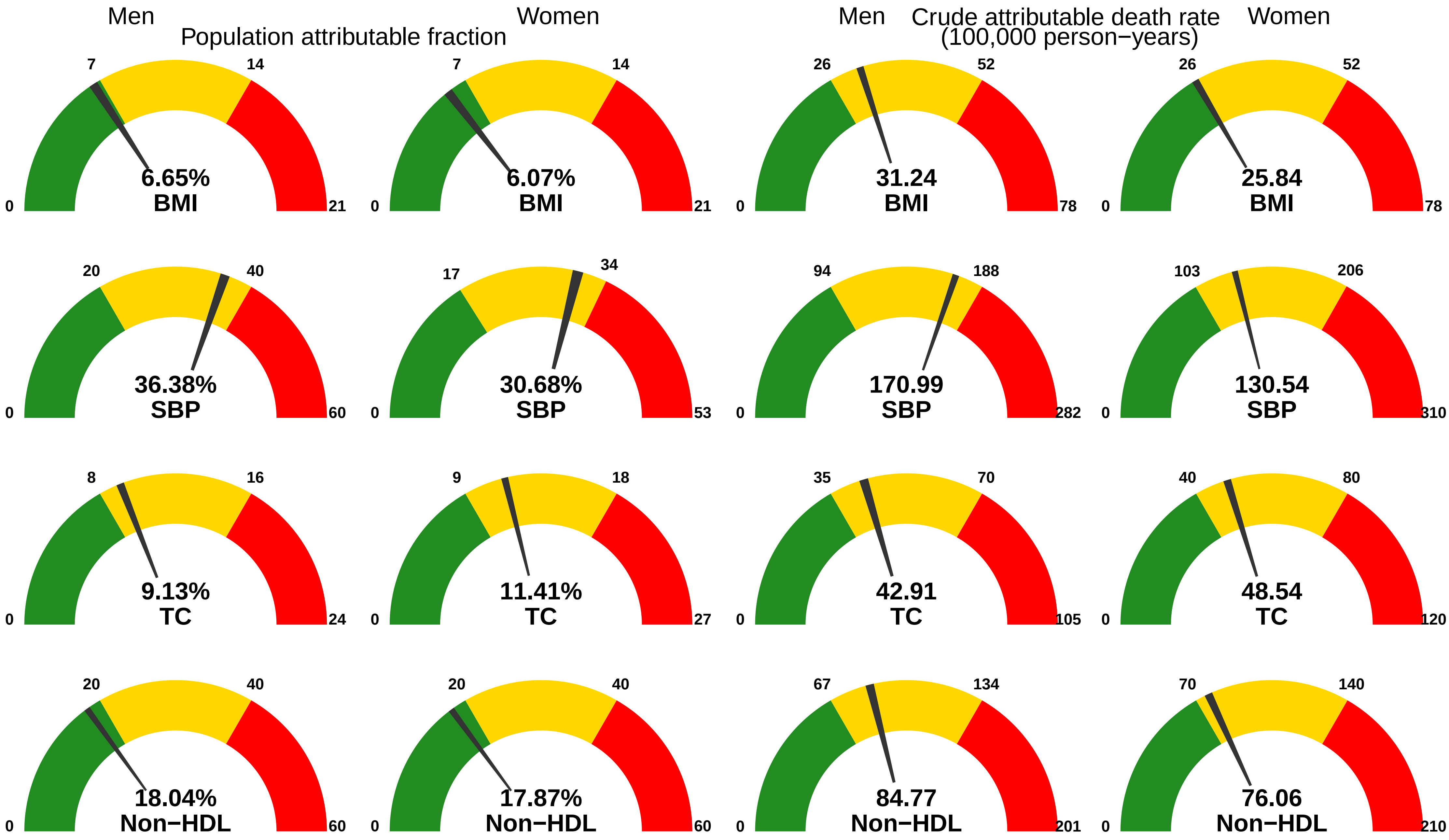

Dominica

(Caribbean)

Legend: BMI = body mass index;
SBP = systolic blood pressure;
TC = total cholesterol;
Non−HDL = Non−HDL cholesterol.
Upper values are the largest observed across countries, risk factor− and sex−specific.
Sex− and age−specific results are available through authors.

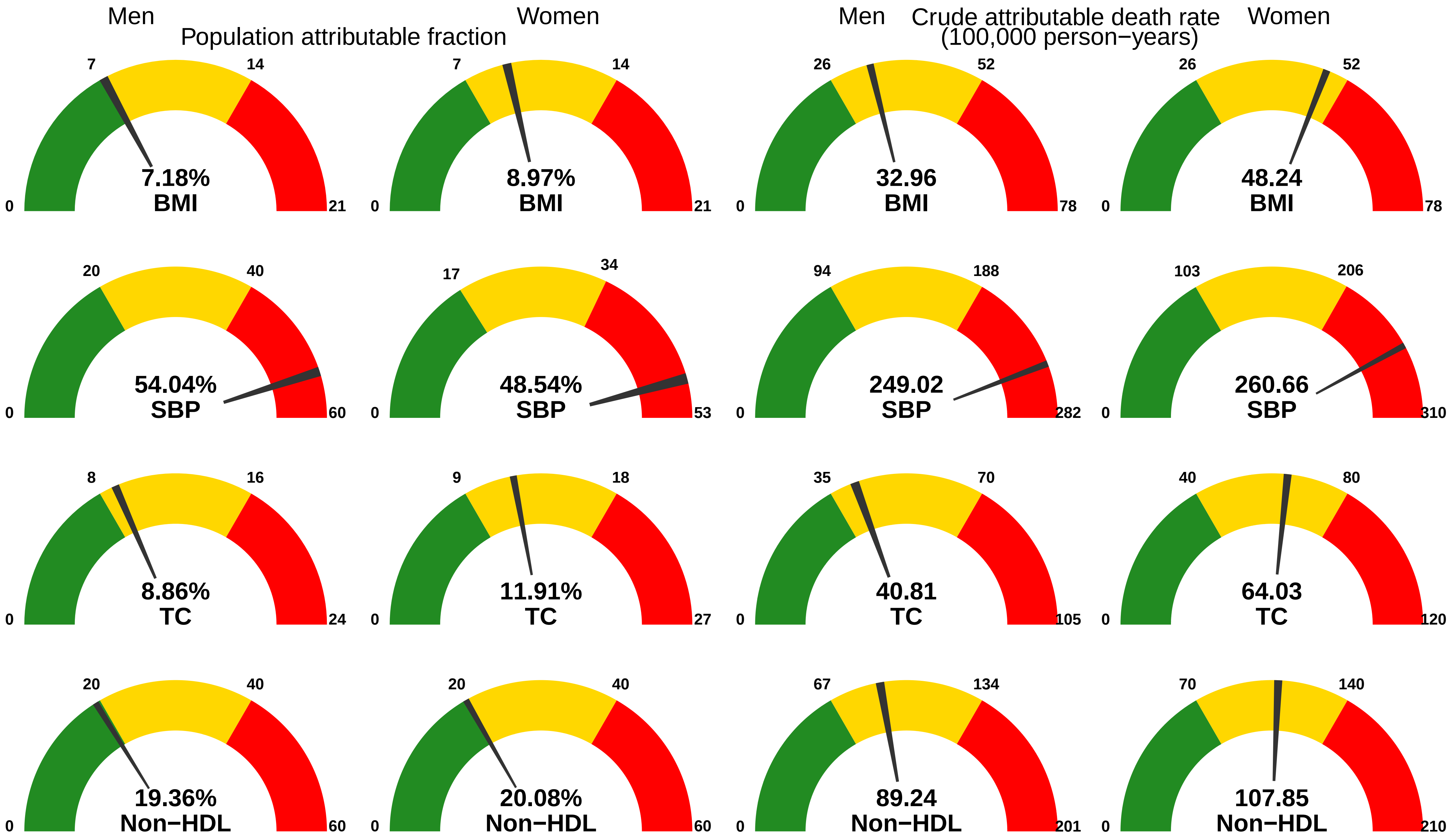

Dominican Republic

(Caribbean)

Legend: BMI = body mass index;
SBP = systolic blood pressure;
TC = total cholesterol;
Non−HDL = Non−HDL cholesterol.
Upper values are the largest observed across countries, risk factor− and sex−specific.
Sex− and age−specific results are available through authors.

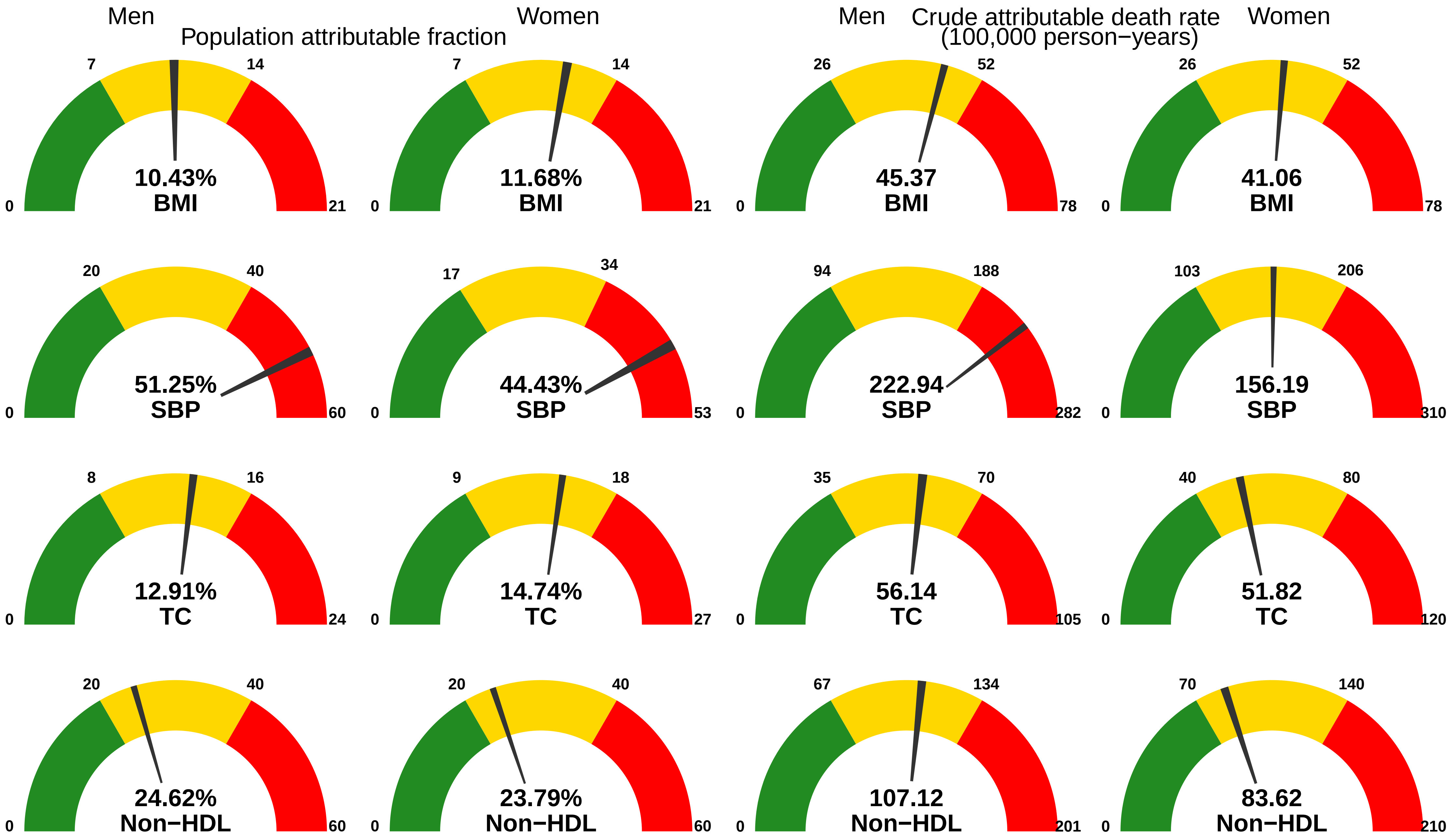

Ecuador

(Andean Latin America)

Legend: BMI = body mass index;
SBP = systolic blood pressure;
TC = total cholesterol;
Non−HDL = Non−HDL cholesterol.
Upper values are the largest
observed across countries,
risk factor− and sex−specific.
Sex− and age−specific results
are available through authors.

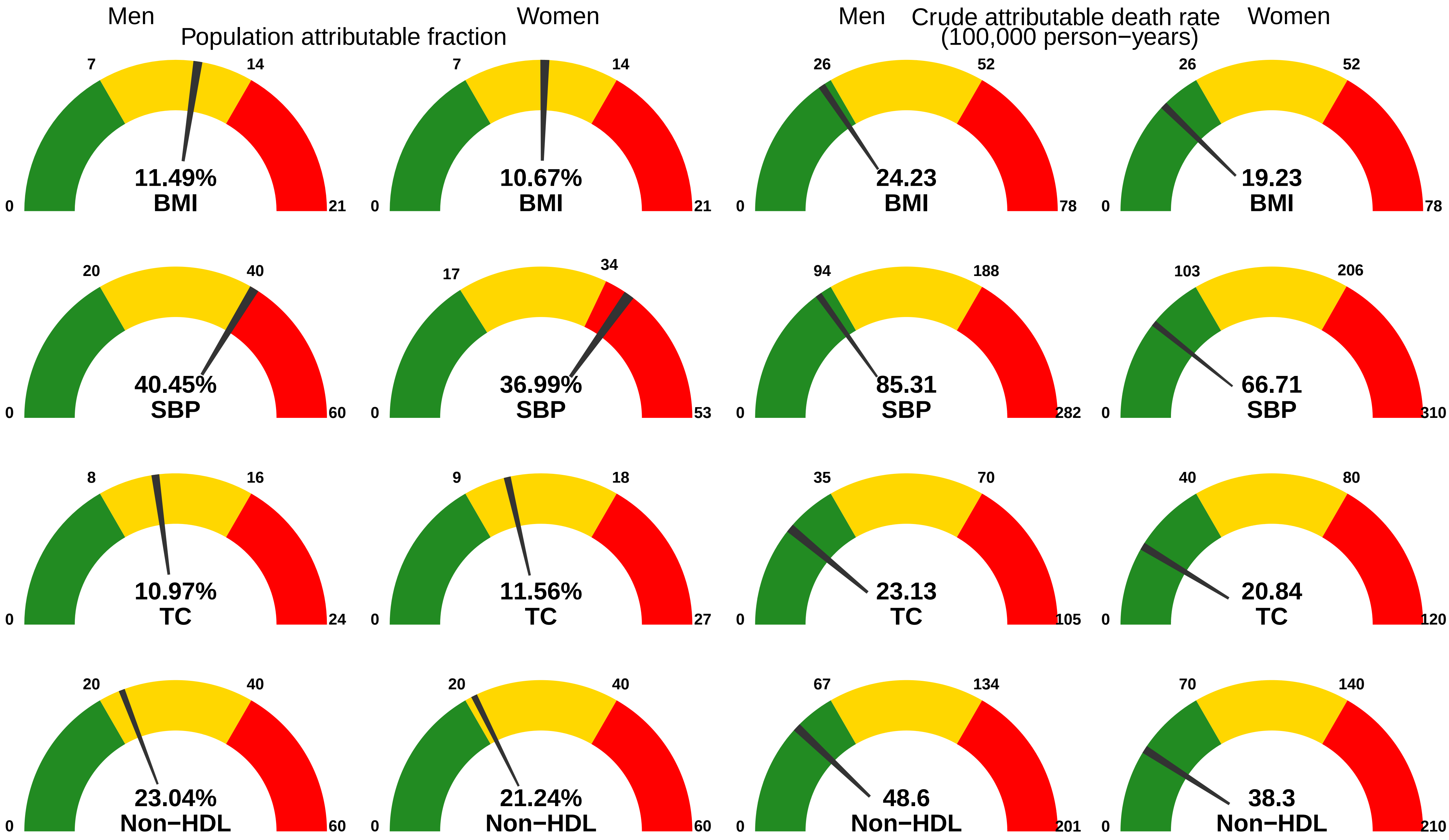

El Salvador

(Central Latin America)

Legend: BMI = body mass index;
SBP = systolic blood pressure;
TC = total cholesterol;
Non−HDL = Non−HDL cholesterol.
Upper values are the largest observed across countries, risk factor− and sex−specific.
Sex− and age−specific results are available through authors.

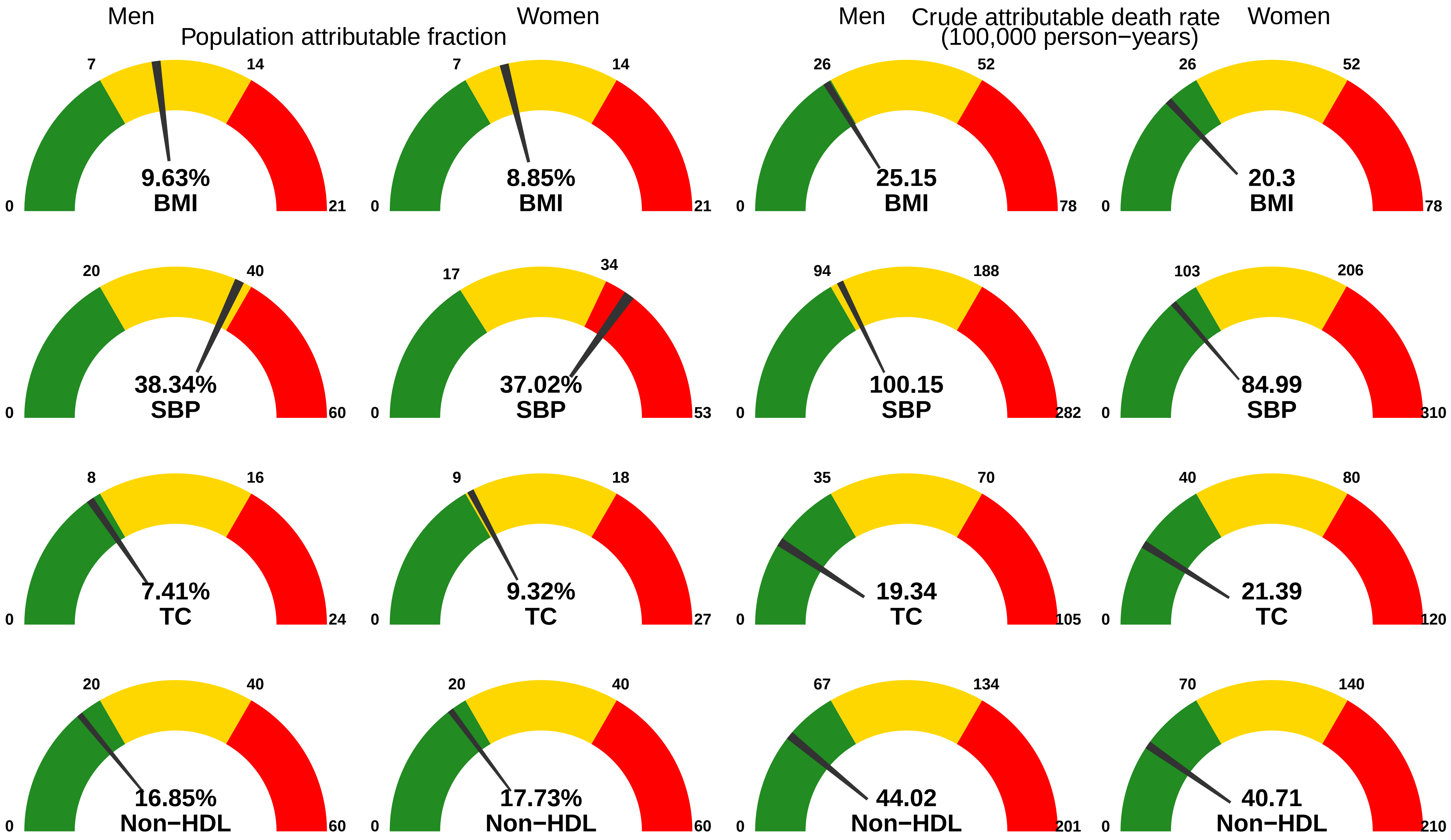

Grenada
(Caribbean)

Legend: BMI = body mass index;
SBP = systolic blood pressure;
TC = total cholesterol;
Non−HDL = Non−HDL cholesterol.
Upper values are the largest
observed across countries,
risk factor− and sex−specific.
Sex− and age−specific results
are available through authors.

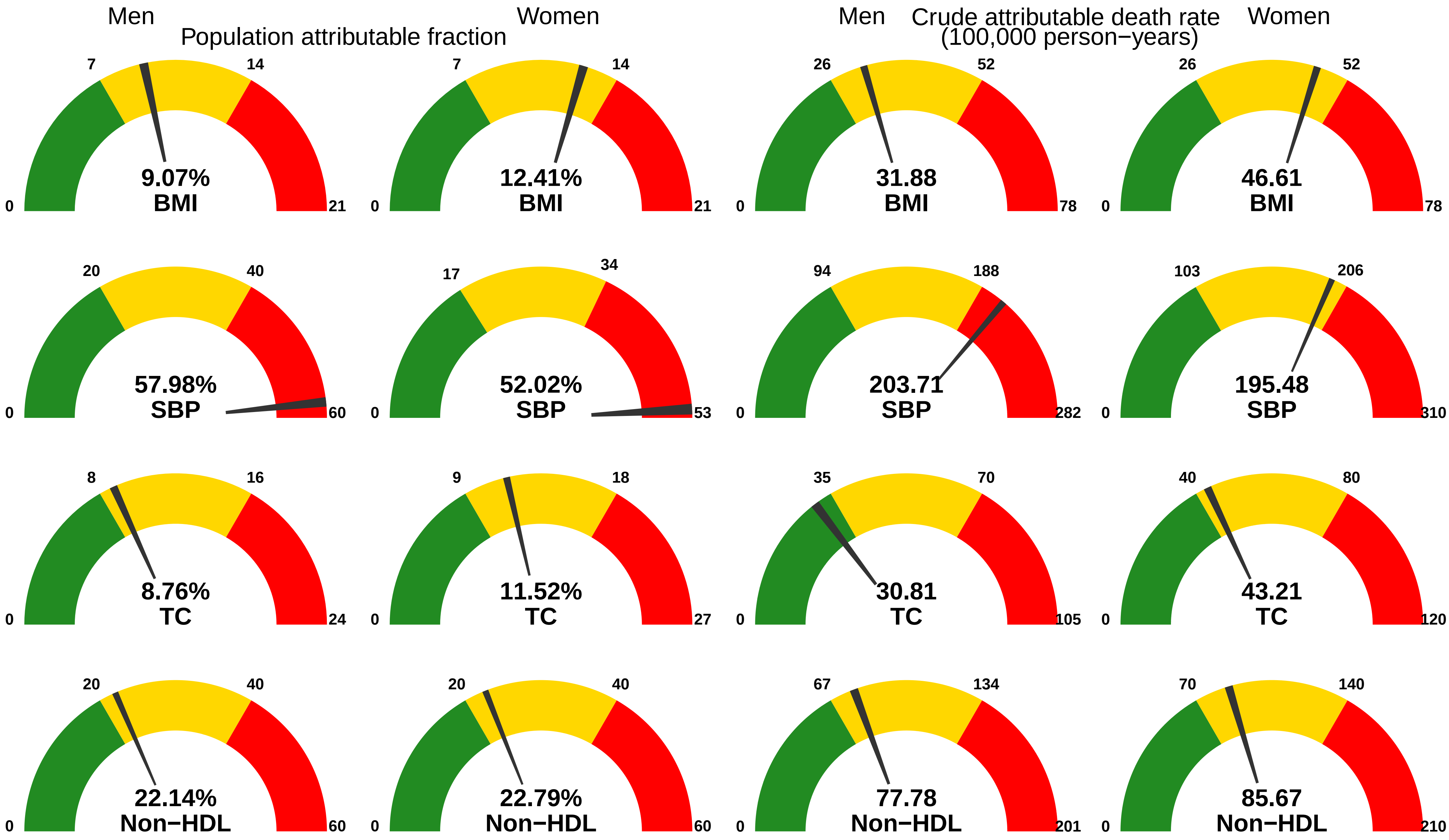

Guatemala

(Central Latin America)

Legend: BMI = body mass index;
SBP = systolic blood pressure;
TC = total cholesterol;
Non−HDL = Non−HDL cholesterol.
Upper values are the largest observed across countries, risk factor− and sex−specific.
Sex− and age−specific results are available through authors.

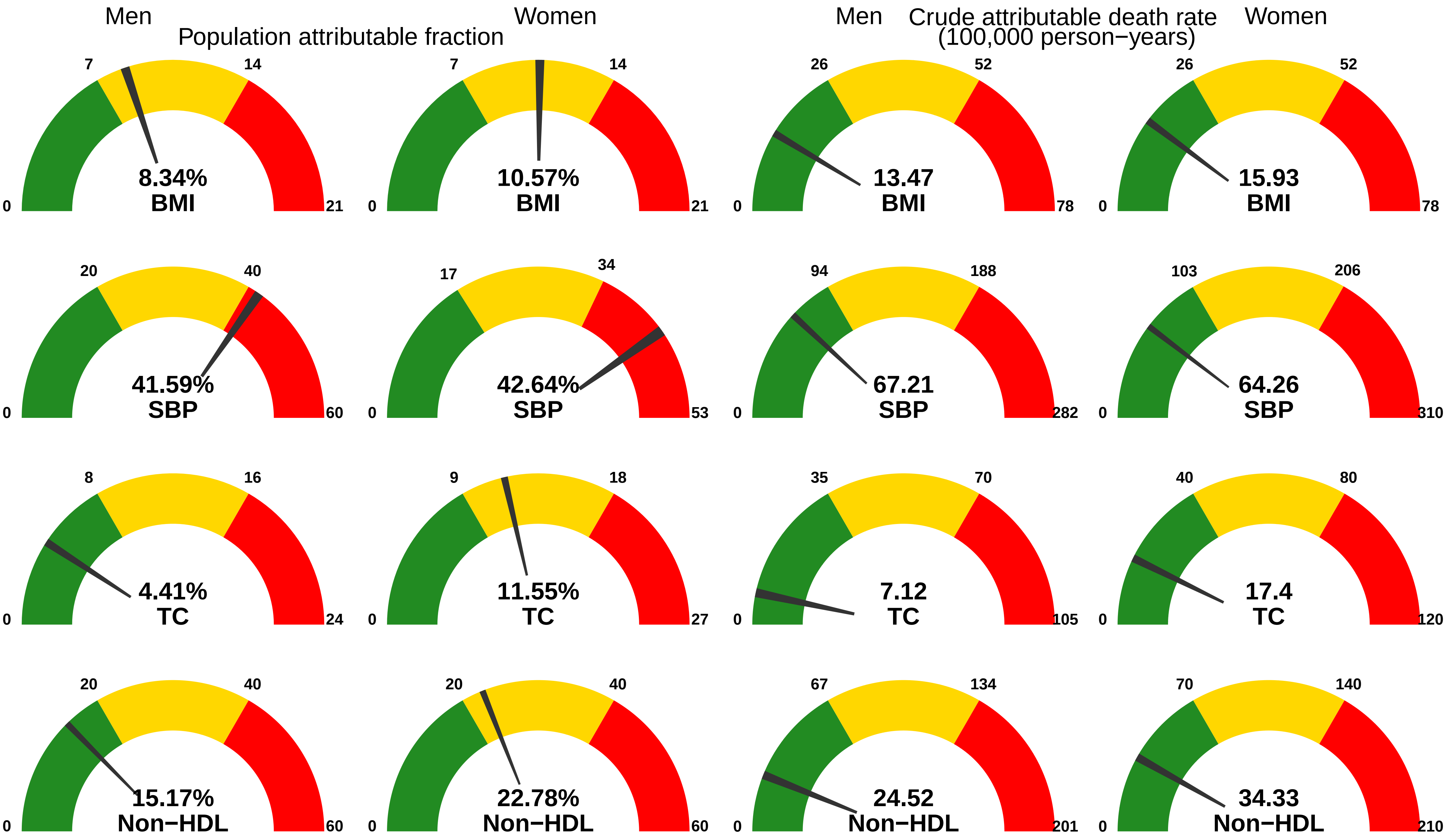

Guyana
(Caribbean)

Legend: BMI = body mass index;
SBP = systolic blood pressure;
TC = total cholesterol;
Non−HDL = Non−HDL cholesterol.
Upper values are the largest
observed across countries,
risk factor− and sex−specific.
Sex− and age−specific results
are available through authors.

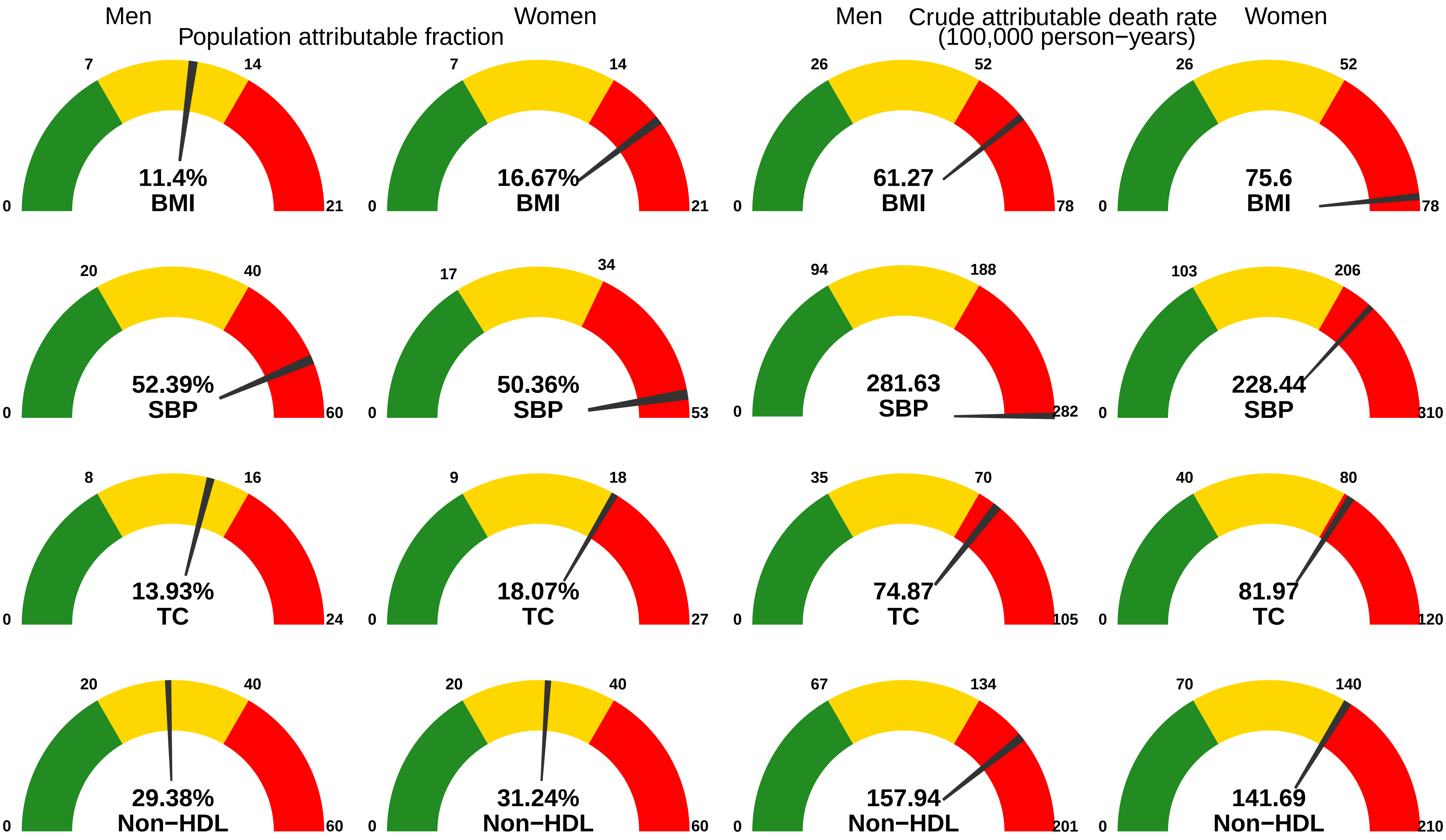

Haiti

(Caribbean)

Legend: BMI = body mass index;
SBP = systolic blood pressure;
TC = total cholesterol;
Non−HDL = Non−HDL cholesterol.
Upper values are the largest observed across countries, risk factor− and sex−specific.
Sex− and age−specific results are available through authors.

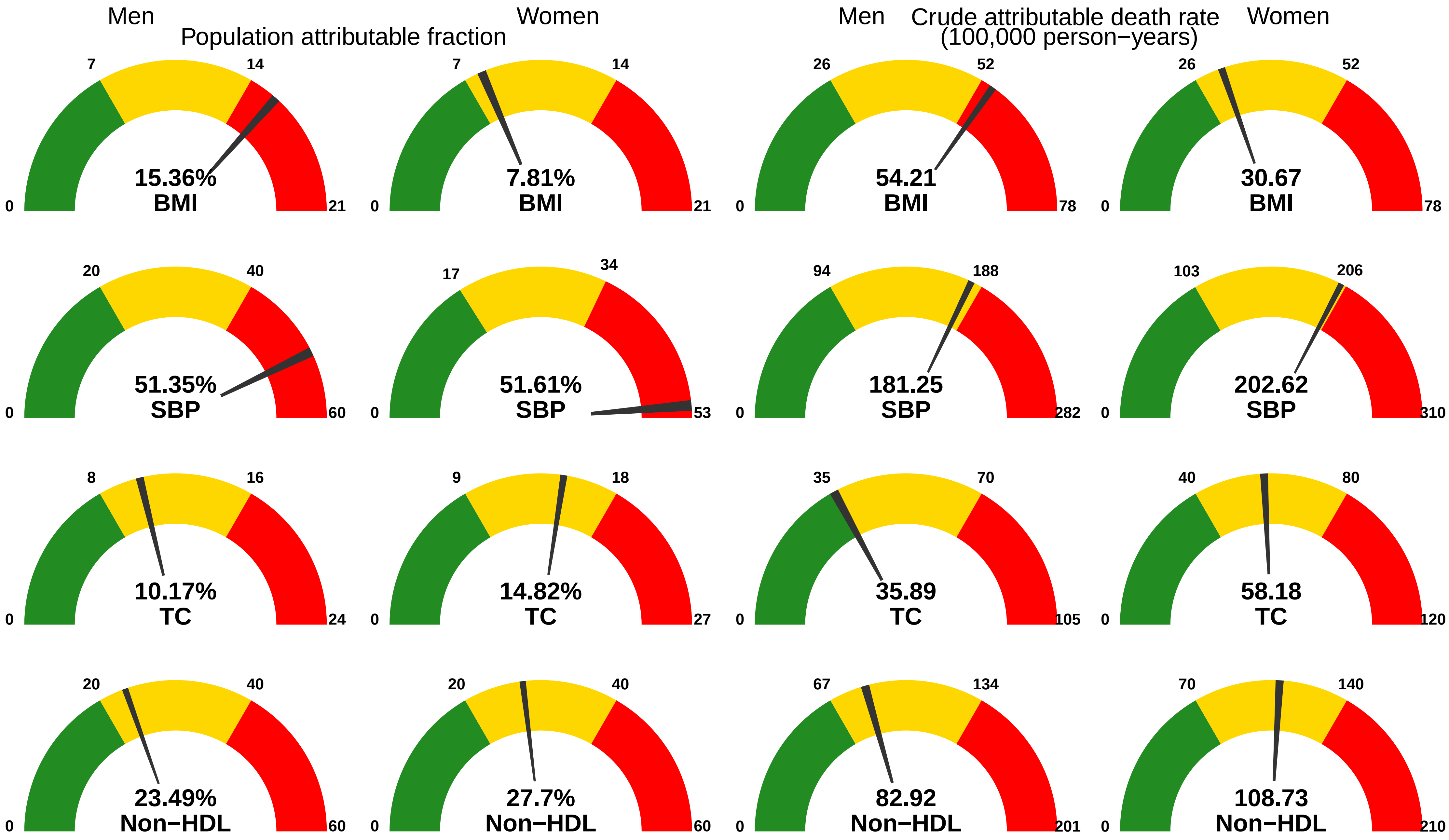

Honduras
(Central Latin America)

Legend: BMI = body mass index;
SBP = systolic blood pressure;
TC = total cholesterol;
Non−HDL = Non−HDL cholesterol.
Upper values are the largest
observed across countries,
risk factor− and sex−specific.
Sex− and age−specific results
are available through authors.

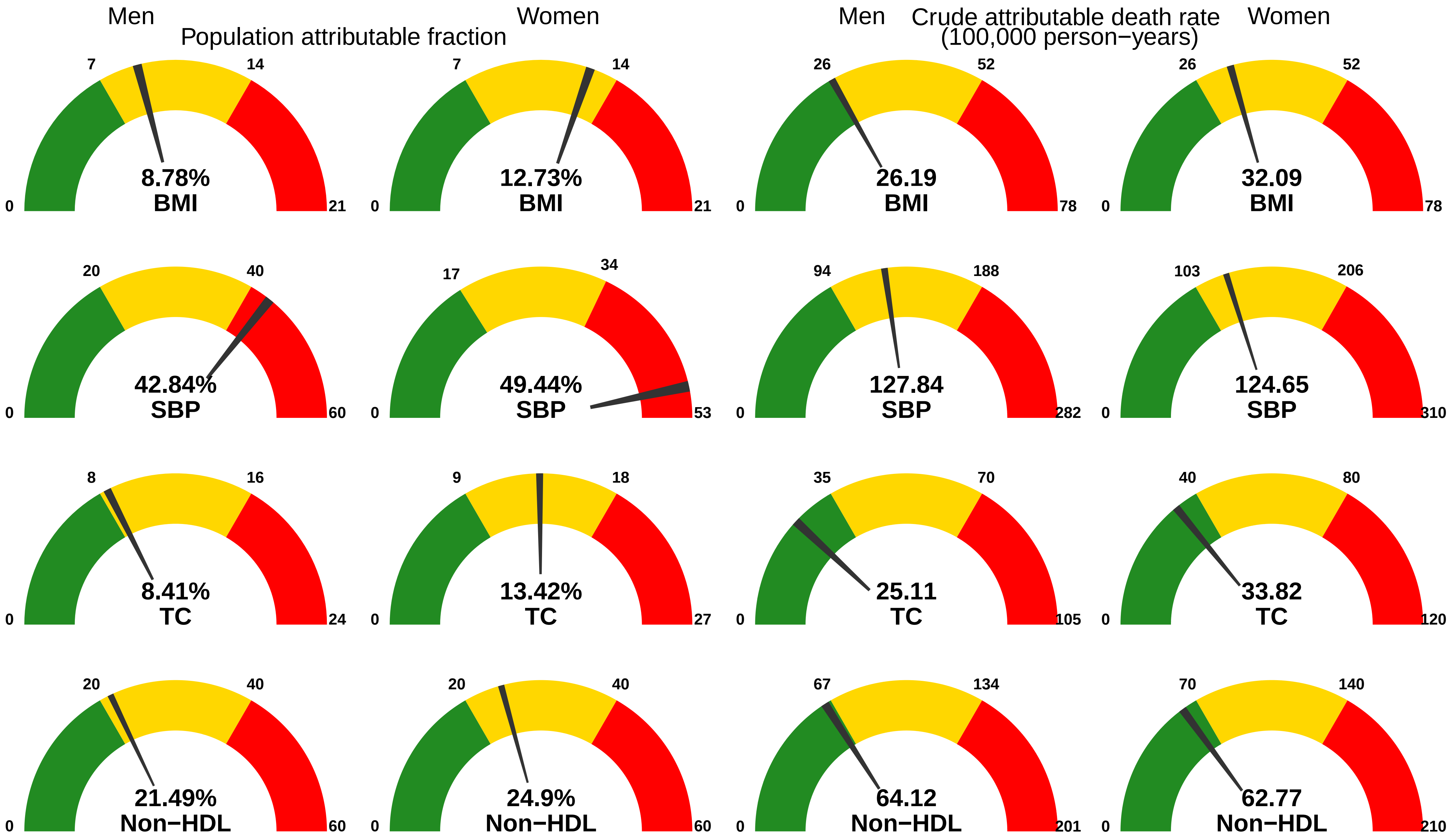

Jamaica

(Caribbean)

Legend: BMI = body mass index;
SBP = systolic blood pressure;
TC = total cholesterol;
Non−HDL = Non−HDL cholesterol.
Upper values are the largest observed across countries, risk factor− and sex−specific.
Sex− and age−specific results are available through authors.

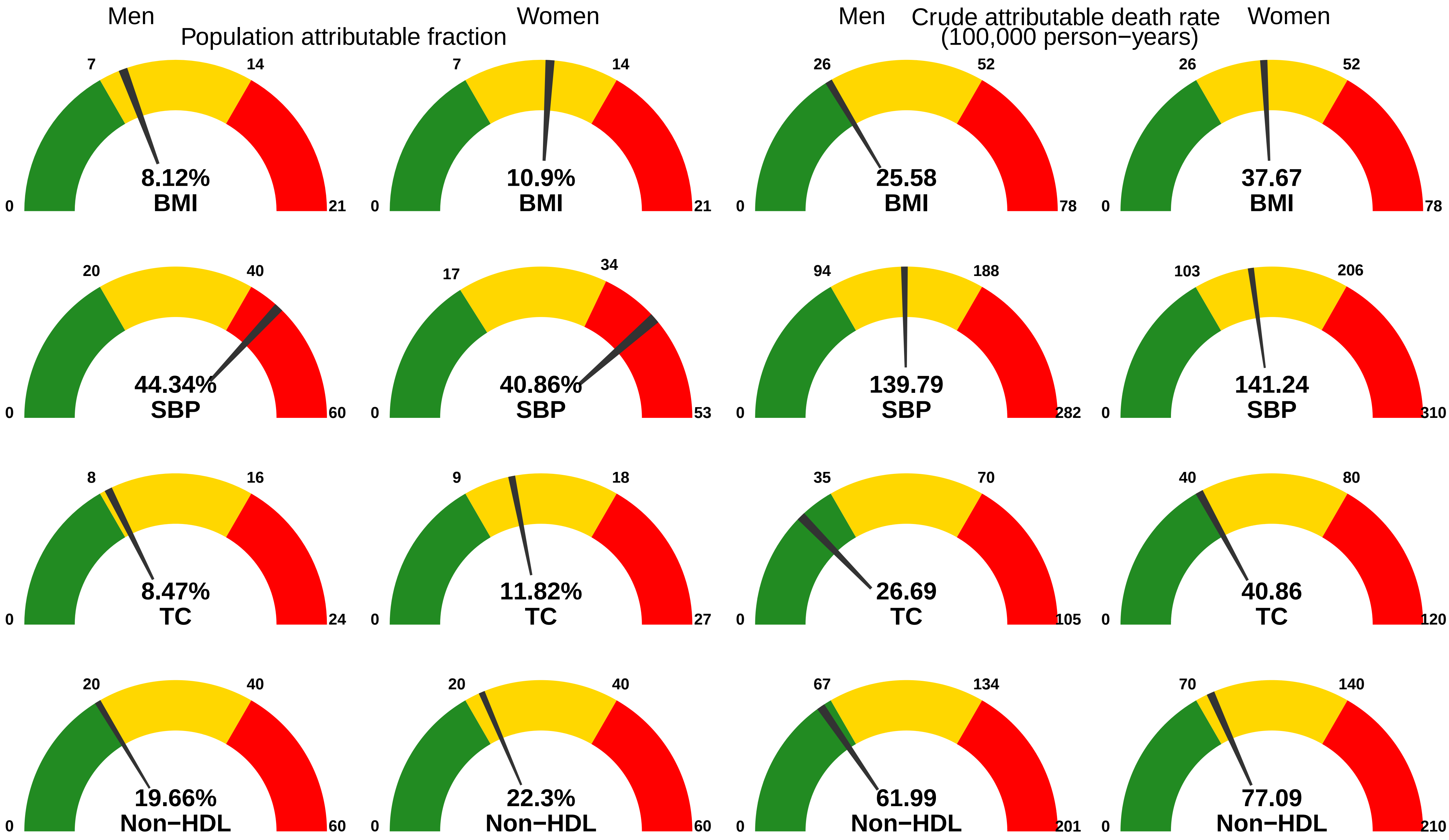

Mexico

(Central Latin America)

Legend: BMI = body mass index;
SBP = systolic blood pressure;
TC = total cholesterol;
Non−HDL = Non−HDL cholesterol.
Upper values are the largest observed across countries, risk factor− and sex−specific.
Sex− and age−specific results are available through authors.

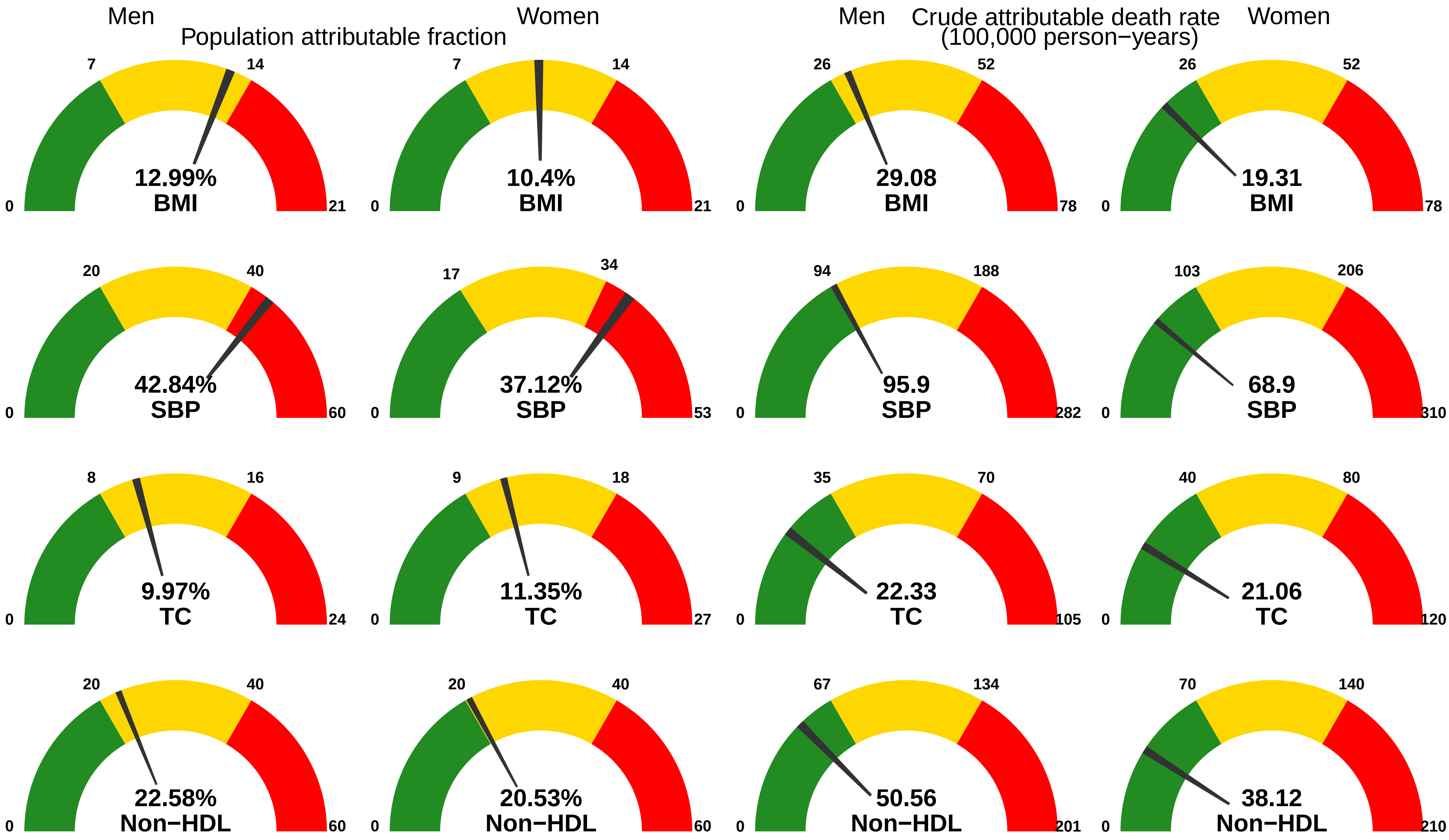

Nicaragua
(Central Latin America)

Legend: BMI = body mass index;
SBP = systolic blood pressure;
TC = total cholesterol;
Non−HDL = Non−HDL cholesterol.
Upper values are the largest
observed across countries,
risk factor− and sex−specific.
Sex− and age−specific results
are available through authors.

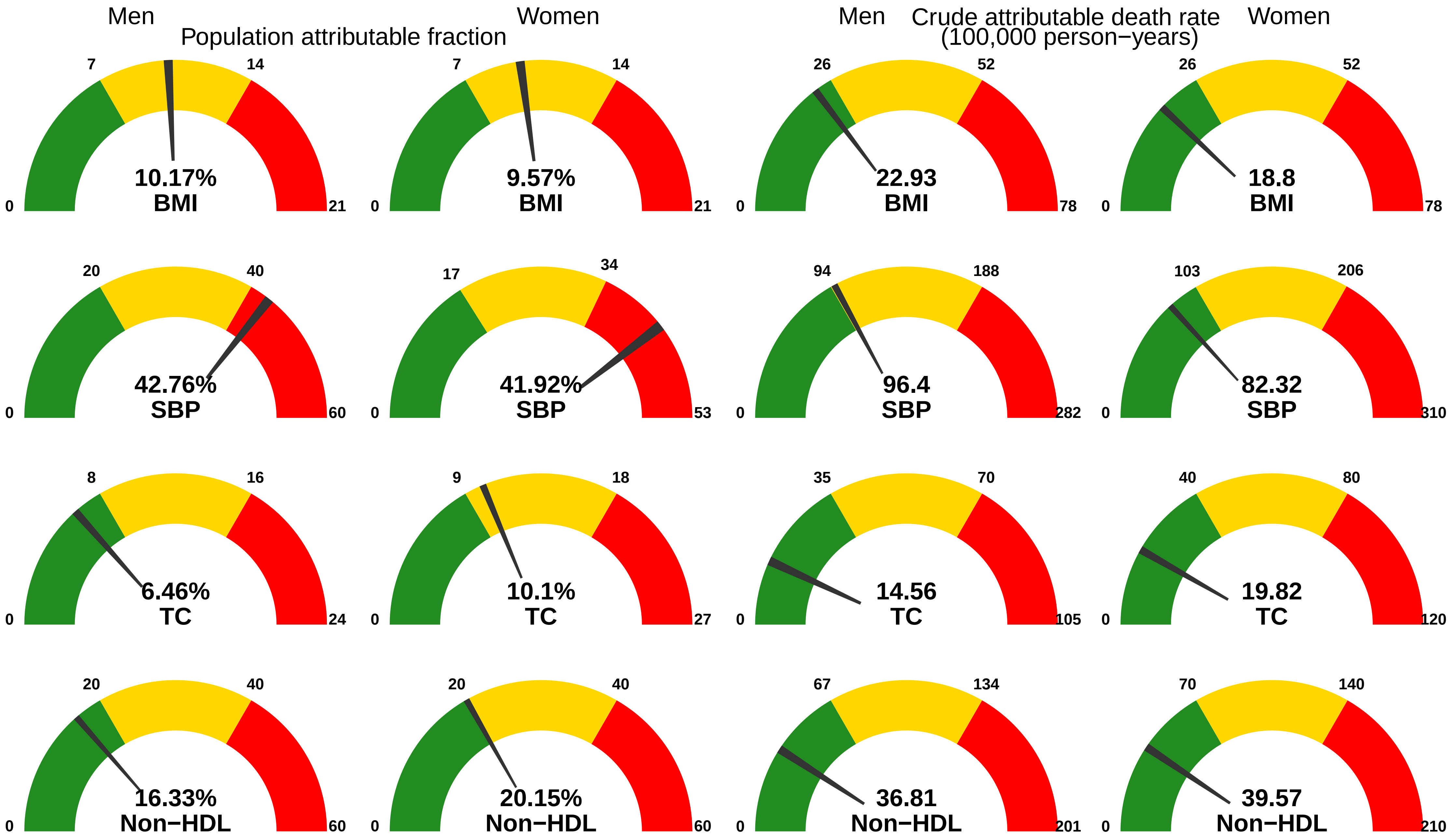

Panama

(Central Latin America)

Legend: BMI = body mass index;
SBP = systolic blood pressure;
TC = total cholesterol;
Non−HDL = Non−HDL cholesterol.
Upper values are the largest
observed across countries,
risk factor− and sex−specific.
Sex− and age−specific results
are available through authors.

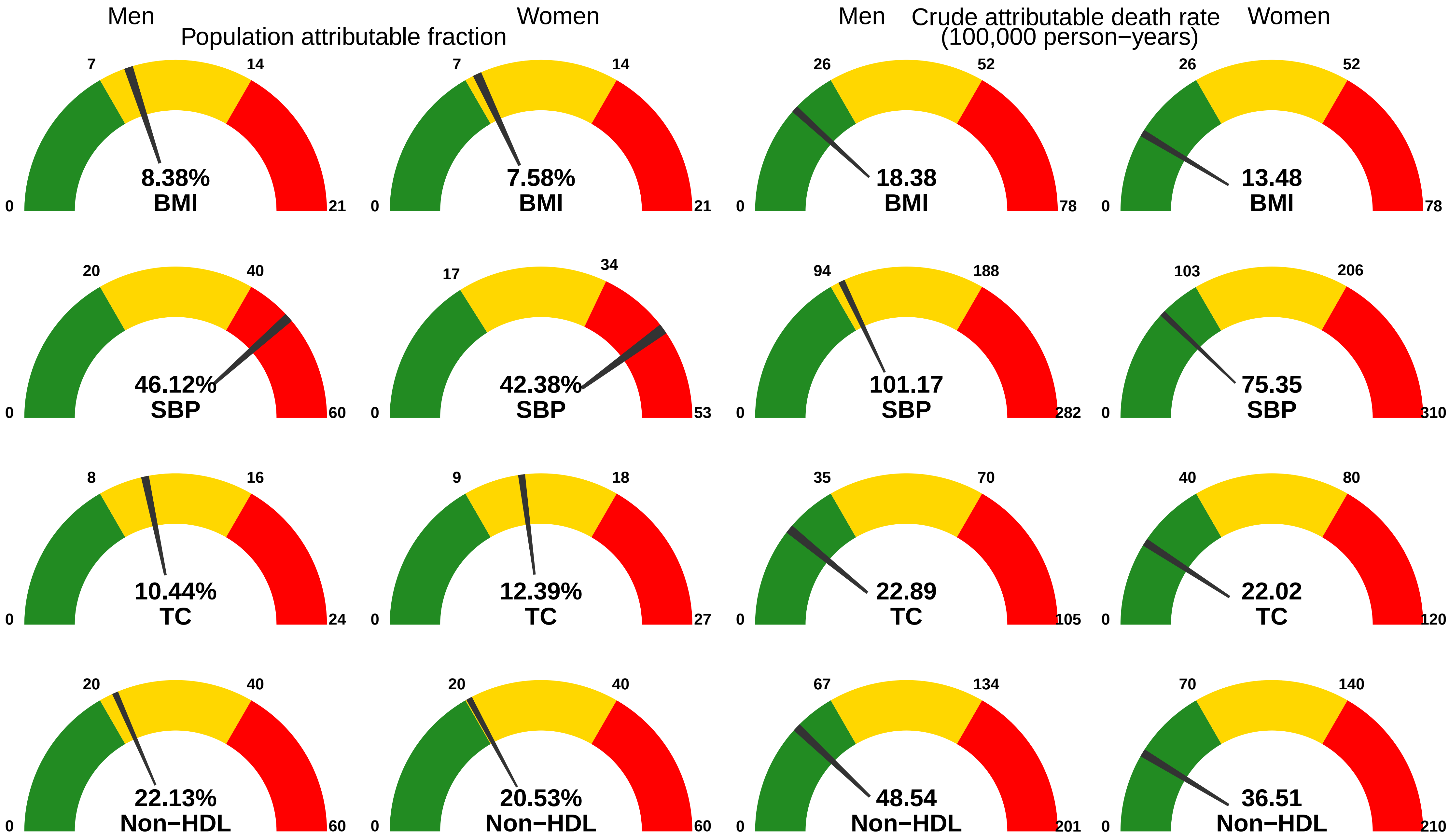

Paraguay

(Southern and Tropical Latin America)

Legend: BMI = body mass index;
SBP = systolic blood pressure;
TC = total cholesterol;
Non−HDL = Non−HDL cholesterol.
Upper values are the largest
observed across countries,
risk factor− and sex−specific.
Sex− and age−specific results
are available through authors.

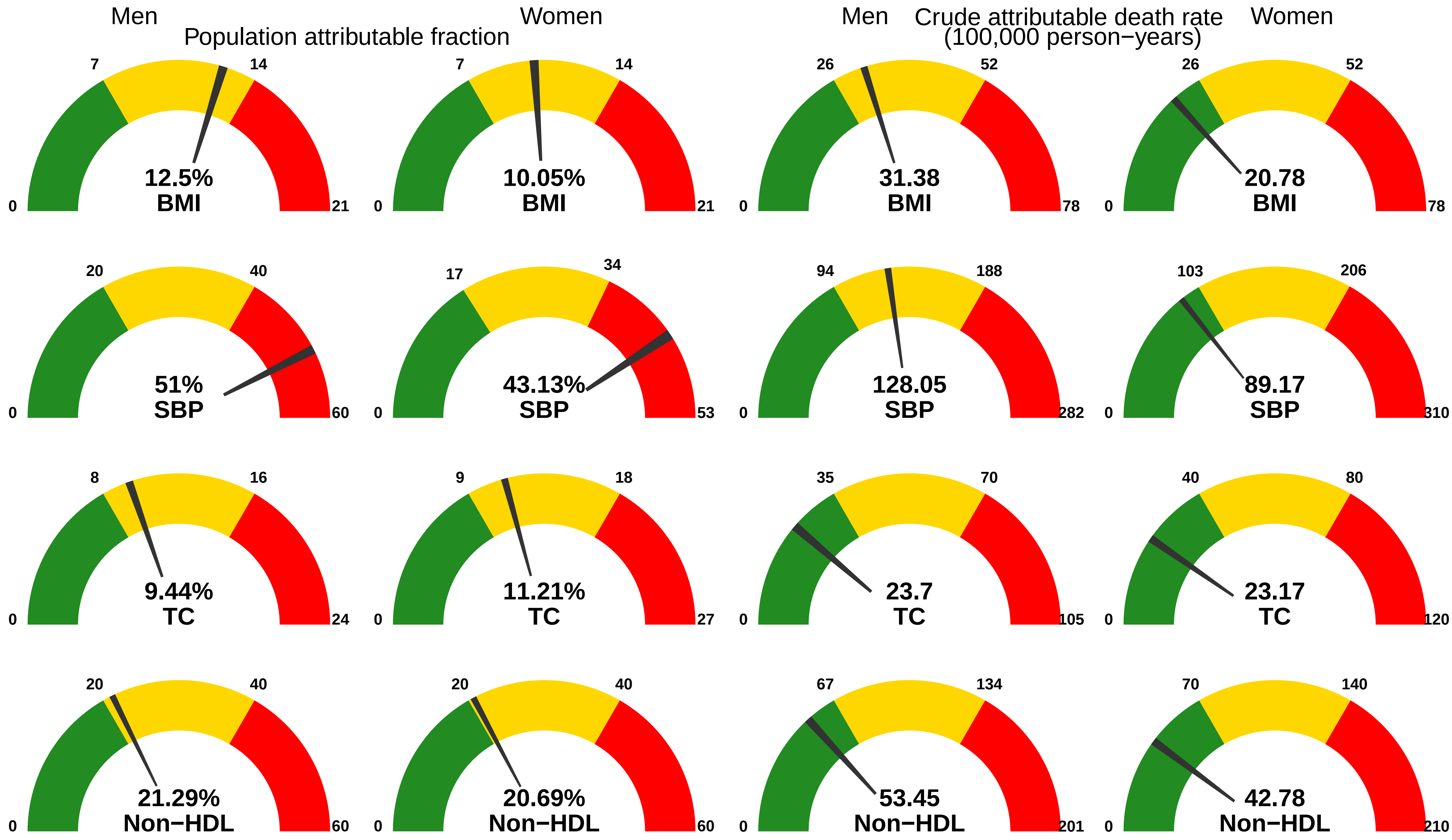

Peru

(Andean Latin America)

Legend: BMI = body mass index;
SBP = systolic blood pressure;
TC = total cholesterol;
Non−HDL = Non−HDL cholesterol.
Upper values are the largest
observed across countries,
risk factor− and sex−specific.
Sex− and age−specific results
are available through authors.

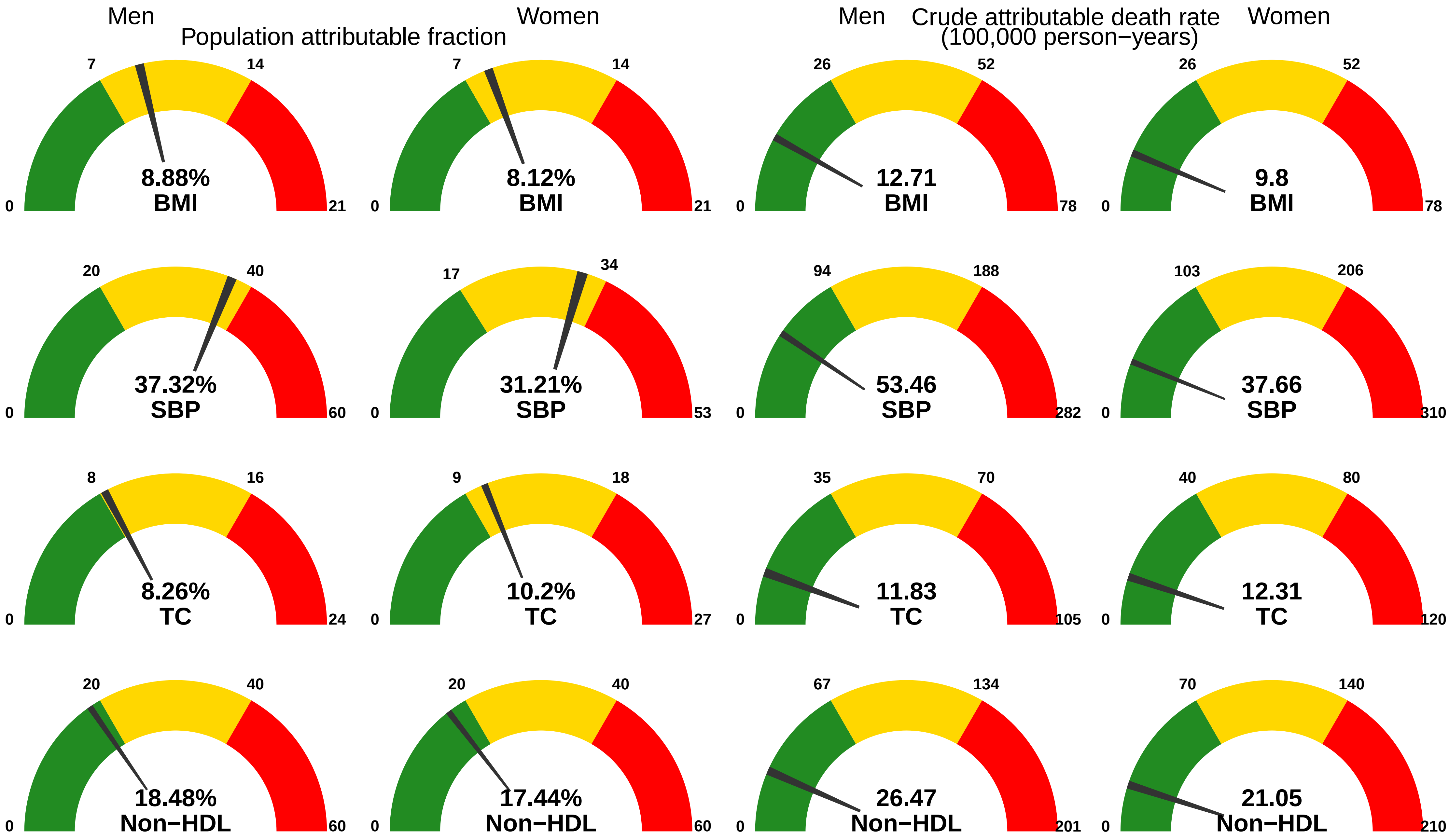

Puerto Rico

(Caribbean)

Legend: BMI = body mass index;
SBP = systolic blood pressure;
TC = total cholesterol;
Non−HDL = Non−HDL cholesterol.
Upper values are the largest
observed across countries,
risk factor− and sex−specific.
Sex− and age−specific results
are available through authors.

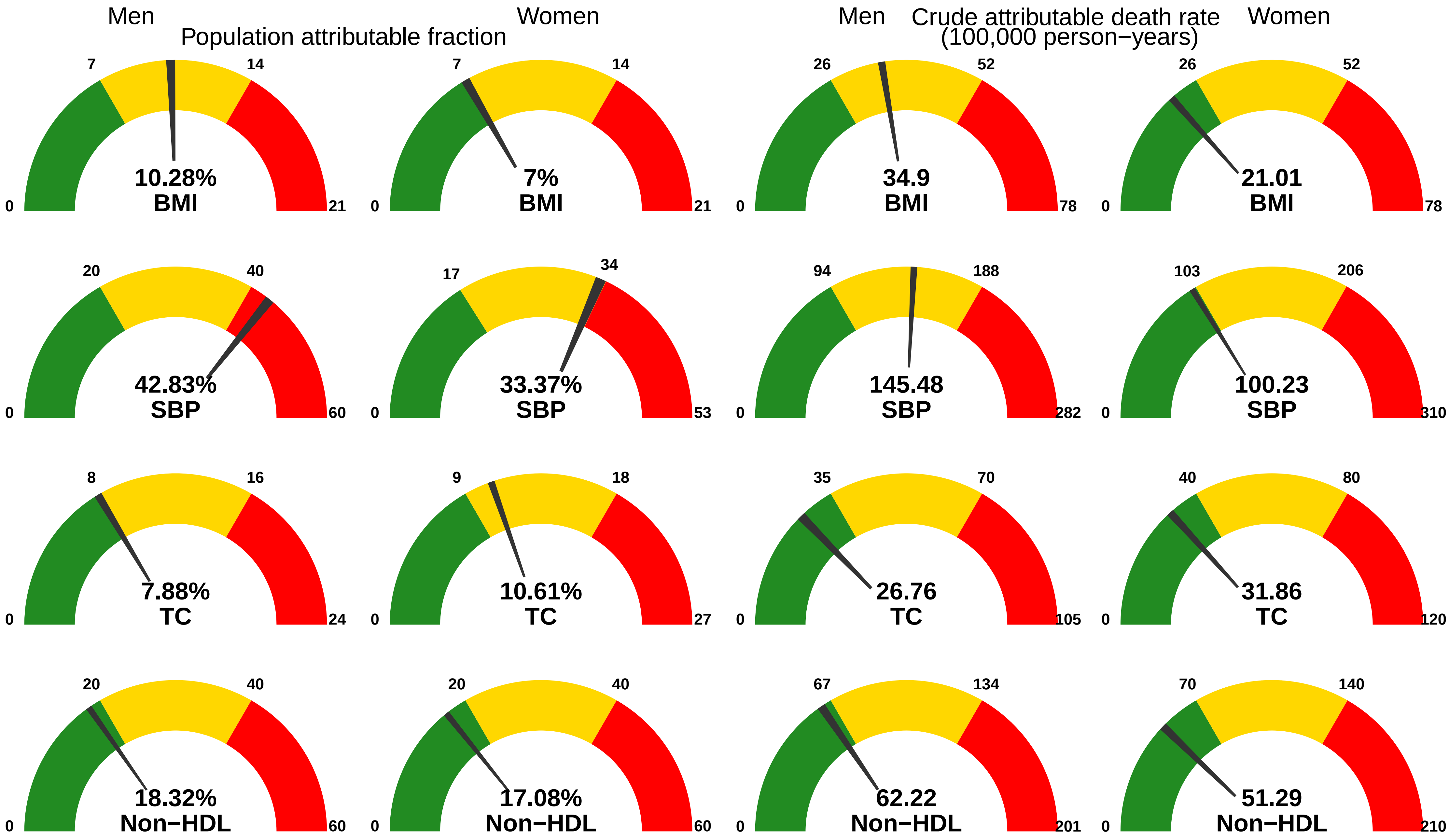

Saint Kitts and Nevis

(Caribbean)

Legend: BMI = body mass index;
SBP = systolic blood pressure;
TC = total cholesterol;
Non−HDL = Non−HDL cholesterol.
Upper values are the largest observed across countries, risk factor− and sex−specific.
Sex− and age−specific results are available through authors.

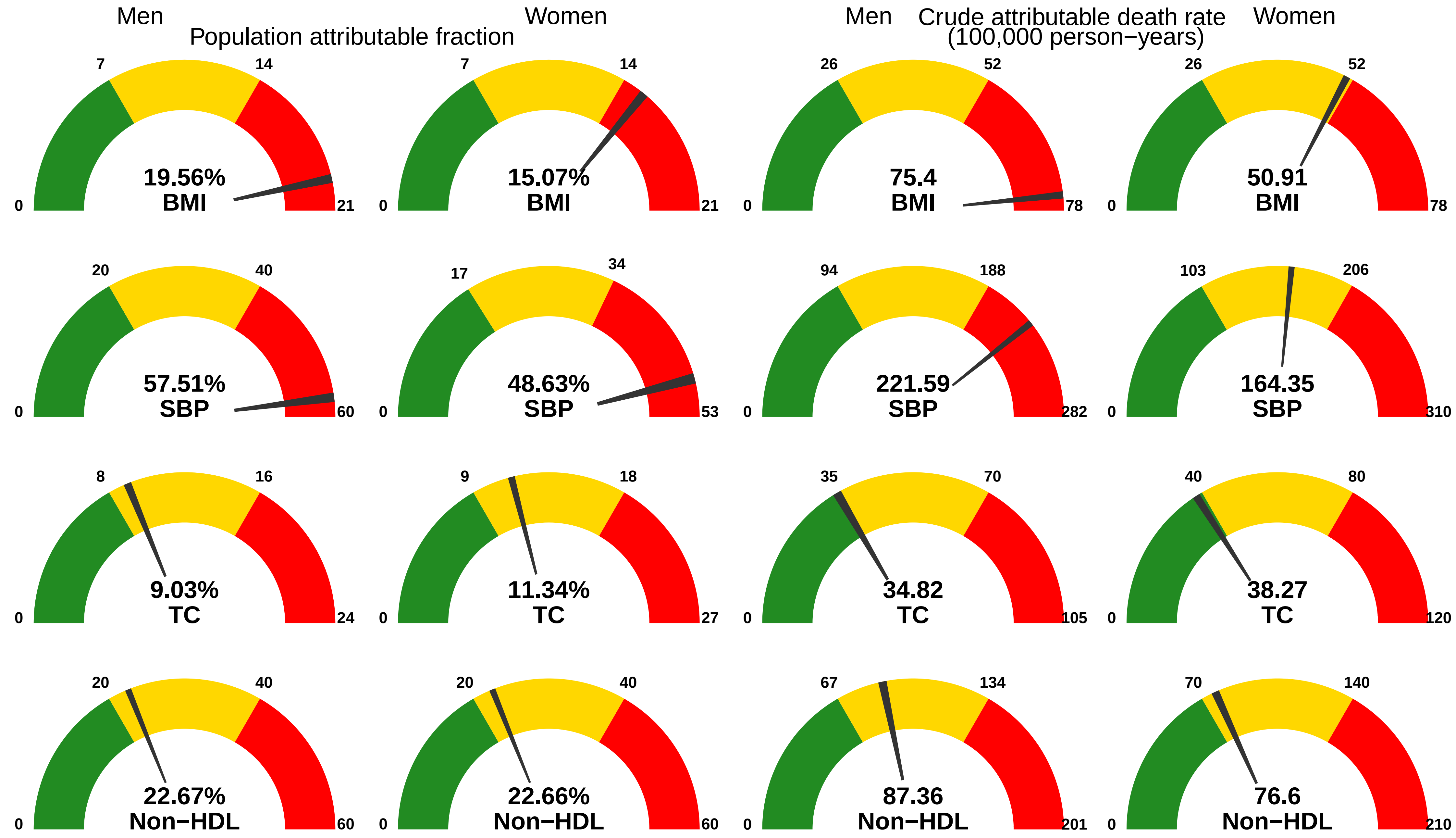

Saint Lucia

(Caribbean)

Legend: BMI = body mass index;
SBP = systolic blood pressure;
TC = total cholesterol;
Non−HDL = Non−HDL cholesterol.
Upper values are the largest observed across countries, risk factor− and sex−specific.
Sex− and age−specific results are available through authors.

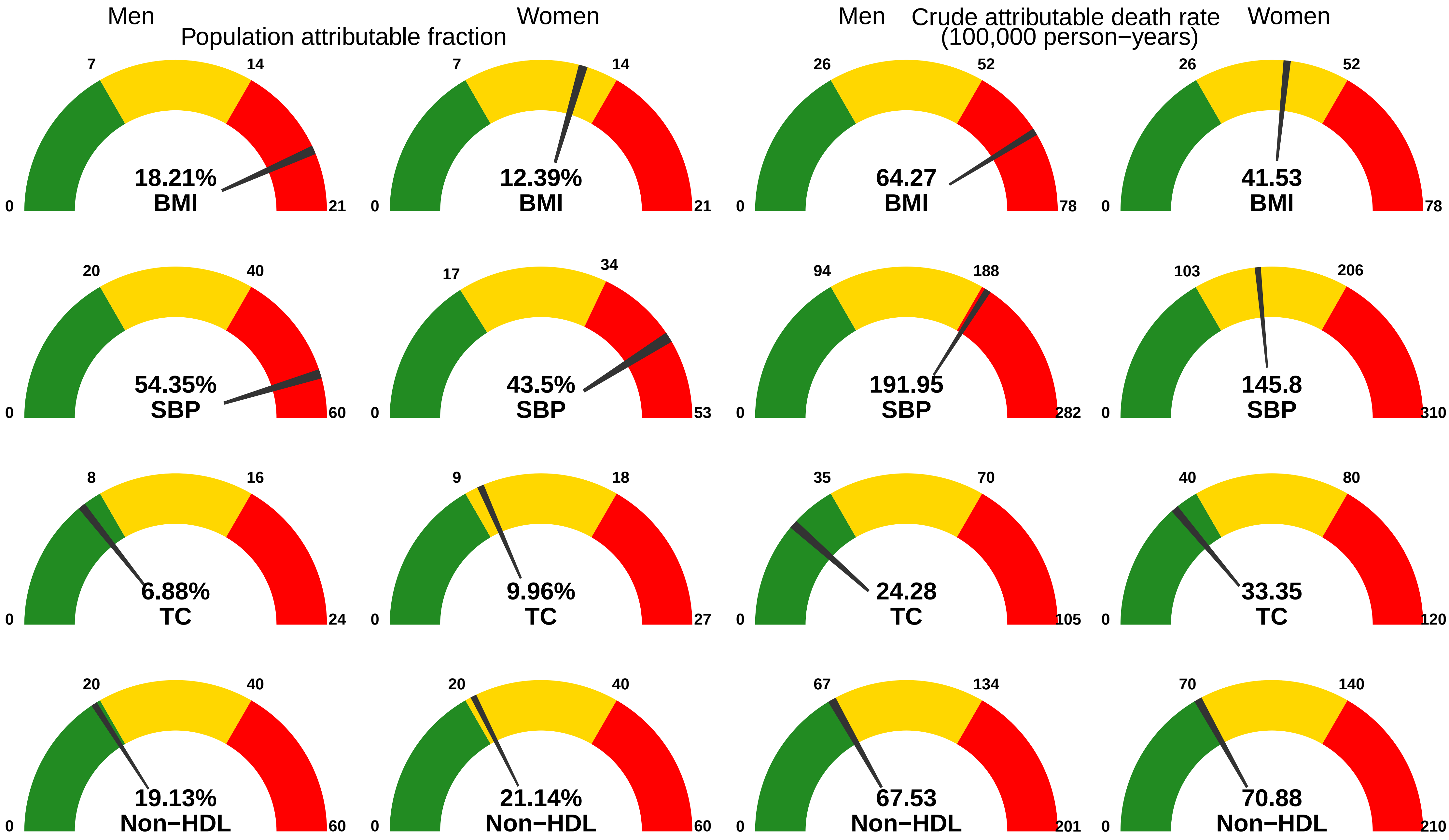

Saint Vincent and the Grenadines

(Caribbean)

Legend: BMI = body mass index;
SBP = systolic blood pressure;
TC = total cholesterol;
Non−HDL = Non−HDL cholesterol.
Upper values are the largest observed across countries, risk factor− and sex−specific.
Sex− and age−specific results are available through authors.

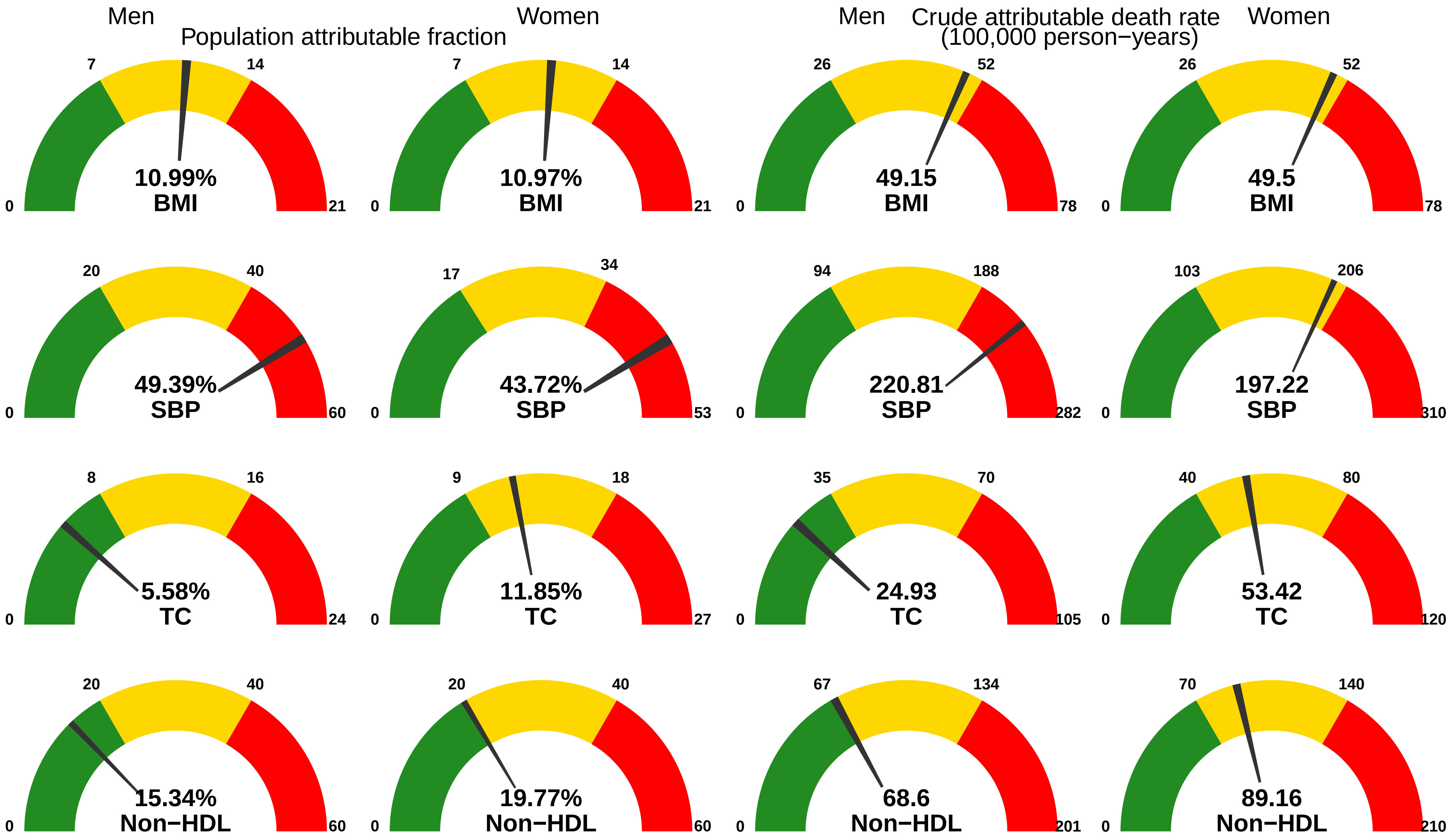

Suriname

(Caribbean)

Legend: BMI = body mass index;
SBP = systolic blood pressure;
TC = total cholesterol;
Non−HDL = Non−HDL cholesterol.
Upper values are the largest observed across countries, risk factor− and sex−specific.
Sex− and age−specific results are available through authors.

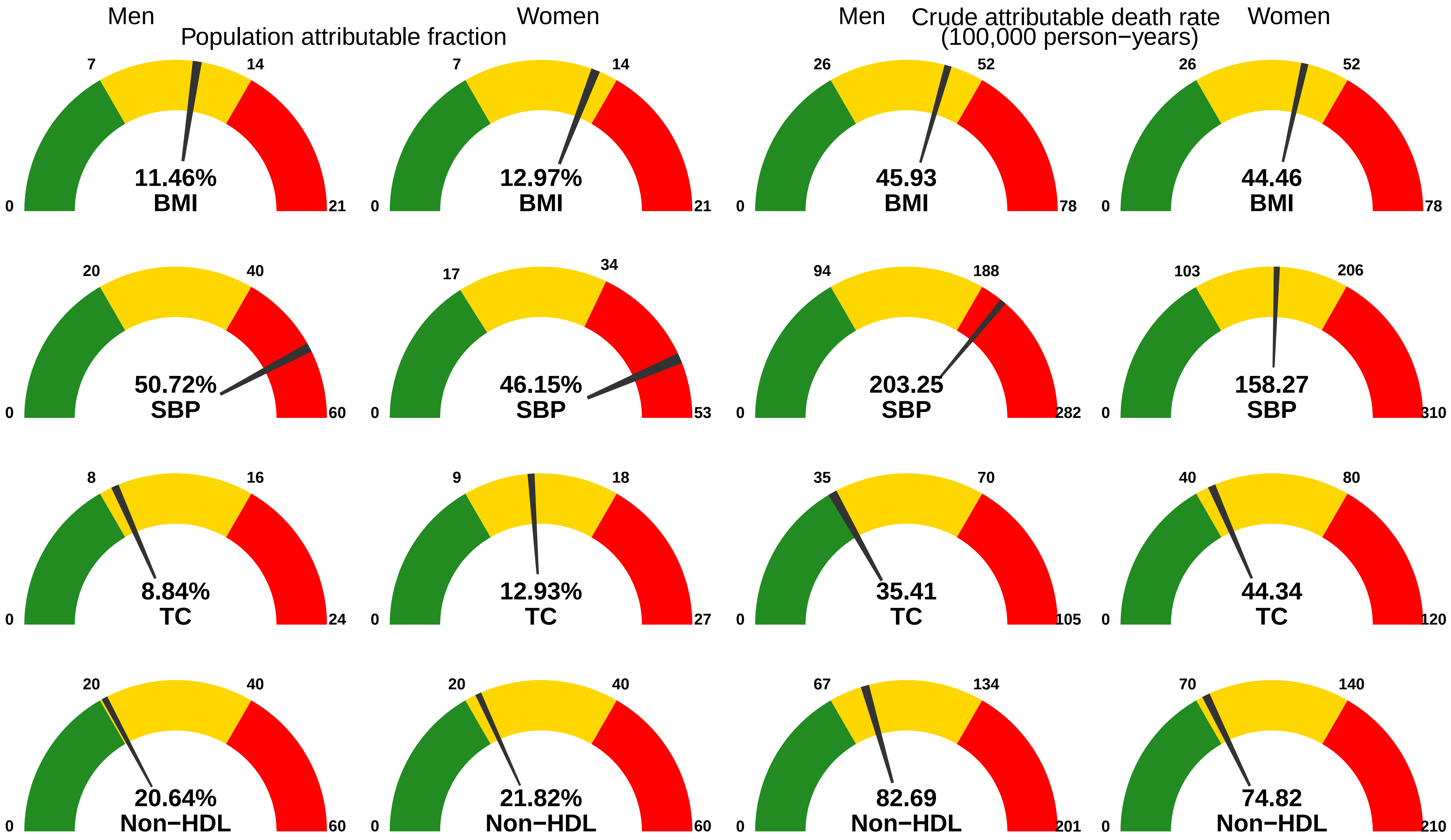

Trinidad and Tobago

(Caribbean)

Legend: BMI = body mass index;
SBP = systolic blood pressure;
TC = total cholesterol;
Non−HDL = Non−HDL cholesterol.
Upper values are the largest
observed across countries,
risk factor− and sex−specific.
Sex− and age−specific results
are available through authors.

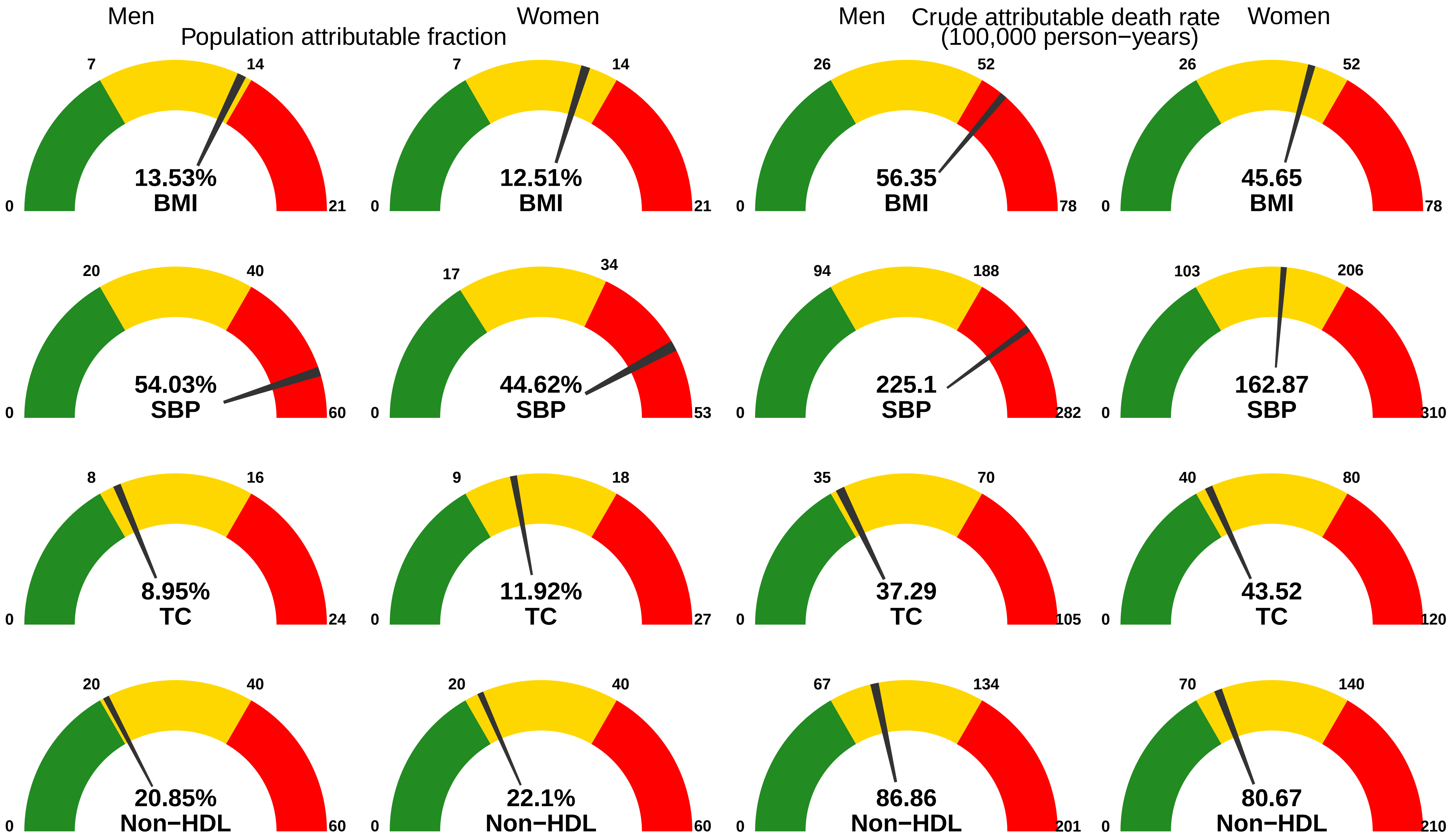

Uruguay

(Southern and Tropical Latin America)

Legend: BMI = body mass index;
SBP = systolic blood pressure;
TC = total cholesterol;
Non−HDL = Non−HDL cholesterol.
Upper values are the largest
observed across countries,
risk factor− and sex−specific.
Sex− and age−specific results
are available through authors.

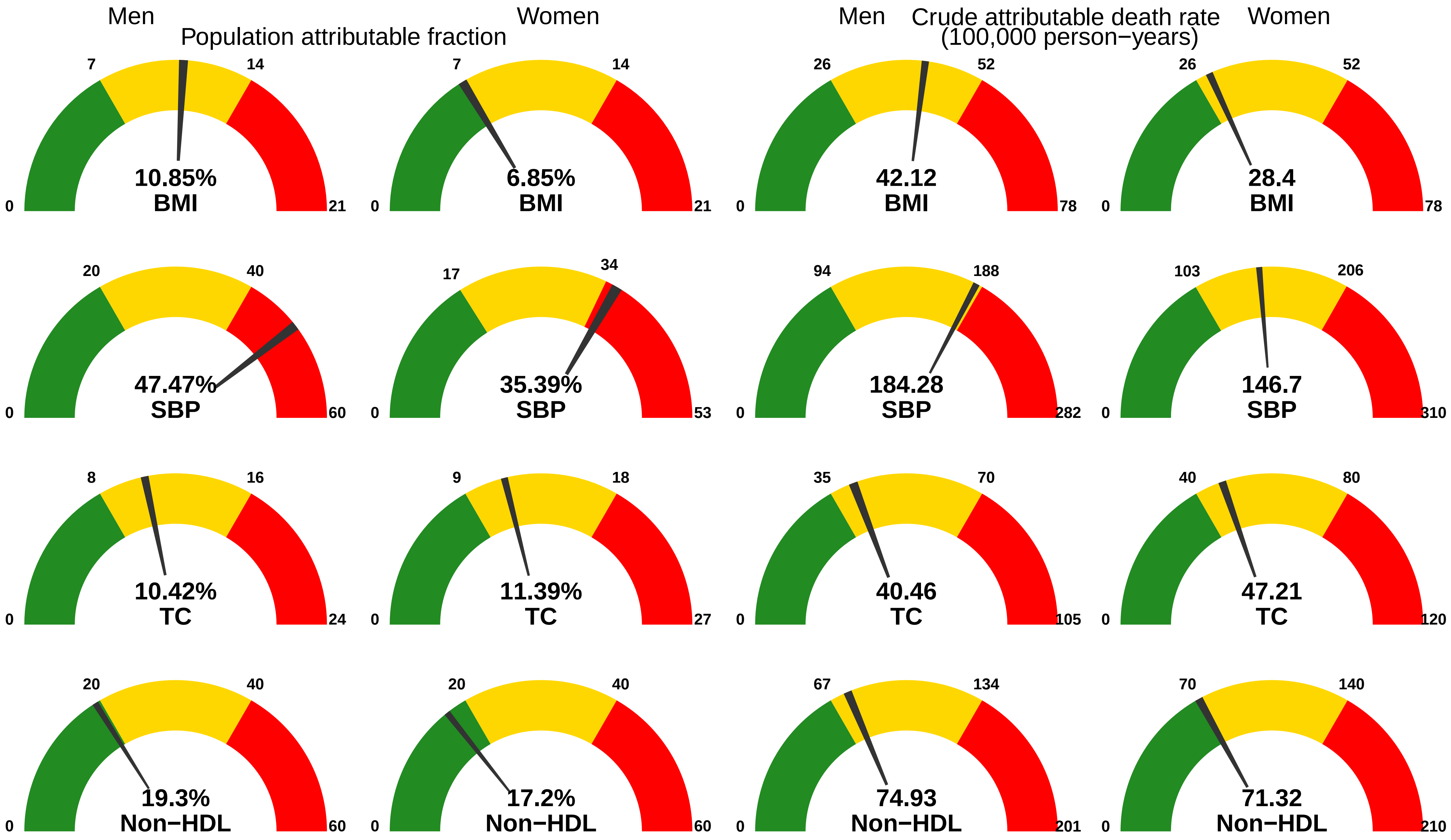

Venezuela

(Central Latin America)

Legend: BMI = body mass index;
SBP = systolic blood pressure;
TC = total cholesterol;
Non−HDL = Non−HDL cholesterol.
Upper values are the largest
observed across countries,
risk factor− and sex−specific.
Sex− and age−specific results
are available through authors.

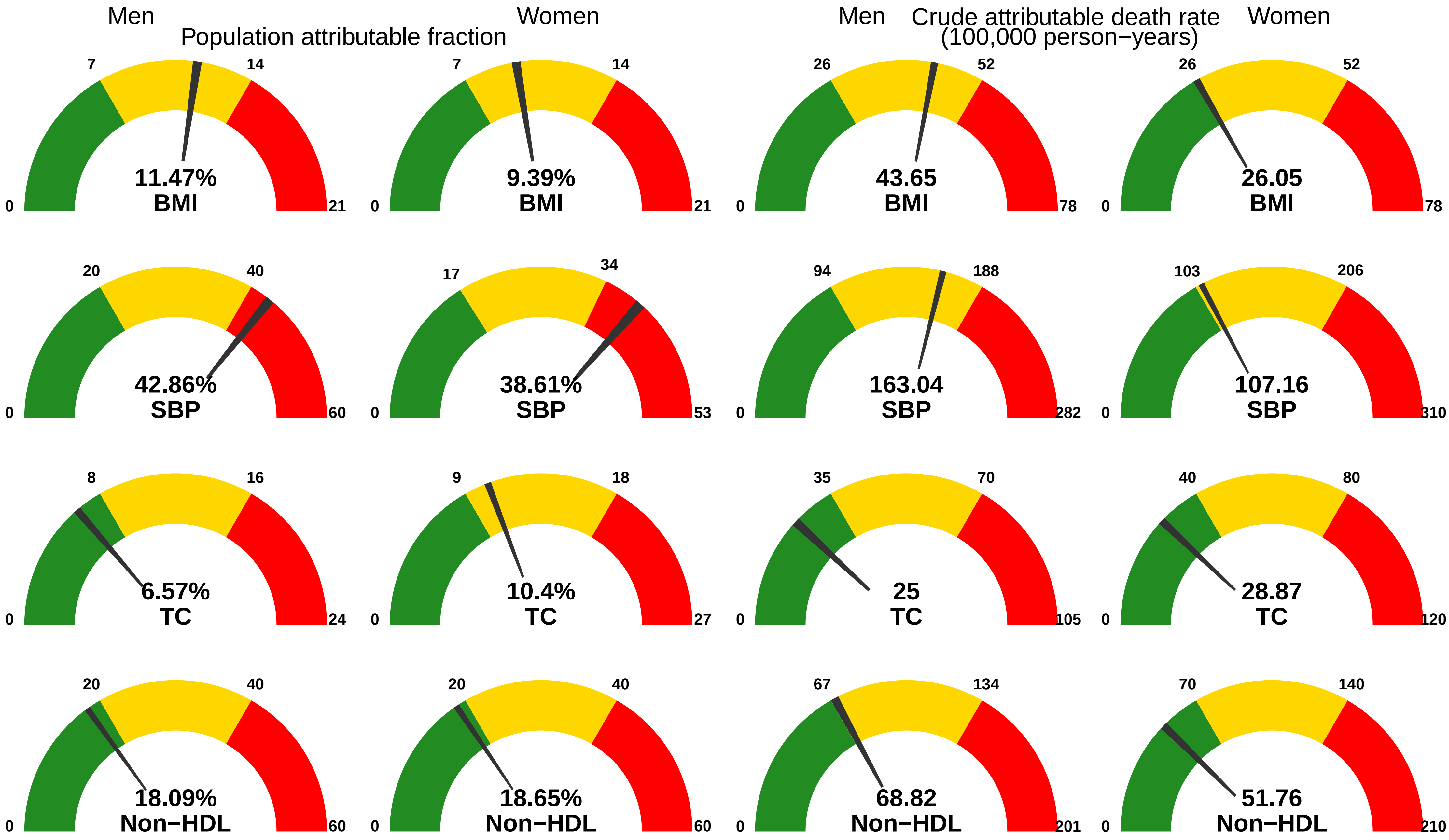

Supplement: Supplementary file 1 [file mmc1.pdf]
